# Comparison of left ventricular deformation abnormalities by echocardiography with cardiac magnetic resonance imaging in patients with acute myocarditis and preserved left ventricular ejection fraction

**DOI:** 10.3389/fcvm.2023.1322145

**Published:** 2024-01-09

**Authors:** Joscha Kandels, Sarah Richter, Andreas Hagendorff, Kristian Kragholm, Bhupendar Tayal, Ulrich Laufs, Timm Denecke, Stephan Stöbe

**Affiliations:** ^1^ Department of Cardiology, Leipzig University Hospital, Leipzig, Germany; ^2^ Department of Internal Medicine I, Martha-Maria Hospital Halle-Dölau, Halle (Saale), Germany; ^3^ Department of Cardiology, Aalborg University Hospital, Aalborg, Denmark; ^4^ Unit of Clinical Biostatistics and Epidemiology, Aalborg University Hospital, Aalborg, Denmark; ^5^ Houston Methodist DeBakey Heart and Vascular Center, Houston, TX, United States; ^6^ Department of Diagnostic and Interventional Radiology, University Hospital Leipzig, Leipzig, Germany

**Keywords:** transthoracic echocardiogram (TTE), acute myocarditis, deformation imaging, cardiac magnetic resonance imaging, longitudinal strain analysis, circumferential strain, T1-mapping, T2-mapping

## Abstract

**Purpose:**

Cardiac magnetic resonance imaging (cMRI) represents the gold standard to detect myocarditis. Left ventricular (LV) deformation imaging provides additional diagnostic options presumably exceeding conventional transthoracic echocardiography (TTE). The present study aimed to analyze the feasibility to detect myocarditis in patients (pts) with preserved LV ejection fraction (LVEF) by TTE compared to cMRI. It has been hypothesized that the number of pathological findings by deformation imaging correspond to findings in cMRI.

**Methods and results:**

Between January 2018 and February 2020 102 pts with acute myocarditis according to the modified Lake Louise criteria and early gadolinium enhancement (EGE) by cMRI were identified at the department of cardiology at the University Hospital Leipzig. Twenty-six pts were included in this retrospective comparative study based on specific selection criteria. Twelve pts with normal cMRI served as a control group. LV deformation was analyzed by global and regional longitudinal strain (GLS, rLS), global and regional circumferential and radial strain (GCS, rCS, GRS, rRS), and LV rotation (including layer strain analysis). All parameters were compared to findings of edema, inflammation, and fibrosis by cMRI according to Lake Louise criteria. All pts with acute myocarditis diagnosed by cMRI showed pathological findings in TTE. Especially rCS and LV rotation analyzed by regional layer strain exhibit a high concordance with pathological findings in cMRI. In controls no LV deformation abnormalities were documented. Mean values of GLS, GRS, and GCS were not significantly different between pts with acute myocarditis and controls.

**Conclusion:**

This retrospective analysis documents the feasibility of detecting regional deformation abnormalities by echocardiography in patients with acute myocarditis confirmed by cMRI. The detection of pathological findings due to myocarditis requires the determination of regional deformation parameters, particularly rCS and LV rotation. The assessment of global strain values does not appear to be of critical value.

## Introduction

Acute myocarditis may manifest clinically in several ways (1). Symptoms range from asymptomatic patients (pts) to those with mild chest pain and palpitations to manifestation of cardiogenic shock with severely reduced left ventricular (LV) ejection fraction (LVEF) and malignant arrhythmia (2, 3). Acute myocarditis with reduced LVEF can be easily detected by transthoracic echocardiography (TTE) and confirmed by cardiac magnetic resonance imaging (cMRI) representing the gold standard (4, 5). In cMRI non-ischemic myocardial inflammation is characterized by the modified Lake Louise criteria [main criteria: myocardial edema and non-ischemic myocardial injury by T1-mapping, extracellular volume, and late gadolinium enhancement (LGE)] and early gadolinium enhancement (EGE) (4, 5). Evidence of myocarditis in pts with preserved LVEF remains challenging (6, 7). Thus, speckle tracking echocardiography (STE) analyzing LV deformation might be suitable to detect myocardial involvement of oligosymptomatic myocarditis exposing subclinical impairment of LV function (8–14). Following the acute stage of myocarditis, pts are at ongoing risk of fatal arrhythmic events, dilated cardiomyopathy, and death compared to the general population (1, 3, 15). Due to the increased mortality risk—even after mild courses of acute myocarditis—residual structural changes representing arrhythmogenic substrates, may be detected by regional deformation abnormalities with the aid of STE (8, 10, 11, 16). The aim of the present study was to investigate the feasibility of STE to detect abnormalities of LV deformation components in pts with acute myocarditis with preserved LVEF confirmed by cMRI.

## Methods

### Study population

**Figure 1:**
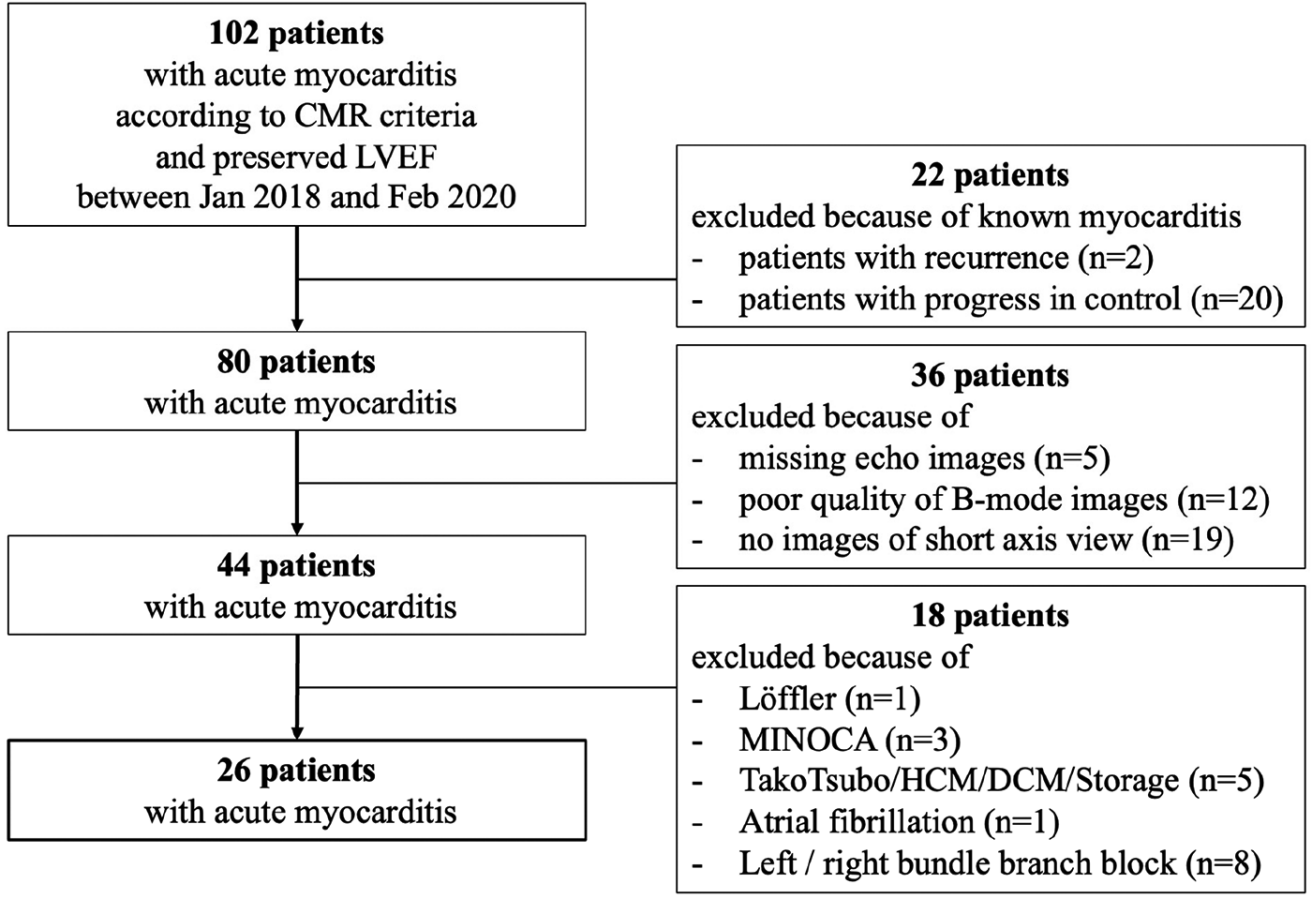
Flowchart of patient selection for analysis of pts with acute myocarditis with preserved LVEF and sinus rhythm.

In this retrospective study, 102 pts with acute myocarditis based on the modified Lake Louise criteria using cMRI were considered between January 2018 and February 2020 at the University Hospital Leipzig. Coronary artery disease was excluded by coronary angiography in all pts. Exclusion criteria of the present study are illustrated in Figure 1. Thus, the comparison between tissue characterization by cMRI and abnormalities of LV deformation components detected by STE has been performed in a highly selected cohort. As the diagnosis of myocarditis on MRI is currently based on the Lake Louise criteria and T1 and T2 mapping analyses, and not on functional impairment of LV deformation, this study compared tissue changes as determined by tissue characterization on CMR with regional functional changes of the different components of LV deformation by echocardiography.

A control group consisted of pts with complete echocardiographic and CMR data available at the same period as the myocarditis pts without showing any clinical, laboratory or imaging evidence of myocarditis. They were formed by a group of pts who had undergone a diagnosis of exclusion of myocardial ischemia, which may explain the increased number of women. The retrospective study was conducted in accordance with the Declaration of Helsinki and was approved by the ethical committee of the University of Leipzig (**359/18-ek**). All included pts provided informed consent.

### Transthoracic echocardiography (TTE)

TTE was performed using a Vivid E9 or E95 ultrasound systems with a M5-S or a 4Vc phased array probe (GE Healthcare Vingmed Ultrasound AS, Horten, Norway). Analyses were performed with the EchoPac software (Version 204, GE Healthcare Vingmed Ultrasound AS, Horten, Norway).

Conventional echocardiographic parameters, e.g., relative wall thickness (RWT), LV mass index (LVMi), LVEF, and cardiac index (CI) were determined by 2D and Doppler echocardiography according to current guidelines and recommended reference values were applied (17–19). All components of LV deformation were analyzed. 2D speckle tracking of the apical long-axis-, two- and four-chamber-view (2-ChV, 4Ch-V) was performed to characterize global longitudinal peak-systolic strain (GLS) and regional longitudinal strain (rLS) using the 18 LV segment model according to current recommendations (20, 21). For GLS and rLS assessment full myocardial tracking was performed after manual adjustment and acceptance of LV segments with accurate tracking. Global circumferential strain (GCS) and regional circumferential strain (rCS) as well as global radial strain (GRS) and regional radial strain (rRS) were assessed by full myocardial speckle tracking based on apical, mid, and basal parasternal short axis views (22–24). The acquisition of parasternal short-axis views requires a high degree of standardization. In particular, the basal and apical short-axis views must be assigned to segments based on the biplane perpendicular long-axis alignment (17). In addition, apical and basal LV rotation was analyzed. Artefact tracking was excluded according to recent recommendations (17, 21). Currently recommended values were used as reference values for longitudinal, circumferential, and radial strain (25, 26). Pathological strain patterns were characterized according to previously established criteria (17).

### Cardiac magnetic resonance imaging

cMRI was performed using a 3 Tesla Philips Achieva (Philips, Eindhoven, The Netherlands). Analyses were performed with the IntelliSpace PACS software (Version 4.4, Philips, Eindhoven, The Netherlands). LV volumes and LVEF were analyzed by LV planimetry, myocardial edema was detected by increased T2-STIR-ratio (STIR = short tau inversion recovery) and by increased values of native T2-mapping (reference: <45 ms) (5, 4, 27). Hyperemia was assessed by T1 black blood sequences prior and post gadolinium administration by EGE (5). EGE measurements were performed by left ventricular segmentation into cranial, mid, and caudal slices according to the first methodological description (5). Increased values of native T1mapping (reference: <1,250 ms) characterizes myocardial injury by an underlying disease processes (e.g., fibrosis, edema, deposits of storage diseases, and/or myocyte necrosis) (4, 27). Myocardial fibrosis was determined by the occurrence of late gadolinium enhancement (LGE) by SPIR (spectral presaturation with inversion recovery) and SCAR sequences (T1 weighted scouting lock locker sequences) (4, 5). As this paper compared echo findings with MRI findings, the labelling of the left ventricular segments primarily referred to the common standard of speckle tracking echocardiography using the 18-segment model (20, 21). Therefore, the 18-segment model was chosen for both the speckle tracking analyses and the description of the CMR findings to allow a consistent comparison of the segments.

### Statistical analysis

All statistical analyses were performed using SPSS Statistics (version 24.0, IBM, Armonk, NY) and Microsoft Office Excel (version 16.75, Microsoft). Normal data distribution was assessed by Kolmogorov-Smirnov-test. Continuous variables are expressed as mean ± standard deviation (SD). For continuous variables, differences between two groups were analyzed by student's t-test. The kappa coefficient (*κ*) was used to assess intra- and interobserver variability for echocardiographic measurements in 20 randomly selected pts. Data comparisons between more than two groups were performed by one-way analysis of Variance (ANOVA). All categorical variables were expressed as numbers and/or percentages. Chi-squared or Fisher exact test was used to analyze categorical variables as appropriate. A *P* value < 0.05 was considered to indicate statistical significance.

## Results

### Baseline characteristics and conventional echocardiographic parameters of systolic LV function

In 26 acute myocarditis pts with preserved LVEF (54% male, mean age 40.3 ± 13.3 years) pathological findings of LV deformation imaging by STE were compared with cMRI. Baseline characteristics of pts with acute myocarditis (*n* = 26) and controls (*n* = 12) are displayed in Table 1. Conventional echocardiographic parameters are shown in Table 2. Myocardial biopsy was performed in 7/26 (27%) pts. A pathogen was detected in 4/7 biopsies [EBV (2 pts), parvo B19, streptococcus].

**Table 1:** Baseline characteristics of acute myocarditis patients and controls.

Parameters	Myocarditis (*n* = 26)	Controls (*n* = 12)	*P* value
	Mean value ± SD	Mean value ± SD	
Age (years)	40.3 ± 13.3	32.1 ± 14.1	0.100
Sex (male)	14 (54%)	11 (92%)	0.023
Weight (kg)	79.4 ± 16.4	81.0 ± 18.5	0.798
Height (cm)	175.2 ± 9.3	183.5 ± 18.7	0.165
BMI (kg/m^2^)	27.3 ± 4.1	23.8 ± 3.6	0.012
HR (bpm)	77.1 ± 15.2	63.3 ± 6.7	<0.001
BPs (mmHg)	121.1 ± 14.4	125.9 ± 7.3	0.177
BPd (mmHg)	77.2 ± 15.0	72.6 ± 8.3	0.235

*P* value < 0.05 statistically significant.

BMI, body-mass-index; HR, heart rate; BPs, systolic blood pressure; BPd, diastolic blood pressure.

**Table 2:** Conventional echocardiographic parameters of acute myocarditis patients and controls.

Parameters	Myocarditis (*n* = 26)	Controls (*n* = 12)	*P* value
	Mean value ± SD	Mean value ± SD	
LVEF (%)	52.7 ± 8.2	63.0 ± 4.4	<0.001
GLS (%)	−17.2 ± 5.3	−19.3 ± 2.0	0.105
IVSD (mm)	9.8 ± 1.2	9.4 ± 1.6	0.458
RWT	0.42 ± 0.07	0.37 ± 0.05	0.016
LVEDDi (mm/m^2^)	24.2 ± 2.1	25.6 ± 2.9	0.077
LVMi male (g/m^2^)	96.3 ± 17.2	85.3 ± 13.3	0.037
LVMi female (g/m^2^)	90.1 ± 17.9	77.0 ± 0	<0.001
E/E	7.4 ± 2.0	6.3 ± 1.9	0.113
E/A	1.3 ± 0.4	1.3 ± 0.6	1
sPAP (mmHg)	28.5 ± 7.3	26.9 ± 3.5	0.369

*P* value < 0.05 statistically significant.

LVEF, left ventricular ejection fraction; GLS, global longitudinal strain; IVSD, interventricular septum diameter; RWT, relative wall thickness; LVEDD, left ventricular end-diastolic diameter indexed; LVMi, left ventricular mass index; sPAP, systolic pulmonary arterial pressure.

### Heterogeneous patterns of pathological findings in acute myocarditis pts with preserved LVEF

**Figure 2:**
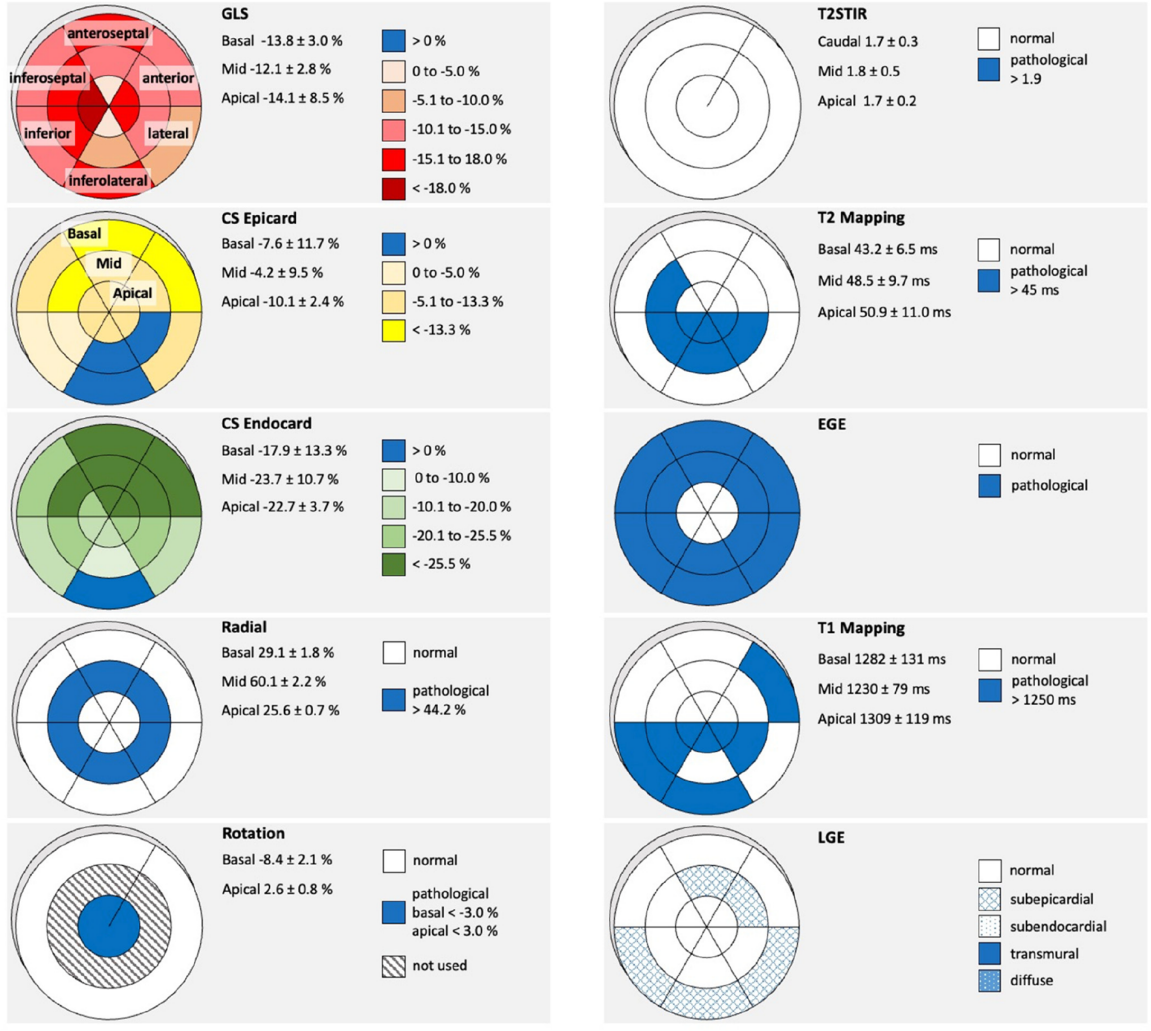
Pathological findings by cMRI and STE in a representative patient with acute myocarditis. On the right-side bull's eye schemes of T2-STIR **(**short tau inversion recovery), native T2-mapping, EGE (early gadolinium enhancement), native T1-Mapping, LGE (late gadolinium enhancement) are shown. On the left side bull's eye schemes of rLS (regional longitudinal strain), subepicardial CS (circumferential strain), subendocardial CS, RS (radial Strain), and LV rotation (basal and apical LV rotation in degrees) are shown. The corresponding LV segments and the level of the LV are designated in fist two bull's eye schemes as an example. Legends and respective reference values are given next to the corresponding bull's eye schemes.

All pathological findings were evaluated individually regarding their localization, extent, and severity. In Figure 2 schemes of the pathological findings are illustrated by an example of a representative myocarditis patient. Qualitative findings were characterized as non-pathological or pathological regarding the 18-segment model. Quantitative values were described as pathological by exceeding the reference values. Qualitative findings of LGE were characterized by different patterns of LV segments according to their different pathology (non-pathological, subepicardial, subendocardial, transmural, diffuse). Pathological findings of longitudinal and circumferential strain were characterized by different color brightness and colors according to their %-change-intervals. Radial strain and LV rotation were described as pathological by exceeding reference values.

**Figure 3:**
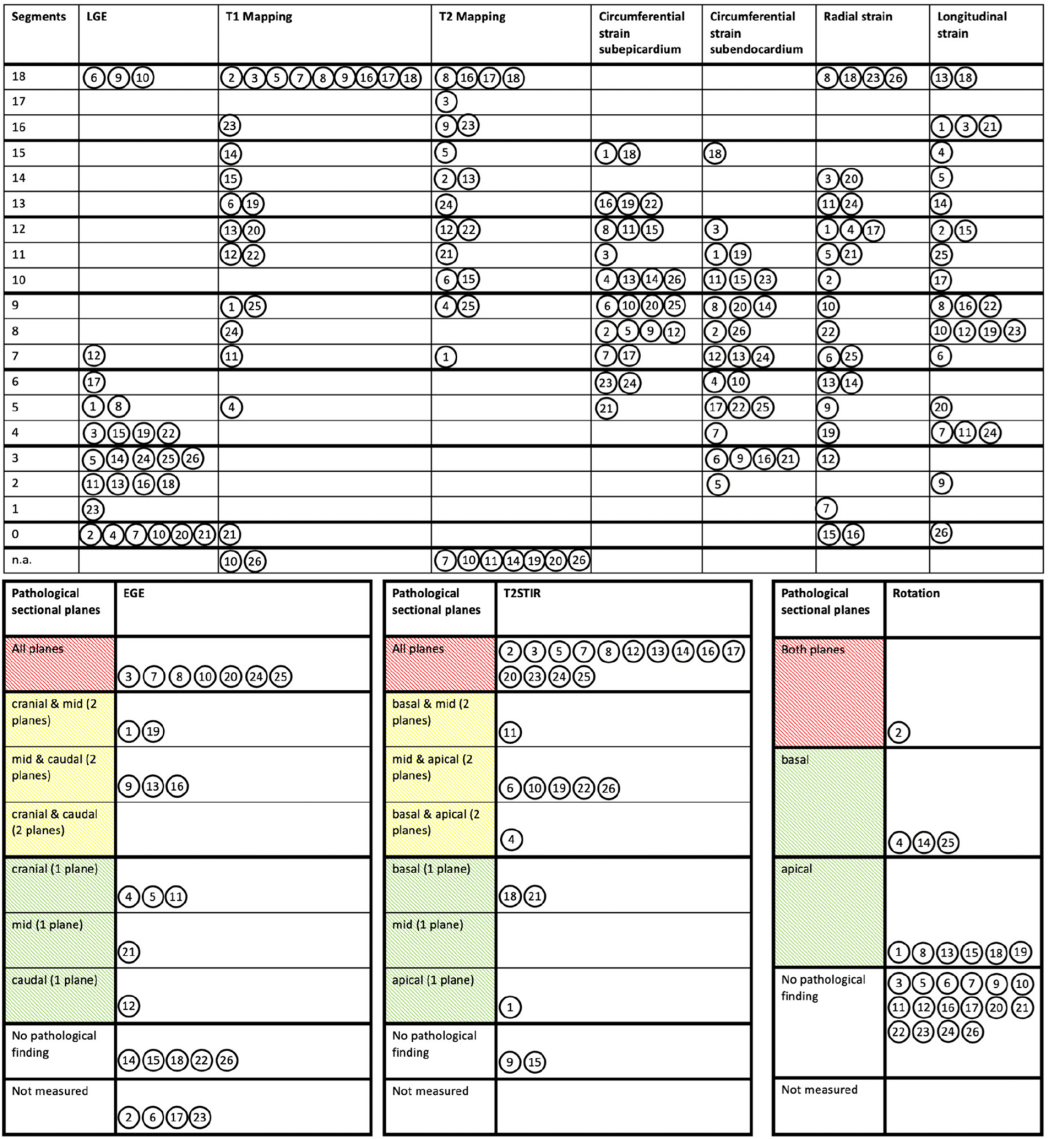
Variable distribution and severity of involved pathological segments documented by cardiac magnetic resonance imaging and speckle tracking echocardiography. Pathological findings of LGE (late gadolinium enhancement), native T1- and T2-Mapping, subepi- and subendomyocardial circumferential strain, radial and longitudinal strain were assigned to the respective 18 LV segments. Pathological EGE (early gadolinium enhancement), T2-STIR (short tau inversion recovery) and LV rotation were assigned to the respective three LV levels (basal, mid, and apical). The circled number represents the corresponding patient in each case. n.a., not available.

The localizations of the individually variable pathological findings are shown in Figure 3. In general, LV deformation parameters showed patchy patterns. Predominant alterations of LV deformation were documented in the basal inferolateral and neighboring LV segments (subepicardial CS) with consecutive effects on LV rotation (Figure 3). LV deformation abnormalities by STE could be observed in all pts within or near the segments that showed suspicious abnormalities of tissue characterization by cMRI. For transparency and verifiability of the detected pathologies, the individual schemes for each patient are represented in the Supplementary Material.

**Figure 4:**
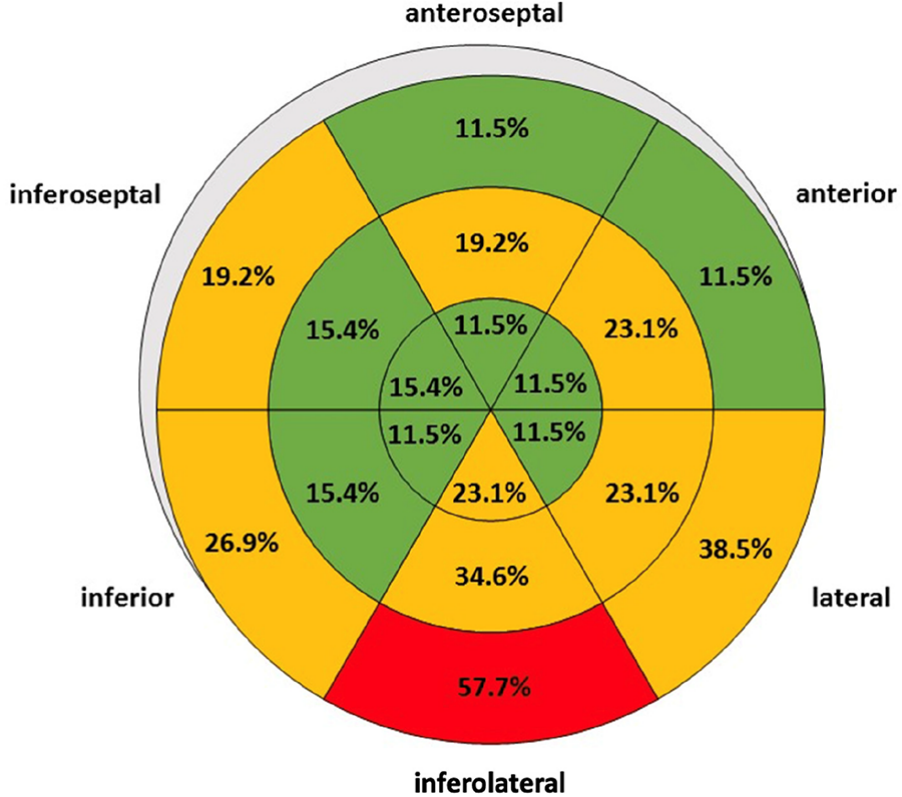
Distribution of regional late gadolinium enhancement in patients with acute myocarditis illustrated by a bull's eye plot. LV segments with less than 5 positive findings out of 26 patients are labelled in green, 5 to 10 positive findings out of 26 patients in orange, and more than 10 positive findings out of 26 patients in red.

Mean values of T2-STIR ratios and native T2-mapping were increased documenting myocardial edema (Supplementary Table S1). Especially, the basal inferolateral LV segment showed higher native T2-mapping values (47.0 ± 5.9 vs. 51.7 ± 6.7; *P* = 0.02). Increased EGE due to hyperemia was documented in half of the pts (Supplementary Table S1). Native T1-mapping values of the basal inferolateral LV segments were significantly higher compared to the apical segments (1,290.0 ± 80.7 vs. 1,324.4 ± 85.0; *P* = 0.03) (Supplementary Table S1). The distribution of LGE was individually variable being pronounced in the basal inferolateral LV segments (Figure 4).

STE findings with respect to the localization of the involved LV segments are illustrated in the Supplementary Table S1. rLS was within lower normal ranges in the basal-mid LV segments and in normal ranges in the apical LV segments. rLS of the basal inferoseptal (−15.5 ± 2.7% vs. −21.7 ± 6.6%; *P* < 0.001) and inferior (−18.0 ± 3.8% vs. −22.8 ± 6.3%; *P* = 0.002) LV segments were reduced compared to the apex. Subepicardial CS was predominantly reduced in the anterior LV segments. Significantly lower subepicardial CS could be observed in the basal inferolateral (−5.5 ± 10.9% vs. −12.8 ± 9.5%; *P* = 0.039), inferoseptal (−17.0 ± 7.2% vs. −10.4 ± 7.2%; *P* < 0.001), and anteroseptal LV segments (−18.9 ± 7.7% vs. −9.0 ± 6.9%; *P* < 0.001) compared to corresponding apical segments. Subendocardial CS was within lower normal values. Significantly lower subendocardial CS could be observed in the basal anteroseptal LV segments (−34.5 ± 9.9% vs. −24.6 ± 11.0%; *P* < 0.001) compared to corresponding apical segments. RS was within normal ranges without any significant differences between LV apex and base (Supplementary Table S1). Regional LV rotation was in normal ranges, although LV rotation was higher in all segments of LV apex (*P* < 0.001) (Supplementary Table S2).

In the control group of pts who underwent cMRI due to unspecific symptoms, no pathological findings were documented by cMRI. Accordingly, all components and layers of LV deformation showed normal values by STE (Supplementary Table S3).

Intra- and interobserver variability showed a high agreement for echocardiographic longitudinal (*κ* = 0.86; *z* = 4.58, *p* < 0.001; *κ* = 0.79; *z* = 4.28, *p* < 0.001) and circumferential strain measurements (*κ* = 0.83; *z* = 4.55, *p* < 0.001; *κ* = 0.78; *z* = 4.19, *p* < 0.001) in 20 randomly selected pts. Intra- and interobserver variabilities for the remaining conventional echocardiographic measurements consistently showed good agreement as well.

## Discussion

The main findings of the present study about acute myocarditis pts with preserved LVEF are:

(1) Findings of edema or/and myocardial injury documented by cMRI are associated with impairment of LV deformation components documented by STE.
(2) The variable distribution of involved LV segments show a patchy pattern in acute myocarditis pts documented by both, cMRI and STE.
(3) Regional LV involvement due to acute myocarditis (edema, subepicardial or diffuse myocardial injury) documented by cMRI does not always lead to impairments of LV deformation in the same LV segments. However, at least neighboring LV segments are involved.
(4) LGE is predominantly observed by cMRI in the basal inferolateral LV segment.
(5) Impairment of LV deformation is predominantly observed due to decreased rCS by STE in the basal inferolateral LV segment.
(6) In controls no pathological findings were observed neither by cMRI nor by STE.

### Diagnostic challenge of acute myocarditis

Histopathological findings of myocardial inflammation in endomyocardial biopsy (EMB) specimens are considered the gold standard for detecting (acute) myocarditis (7, 28). However, the diagnosis can only be confirmed by EMB if signs of inflammation or the virus itself are detected in the specimens. cMRI represents the accepted non-invasive gold standard to diagnose acute myocarditis (4–7). Diagnostic targets of cMRI comprise hyperemia, capillary leak, edema, myocardial injury by loss of cell integrity, necrosis, infiltration of inflammatory cells like macrophages as well as interstitial fibrosis and scars. Although these changes due to myocardial tissue inflammation cannot define the final specific diagnosis, cMRI can predict the diagnosis with high probability based on tissue phenotyping and distribution of LGE pattern (29).

Echocardiography is usually mentioned in the guidelines for the diagnosis of acute myocarditis only to detect left ventricular systolic function or to first rule out other causes of heart failure, e.g., valvular heart diseases, congenital heart disease or certain types of cardiomyopathies (28, 30). It can further be used to monitor LV systolic function including wall motion abnormalities, pericardial effusion, or intracavitary thrombi (7, 28, 31). In contrast to the detection of specific structural tissue changes by cMRI, echocardiography—especially in acute myocarditis with preserved LVEF—is limited by the sole detection of functional findings, which allow interpretation only in context of the patient's medical history and clinical situation. Despite the increased number of women and the lower BMI of the control group, it seems unlikely to the authors that these factors are likely to be relevant in the diagnosis of myocarditis. In the present study, LVEF is significantly lower in myocarditis pts than in controls, although the mean value is in the lower normal range. However, this finding is not surprising, as the patchy and predominantly subepicardial fibrosis observed may impair the functional state of the LV myocardium. According to the available data, changes in LVEF seem to affect the circumferential component of LV deformation more than the longitudinal component.

STE may improve the echocardiographic diagnostic approach in acute myocarditis by objectifying both discrete regional wall motion abnormalities and involvement of the different myocardial LV layers (20, 22, 25, 26, 32–36). The analysis of different components of LV deformation, including regional myocardial layers, may be particularly useful in the diagnosis of myocarditis because subepicardial myocardial damage is predominantly seen (5, 6). Currently, STE is increasingly used to support the suspected diagnosis of acute myocarditis (8, 11–14). However, 2D STE requires standardized imaging with sufficient image quality to perform tracking analyses in a valid and verifiable manner without artifact tracking (17, 24, 37). In addition, inter-vendor differences of tracking analyses must be considered (38).

Of note, despite the small number of pts with acute myocarditis, pathological findings were found by both cMRI and STE in all pts, whereas no pathological findings were found by either cMRI or STE in controls during this time. However, this small cohort of pts from the pre-SARS-CoV-2 era seems noteworthy, as pts after SARS-CoV-2 infection often present with cardiac symptoms suggestive for concomitant myocardial involvement (16, 39–42). Because residual cardiac findings after unrecognized concomitant myocarditis secondary to SARS-CoV-2 infection are likely to cause changes in LV rotation, even this highly selected group of pre-SARS-CoV-2 era pts may be valuable for future investigation.

### The association between structural changes and functional impairment in acute myocarditis

Edema, myocardial injury and fibrosis due to inflammation, as well as vascular complications due to acute inflammation-related thrombus formation, may affect all LV regions to varying degrees (1, 7). While acute myocarditis with severe heart failure is easily recognized by functional abnormalities using TTE and STE, mild courses of myocarditis are often underdiagnosed, although these pts also appear to be associated with a less favorable prognosis (1, 3, 7, 8, 13). The present data demonstrate an association between abnormalities in tissue characterization documented by cMRI and impairment of LV deformation documented by STE. In this regard, there is a relevant degree of myocardial involvement in pts with acute myocarditis characterized by pathological CS, without apparent impairment of global LV systolic function. However, impairment of LV deformation by STE did not always correspond to LV segments with abnormalities in tissue characterization according to cMRI. Thus, in individual pts, LV deformation was unremarkable in LV segments where myocardial edema was detectable on cMRI. Even in pts with subepicardial LGE, rCS could still be within normal ranges. An additional involvement of the pericardium in the presence of perimyocarditis is usually manifested by an accompanying pericardial effusion. Adhesion between the epicardium and pericardium after absorption of pericardial fluid could explain the functional impairment of LV segments adjacent to LGE findings detected by cMRI in prolonged inflammation or chronic courses. The most striking finding was pathological rCS in the subepicardial layers, which was predominantly detected in the basal inferolateral LV segments. Thus, impairment of LV rotation is apparently more frequently affected than impairment of rLS. Based on this higher sensitivity the use of STE to detect subclinical myocardial involvement in acute myocarditis seems reasonable.

### The attribution of functional impairments secondary to acute myocarditis

The observed pathological patterns of the individual LV deformation components cannot be exclusively attributed to acute myocarditis, as they represent nonspecific findings with a variety of possible causes. However, especially in younger pts without previous cardiac disease, the suspected diagnosis of myocarditis can be corroborated by these STE modalities. Follow-up examinations are frequently performed in special cohorts such as athletes and/or special occupational groups such as pilots. In these situations, altered strain curves of the individual LV deformation components from normal to pathological STE findings after viral infections allow to emphasize the diagnosis of acute myocarditis in TTE follow-up examinations (9). In such patient cohorts, a detailed STE which includes all components of LV deformation could avoid unnecessary cMRI examinations or serve to target pts for cMRI to confirm the diagnosis. A major argument for the causal correlation between structural findings in cMRI (predominantly LGE) and functional findings in STE (predominantly subepicardial CS) in pts with acute myocarditis is that the basal inferolateral LV segment was most affected by both methods.

### Clinical implications of residual myocardial involvement

After the acute stage, pts are at increased risk for cardiovascular events, e.g., malignant arrhythmias, compared with the general population. Even after mild and subclinical courses of acute myocarditis, subepicardial fibrosis can act as an arrhythmogenic substrate for malignant arrhythmias, which may result in an unfavorable prognosis in these pts (1, 3, 15). However, data on patient outcome depending on the severity of myocardial injury in pts with myocardial involvement due to viral infections are sparse (1–3, 28, 39, 43, 44). Despite their primary lack of functional relevance, small areas of fibrosis may cause persistent impairments of regional LV deformation, especially of rCS. Therefore, findings described in the present study have implications for future echocardiographic documentation, particularly due to standardized documentation of parasternal short-axis views (apical, mid, basal) so that all components of LV deformation can always be assessed. Persistent areas of fibrosis can also be observed after SARS-CoV-2 infection. In consequence of SARS-CoV-2 pandemic, it can be speculated that abnormalities of LV deformation might also be observed in a higher percentage during follow-up examinations in the future. Therefore, the present study sets the stage for follow-up studies to determine the prognostic value of persistent LV deformation abnormalities in pts after viral infections, including SARS-CoV-2.

### Limitations

The present study is mainly limited by the small number of pts. The most decisive reason for the limited number of pts was the purposeful selection of pts with positive cMRI findings and preserved LV systolic function. According to methodological requirements, pts with bundle branch blocks, arrhythmias, and poor acoustic windows were excluded. In addition, the acquisition of apical, mid, and basal parasternal short-axis views was not part of the daily standardized imaging protocol before the SARS-CoV-2 era, further decimating the number of complete data sets to assess all components of LV deformation. The labelling of the left ventricular segments primarily referred to the common standard of speckle tracking echocardiography using the 18-segment model (20, 21). Therefore, an 18-segment model has been chosen for both the speckle tracking analyses and the description of the CMR findings to allow a consistent comparison of the segments.

## Conclusion

The present study documents the feasibility of detecting regional deformation abnormalities by echocardiography in patients with acute myocarditis confirmed by cMRI. The most important echocardiographic finding in patients with acute myocarditis is an impairment of regional circumferential strain in the basal inferolateral LV segment, whereas assessment of global strain values does not appear to be of critical value.

## Data Availability

The original contributions presented in the study are included in the article/Supplementary Material, further inquiries can be directed to the corresponding author.

## References

[1] Ammirati E Cipriani M Moro C Raineri C Pini D Sormani P Clinical presentation and outcome in a contemporary cohort of patients with acute myocarditis: multicenter lombardy registry. Circulation. (2018) 138:1088–99. 10.1161/CIRCULATIONAHA.118.035319 29764898

[2] Trachtenberg BH Hare JM. Inflammatory cardiomyopathic syndromes. Circ Res. (2017) 121:803–18. 10.1161/CIRCRESAHA.117.310221 28912184

[3] Kragholm KH Lindgren FL Zaremba T Freeman P Andersen NH Riahi S Mortality and ventricular arrhythmia after acute myocarditis: a nationwide registry-based follow-up study. Open Heart. (2021) 8:e001806. 10.1136/openhrt-2021-001806 34675133 PMC8532546

[4] Ferreira VM Schulz-Menger J Holmvang G Kramer CM Carbone I Sechtem U Cardiovascular magnetic resonance in nonischemic myocardial inflammation. J Am Coll Cardiol. (2018) 72:3158–76. 10.1016/j.jacc.2018.09.072 30545455

[5] Friedrich MG Sechtem U Schulz-Menger J Holmvang G Alakija P Cooper LT Cardiovascular magnetic resonance in myocarditis: a JACC white paper. J Am Coll Cardiol. (2009) 53:1475–87. 10.1016/j.jacc.2009.02.007 19389557 PMC2743893

[6] Kotanidis CP Bazmpani M-A Haidich A-B Karvounis C Antoniades C Karamitsos TD. Diagnostic accuracy of cardiovascular magnetic resonance in acute myocarditis. JACC Cardiovasc Imaging. (2018) 11:1583–90. 10.1016/j.jcmg.2017.12.008 29454761

[7] Caforio ALP Pankuweit S Arbustini E Basso C Gimeno-Blanes J Felix SB Current state of knowledge on aetiology, diagnosis, management, and therapy of myocarditis: a position statement of the European society of cardiology working group on myocardial and pericardial diseases. Eur Heart J. (2013) 34:2636–48. 10.1093/eurheartj/eht210 23824828

[8] Kasner M Aleksandrov A Escher F Al-Saadi N Makowski M Spillmann F Multimodality imaging approach in the diagnosis of chronic myocarditis with preserved left ventricular ejection fraction (MCpEF): the role of 2D speckle-tracking echocardiography. Int J Cardiol. (2017) 243:374–8. 10.1016/j.ijcard.2017.05.038 28536004

[9] Döbel T Stöbe S Marshall RP Hepp P Fikenzer S Fikenzer K Possible new options and benefits to detect myocarditis, right ventricular remodeling and coronary anomalies by echocardiography in systematic preparticipation screening of athletes. Int J Cardiovasc Imaging. (2020) 36:1855–85. 10.1007/s10554-020-01899-1 32462448 PMC7497512

[10] Khoo NS Smallhorn JF Atallah J Kaneko S Mackie AS Paterson I. Altered left ventricular tissue velocities, deformation and twist in children and young adults with acute myocarditis and normal ejection fraction. J Am Soc Echocardiogr. (2012) 25:294–303. 10.1016/j.echo.2011.10.010 22101088

[11] Stöbe S Hagendorff A Gutberlet M Tayal B. Myocardial work: a modern tool to detect possible compensation mechanism of deformation in acute myocarditis with preserved left ventricular function. J Cardiovasc Echography. (2020) 30:206. 10.4103/jcecho.jcecho_48_20 PMC8021083 33828942

[12] Stöbe S Tayal B Tünnemann-Tarr A Hagendorff A. Dynamics in myocardial deformation as an indirect marker of myocardial involvement in acute myocarditis due to HIV infection: a case report. Eur Heart J Case Rep. (2021) 5:ytaa511. 10.1093/ehjcr/ytaa511 33598608 PMC7873785

[13] Tünnemann-Tarr A Stöbe S Laufs U Hagendorff A Tayal B. Speckle tracking echocardiography in a patient with viral myocarditis and acute myocardial infarction. J Cardiol Cases. (2020) 22:184–91. 10.1016/j.jccase.2020.06.011 33014202 PMC7520523

[14] Afonso L Hari P Pidlaoan V Kondur A Jacob S Khetarpal V. Acute myocarditis: can novel echocardiographic techniques assist with diagnosis? Eur Heart J Cardiovasc Imaging. (2010) 11:E5. 10.1093/ejechocard/jep183 19939815

[15] Te ALD Wu T-C Lin Y-J Chen Y-Y Chung F-P Chang S-L Increased risk of ventricular tachycardia and cardiovascular death in patients with myocarditis during the long-term follow-up: a national representative cohort from the national health insurance research database. Medicine. (2017) 96:e6633. 10.1097/MD.0000000000006633 28471960 PMC5419906

[16] Stöbe S Richter S Seige M Stehr S Laufs U Hagendorff A. Echocardiographic characteristics of patients with SARS-CoV-2 infection. Clin Res Cardiol. (2020) 109:1549–66. 10.1007/s00392-020-01727-5 32803387 PMC7428201

[17] Hofrichter P Hagendorff A Laufs U Fikenzer S Hepp P Marshall RP Analysis of left ventricular rotational deformation by 2D speckle tracking echocardiography: a feasibility study in athletes. Int J Cardiovasc Imaging. (2021) 37:2369–86. 10.1007/s10554-021-02213-3 33738612 PMC8302535

[18] Lang RM Badano LP Mor-Avi V Afilalo J Armstrong A Ernande L Recommendations for cardiac chamber quantification by echocardiography in adults: an update from the American society of echocardiography and the European association of cardiovascular imaging. Eur Heart J Cardiovasc Imaging. (2015) 16:233–71. 10.1093/ehjci/jev014 25712077

[19] Quiñones MA Otto CM Stoddard M Waggoner A Zoghbi WA. Recommendations for quantification of Doppler echocardiography: a report from the Doppler quantification task force of the nomenclature and standards committee of the American society of echocardiography. J Am Soc Echocardiogr. (2002) 15:167–84. 10.1067/mje.2002.120202 11836492

[20] Leitman M Lysyansky P Sidenko S Shir V Peleg E Binenbaum M Two-dimensional strain–a novel software for real-time quantitative echocardiographic assessment of myocardial function. J Am Soc Echocardiogr. (2004) 17:1021–9. 10.1016/j.echo.2004.06.019 15452466

[21] Voigt J-U Pedrizzetti G Lysyansky P Marwick TH Houle H Baumann R Definitions for a common standard for 2D speckle tracking echocardiography: consensus document of the EACVI/ASE/industry task force to standardize deformation imaging. Eur Heart J Cardiovasc Imaging. (2015) 16:1–11. 10.1093/ehjci/jeu184 25525063

[22] Leitman M Lysiansky M Lysyansky P Friedman Z Tyomkin V Fuchs T Circumferential and longitudinal strain in 3 myocardial layers in normal subjects and in patients with regional left ventricular dysfunction. J Am Soc Echocardiogr. (2010) 23:64–70. 10.1016/j.echo.2009.10.004 20122496

[23] Shi J Pan C Kong D Cheng L Shu X. Left ventricular longitudinal and circumferential layer-specific myocardial strains and their determinants in healthy subjects. Echocardiography. (2016) 33:510–8. 10.1111/echo.13132 26661049

[24] Stöhr EJ Shave RE Baggish AL Weiner RB. Left ventricular twist mechanics in the context of normal physiology and cardiovascular disease: a review of studies using speckle tracking echocardiography. Am J Physiol Heart Circ Physiol. (2016) 311:H633–44. 10.1152/ajpheart.00104.2016 27402663

[25] Yingchoncharoen T Agarwal S Popović ZB Marwick TH. Normal ranges of left ventricular strain: a meta-analysis. J Am Soc Echocardiogr. (2013) 26:185–91. 10.1016/j.echo.2012.10.008 23218891

[26] Marwick TH Leano RL Brown J Sun J-P Hoffmann R Lysyansky P Myocardial strain measurement with 2-dimensional speckle-tracking echocardiography. JACC: Cardiovasc Imaging. (2009) 2:80–4. 10.1016/j.jcmg.2007.12.007 19356538

[27] Messroghli DR Moon JC Ferreira VM Grosse-Wortmann L He T Kellman P Clinical recommendations for cardiovascular magnetic resonance mapping of T1, T2, T2* and extracellular volume: a consensus statement by the society for cardiovascular magnetic resonance (SCMR) endorsed by the European association for cardiovascular imaging (EACVI). J Cardiovasc Magn Reson. (2017) 19:75. 10.1186/s12968-017-0389-8 28992817 PMC5633041

[28] Ammirati E Frigerio M Adler ED Basso C Birnie DH Brambatti M Management of acute myocarditis and chronic inflammatory cardiomyopathy: an expert consensus document. Circ Heart Fail. (2020) 13:e007405. 10.1161/CIRCHEARTFAILURE.120.007405 33176455 PMC7673642

[29] Mahrholdt H Wagner A Judd RM Sechtem U Kim RJ. Delayed enhancement cardiovascular magnetic resonance assessment of non-ischaemic cardiomyopathies. Eur Heart J. (2005) 26:1461–74. 10.1093/eurheartj/ehi258 15831557

[30] Tschöpe C Cooper LT Torre-Amione G Van Linthout S. Management of myocarditis-related cardiomyopathy in adults. Circ Res. (2019) 124:1568–83. 10.1161/CIRCRESAHA.118.313578 31120823

[31] Tschöpe C Ammirati E Bozkurt B Caforio ALP Cooper LT Felix SB Myocarditis and inflammatory cardiomyopathy: current evidence and future directions. Nat Rev Cardiol. (2021) 18:169–93. 10.1038/s41569-020-00435-x 33046850 PMC7548534

[32] Tsugu T Postolache A Dulgheru R Sugimoto T Tridetti J Nguyen Trung M-L Echocardiographic reference ranges for normal left ventricular layer-specific strain: results from the EACVI NORRE study. Eur Heart J Cardiovasc Imaging. (2020) 21:896–905. 10.1093/ehjci/jeaa050 32259844

[33] Helle-Valle T Crosby J Edvardsen T Lyseggen E Amundsen BH Smith H-J New noninvasive method for assessment of left ventricular rotation: speckle tracking echocardiography. Circulation. (2005) 112:3149–56. 10.1161/CIRCULATIONAHA.104.531558 16286606

[34] Notomi Y Shiota T Popovic ZB Weaver JA Oryszak SJ Greenberg NL Measurement of ventricular torsion by two-dimensional ultrasound speckle tracking imaging. J Am Coll Cardiol. (2005) 45:2034–41. 10.1016/j.jacc.2005.02.082 15963406

[35] Kim H-K Sohn D-W Lee S-E Choi S-Y Park J-S Kim Y-J Assessment of left ventricular rotation and torsion with two-dimensional speckle tracking echocardiography. J Am Soc Echocardiogr. (2007) 20:45–53. 10.1016/j.echo.2006.07.007 17218201

[36] Goffinet C Chenot F Robert A Pouleur A-C De Waroux J-BLP Vancrayenest D Assessment of subendocardial vs. subepicardial left ventricular rotation and twist using two-dimensional speckle tracking echocardiography: comparison with tagged cardiac magnetic resonance. Eur Heart J. (2009) 30:608–17. 10.1093/eurheartj/ehn511 19019994

[37] Spriestersbach H Oh-Icí D Schmitt B Berger F Schmitz L. The influence of the region of interest width on two-dimensional speckle tracking-based measurements of strain and strain rate. Echocardiography. (2015) 32:89–95. 10.1111/echo.12589 24665977

[38] Mirea O Pagourelias ED Duchenne J Bogaert J Thomas JD Badano LP Intervendor differences in the accuracy of detecting regional functional abnormalities. JACC Cardiovasc Imaging. (2018) 11:25–34. 10.1016/j.jcmg.2017.02.014 28528162

[39] Nappi F Avtaar Singh SS. SARS-CoV-2-induced myocarditis: a state-of-the-art review. Viruses. (2023) 15:916. 10.3390/v15040916 37112896 PMC10145666

[40] Ammirati E Lupi L Palazzini M Hendren NS Grodin JL Cannistraci CV Prevalence, characteristics, and outcomes of COVID-19–associated acute myocarditis. Circulation. (2022) 145:1123–39. 10.1161/CIRCULATIONAHA.121.056817 35404682 PMC8989611

[41] Kornowski R Witberg G. Acute myocarditis caused by COVID-19 disease and following COVID-19 vaccination. Open Heart. (2022) 9:e001957. 10.1136/openhrt-2021-001957 35264415 PMC8914394

[42] Voleti N Reddy SP Ssentongo P. Myocarditis in SARS-CoV-2 infection vs. COVID-19 vaccination: a systematic review and meta-analysis. Front Cardiovasc Med. (2022) 9:951314. 10.3389/fcvm.2022.951314 36105535 PMC9467278

[43] Puntmann VO Carerj ML Wieters I Fahim M Arendt C Hoffmann J Outcomes of cardiovascular magnetic resonance imaging in patients recently recovered from coronavirus disease 2019 (COVID-19). JAMA Cardiol. (2020) 5:1265. 10.1001/jamacardio.2020.3557 32730619 PMC7385689

[44] Shchendrygina A Nagel E Puntmann VO Valbuena-Lopez S. COVID-19 myocarditis and prospective heart failure burden. Expert Rev Cardiovasc Ther. (2021) 19:5–14. 10.1080/14779072.2021.1844005 33119418

