# Supplementary material for: Comparison of left ventricular deformation abnormalities by echocardiography with cardiac magnetic resonance imaging in patients with acute myocarditis and preserved left ventricular ejection fraction
Source: Front Cardiovasc Med. 2024 Jan 9;10:1322145. doi: 10.3389/fcvm.2023.1322145 (PMC10803407; doi:10.3389/fcvm.2023.1322145)
Supplement: Supplementary file 1 [file Datasheet1.pdf]

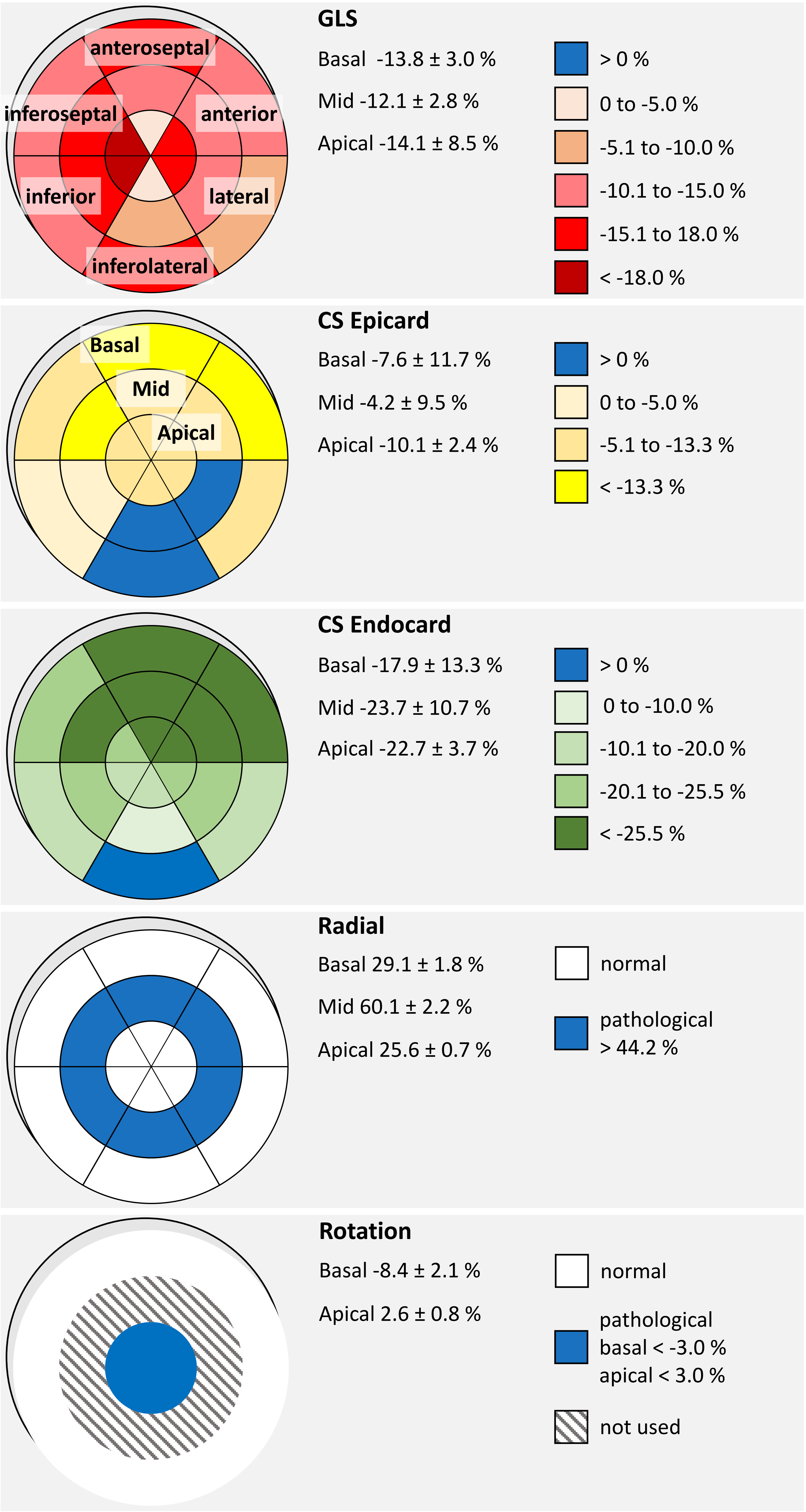
anteroseptal
inferoseptal
anterior
inferior
lateral
inferolateral
GLS
Basal -13.8 ± 3.0 %
Mid -12.1 ± 2.8 %
Apical -14.1 ± 8.5 %
> 0 %
0 to -5.0 %
-5.1 to -10.0 %
-10.1 to -15.0 %
-15.1 to 18.0 %
< -18.0 %
Basal
Mid
Apical
CS Epicard
Basal -7.6 ± 11.7 %
Mid -4.2 ± 9.5 %
Apical -10.1 ± 2.4 %
> 0 %
0 to -5.0 %
-5.1 to -13.3 %
< -13.3 %
CS Endocard
Basal -17.9 ± 13.3 %
Mid -23.7 ± 10.7 %
Apical -22.7 ± 3.7 %
> 0 %
0 to -10.0 %
-10.1 to -20.0 %
-20.1 to -25.5 %
< -25.5 %
Radial
Basal 29.1 ± 1.8 %
Mid 60.1 ± 2.2 %
Apical 25.6 ± 0.7 %
normal
pathological
> 44.2 %
Rotation
Basal -8.4 ± 2.1 %
Apical 2.6 ± 0.8 %
normal
pathological
basal < -3.0 %
apical < 3.0 %
not used

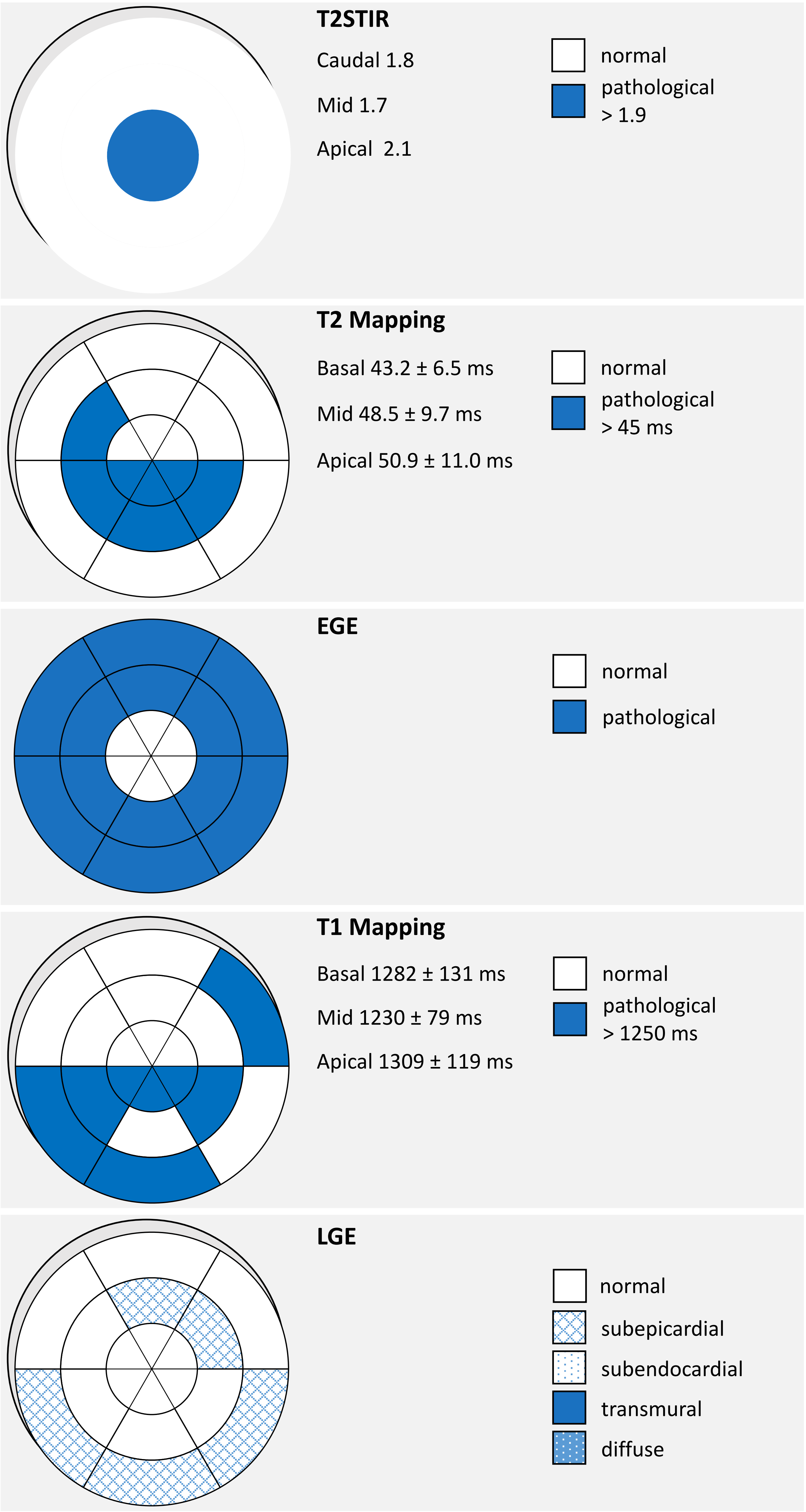

T2STIR
Caudal 1.8
Mid 1.7
Apical 2.1
normal
pathological
> 1.9
T2 Mapping
Basal 43.2 ± 6.5 ms
Mid 48.5 ± 9.7 ms
Apical 50.9 ± 11.0 ms
normal
pathological
> 45 ms
EGE
normal
pathological
T1 Mapping
Basal 1282 ± 131 ms
Mid 1230 ± 79 ms
Apical 1309 ± 119 ms
normal
pathological
> 1250 ms
LGE
normal
subepicardial
subendocardial
transmural
diffuse

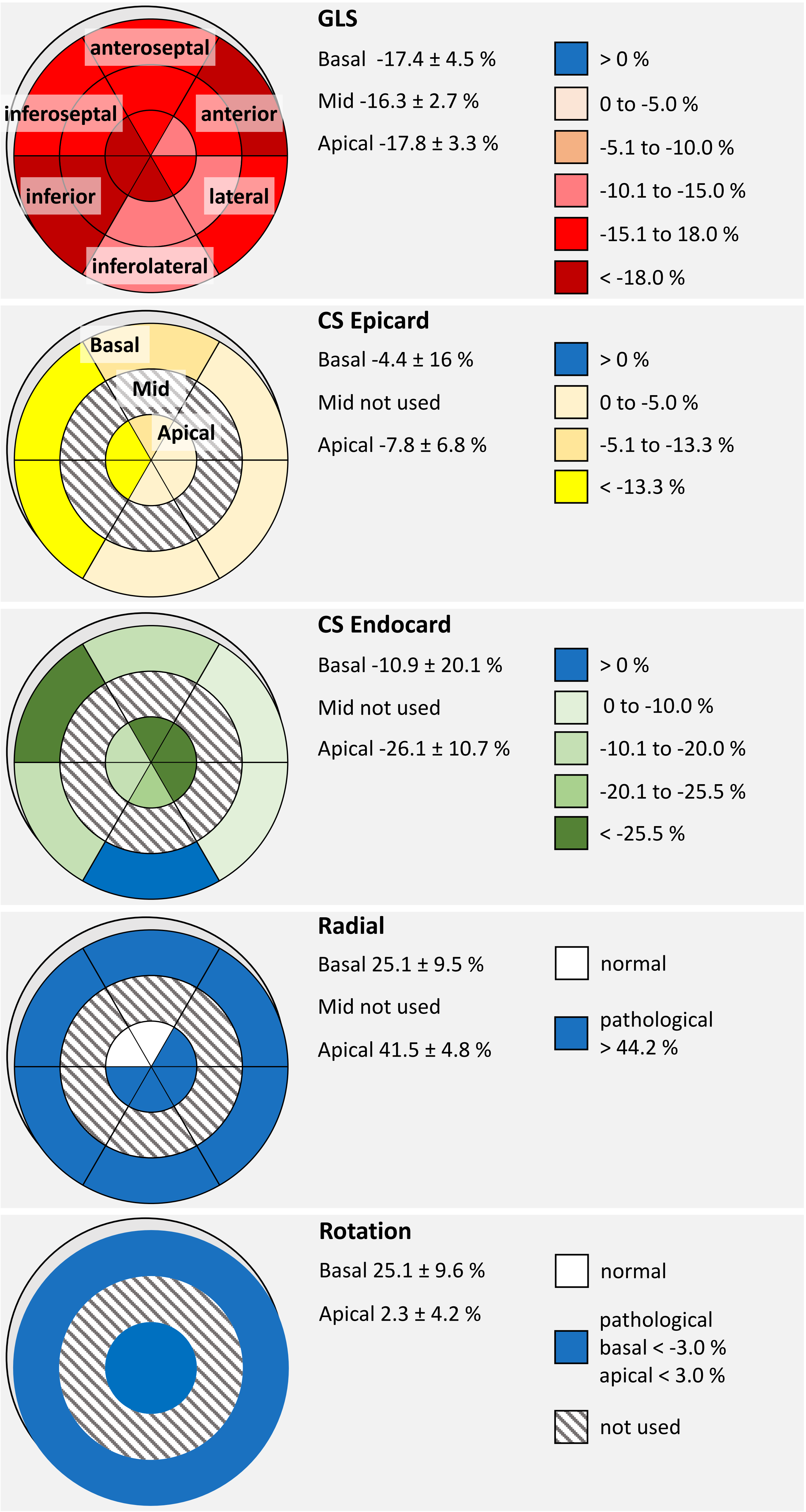

anteroseptal
inferoseptal
anterior
inferior
lateral
inferolateral
GLS
Basal -17.4 ± 4.5 %
Mid -16.3 ± 2.7 %
Apical -17.8 ± 3.3 %
> 0 %
0 to -5.0 %
-5.1 to -10.0 %
-10.1 to -15.0 %
-15.1 to 18.0 %
< -18.0 %
Basal
Mid
Apical
CS Epicard
Basal -4.4 ± 16 %
Mid not used
Apical -7.8 ± 6.8 %
> 0 %
0 to -5.0 %
-5.1 to -13.3 %
< -13.3 %
CS Endocard
Basal -10.9 ± 20.1 %
Mid not used
Apical -26.1 ± 10.7 %
> 0 %
0 to -10.0 %
-10.1 to -20.0 %
-20.1 to -25.5 %
< -25.5 %
Radial
Basal 25.1 ± 9.5 %
Mid not used
Apical 41.5 ± 4.8 %
normal
pathological
> 44.2 %
Rotation
Basal 25.1 ± 9.6 %
Apical 2.3 ± 4.2 %
normal
pathological
basal < -3.0 %
apical < 3.0 %
not used

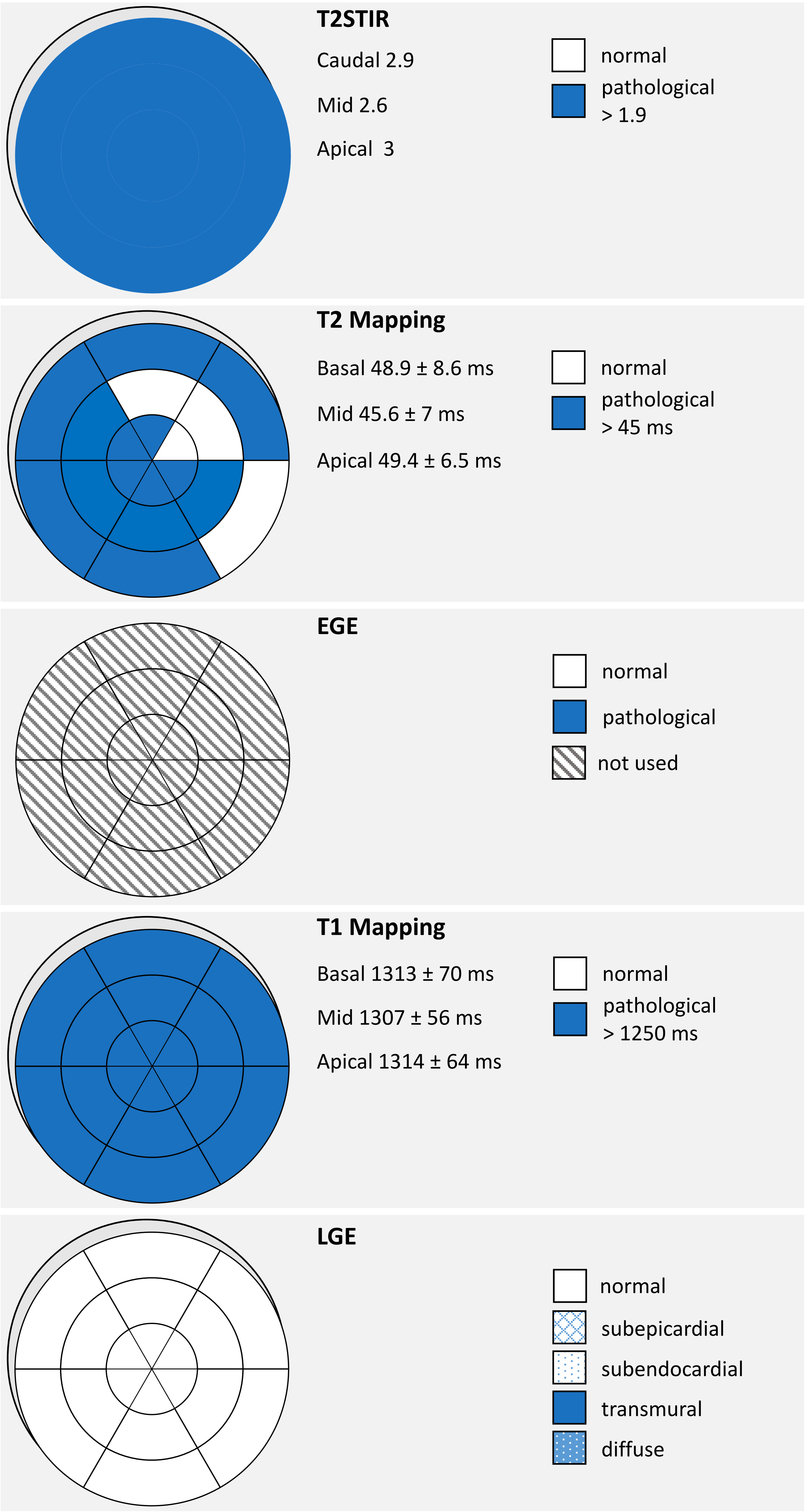
T2STIR
Caudal 2.9
Mid 2.6
Apical 3
normal
pathological
> 1.9
T2 Mapping
Basal 48.9 ± 8.6 ms
Mid 45.6 ± 7 ms
Apical 49.4 ± 6.5 ms
normal
pathological
> 45 ms
EGE
normal
pathological
not used
T1 Mapping
Basal 1313 ± 70 ms
Mid 1307 ± 56 ms
Apical 1314 ± 64 ms
normal
pathological
> 1250 ms
LGE
normal
subepicardial
subendocardial
transmural
diffuse

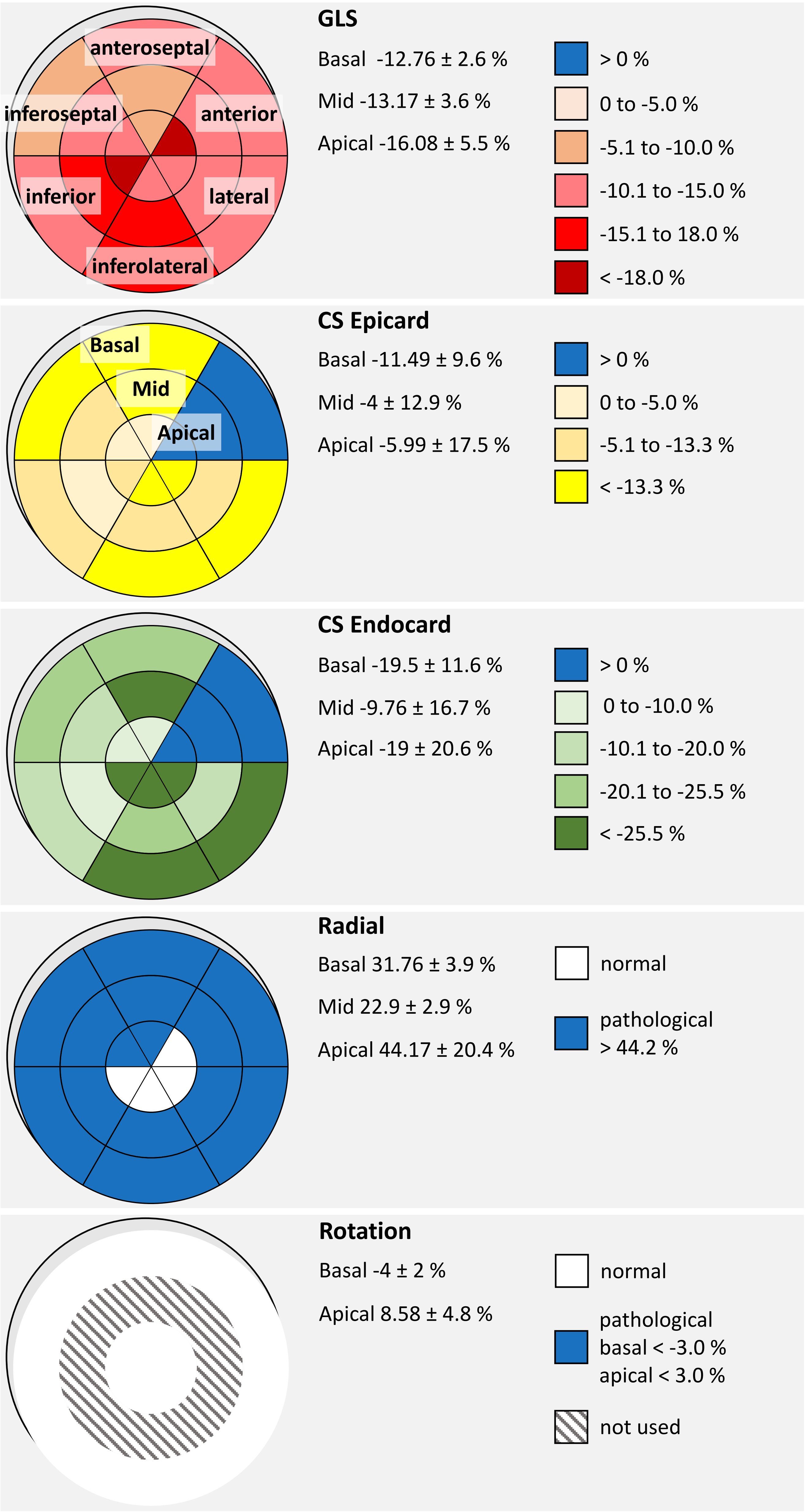
anteroseptal
inferoseptal
anterior
inferior
lateral
inferolateral
GLS
Basal -12.76 ± 2.6 %
Mid -13.17 ± 3.6 %
Apical -16.08 ± 5.5 %
> 0 %
0 to -5.0 %
-5.1 to -10.0 %
-10.1 to -15.0 %
-15.1 to 18.0 %
< -18.0 %
Basal
Mid
Apical
CS Epicard
Basal -11.49 ± 9.6 %
Mid -4 ± 12.9 %
Apical -5.99 ± 17.5 %
> 0 %
0 to -5.0 %
-5.1 to -13.3 %
< -13.3 %
CS Endocard
Basal -19.5 ± 11.6 %
Mid -9.76 ± 16.7 %
Apical -19 ± 20.6 %
> 0 %
0 to -10.0 %
-10.1 to -20.0 %
-20.1 to -25.5 %
< -25.5 %
Radial
Basal 31.76 ± 3.9 %
Mid 22.9 ± 2.9 %
Apical 44.17 ± 20.4 %
normal
pathological
> 44.2 %
Rotation
Basal -4 ± 2 %
Apical 8.58 ± 4.8 %
normal
pathological
basal < -3.0 %
apical < 3.0 %
not used

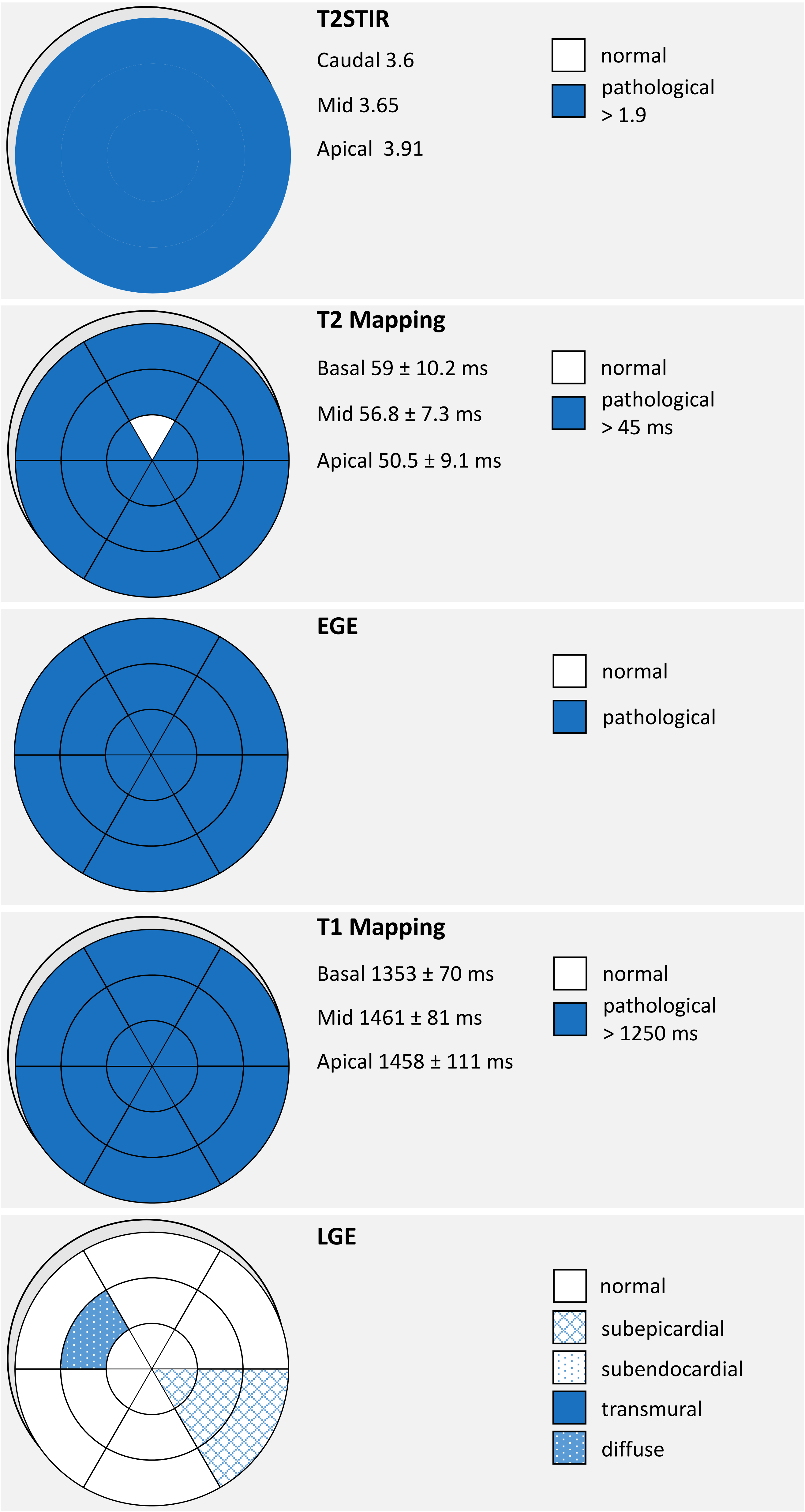

T2STIR
Caudal 3.6
Mid 3.65
Apical 3.91
normal
pathological
> 1.9
T2 Mapping
Basal 59 ± 10.2 ms
Mid 56.8 ± 7.3 ms
Apical 50.5 ± 9.1 ms
normal
pathological
> 45 ms
EGE
normal
pathological
T1 Mapping
Basal 1353 ± 70 ms
Mid 1461 ± 81 ms
Apical 1458 ± 111 ms
normal
pathological
> 1250 ms
LGE
normal
subepicardial
subendocardial
transmural
diffuse

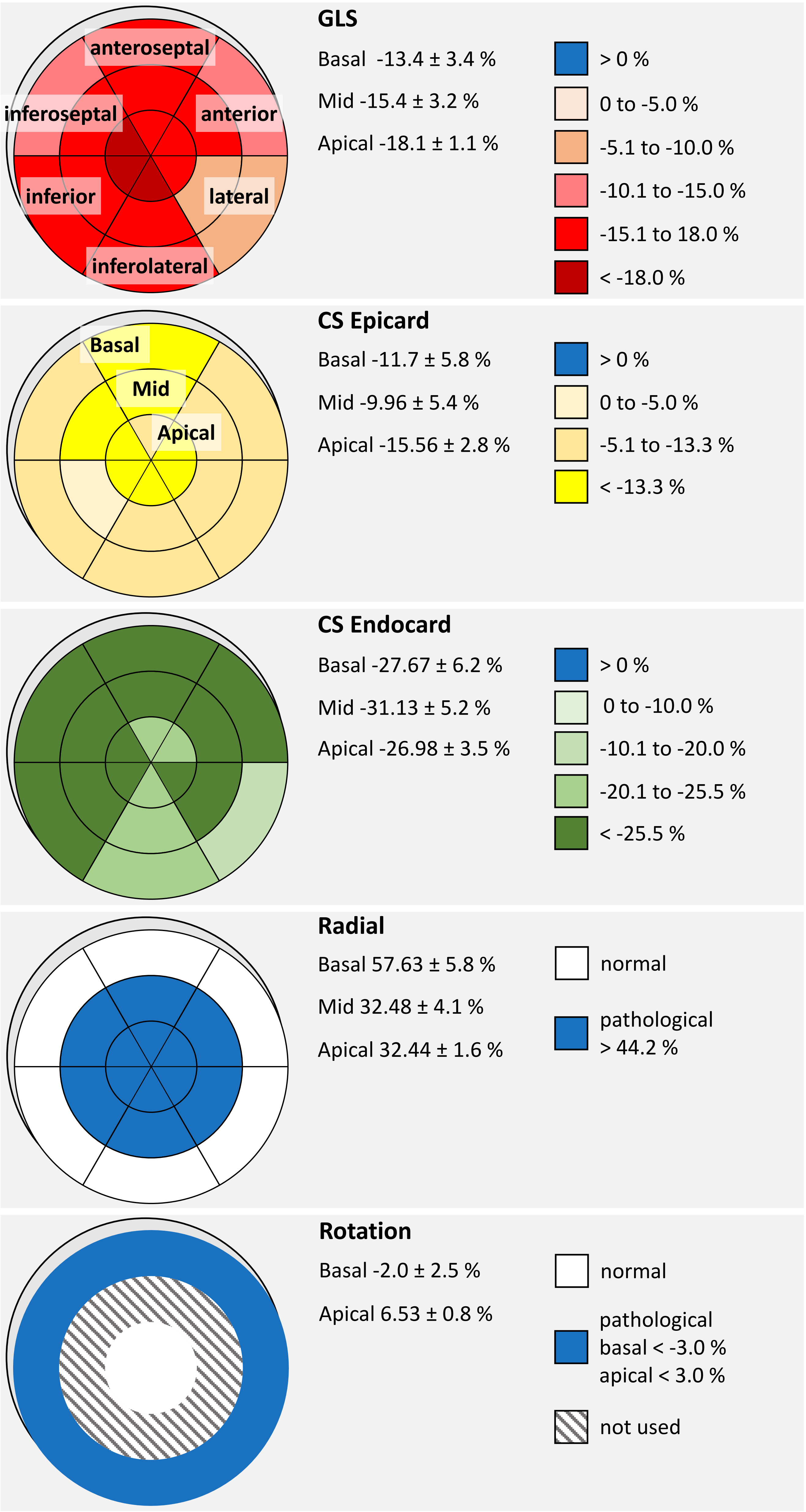
anteroseptal
inferoseptal
anterior
inferior
lateral
inferolateral
GLS
Basal -13.4 ± 3.4 %
Mid -15.4 ± 3.2 %
Apical -18.1 ± 1.1 %
> 0 %
0 to -5.0 %
-5.1 to -10.0 %
-10.1 to -15.0 %
-15.1 to 18.0 %
< -18.0 %
Basal
Mid
Apical
CS Epicard
Basal -11.7 ± 5.8 %
Mid -9.96 ± 5.4 %
Apical -15.56 ± 2.8 %
> 0 %
0 to -5.0 %
-5.1 to -13.3 %
< -13.3 %
CS Endocard
Basal -27.67 ± 6.2 %
Mid -31.13 ± 5.2 %
Apical -26.98 ± 3.5 %
> 0 %
0 to -10.0 %
-10.1 to -20.0 %
-20.1 to -25.5 %
< -25.5 %
Radial
Basal 57.63 ± 5.8 %
Mid 32.48 ± 4.1 %
Apical 32.44 ± 1.6 %
normal
pathological
> 44.2 %
Rotation
Basal -2.0 ± 2.5 %
Apical 6.53 ± 0.8 %
normal
pathological
basal < -3.0 %
apical < 3.0 %
not used

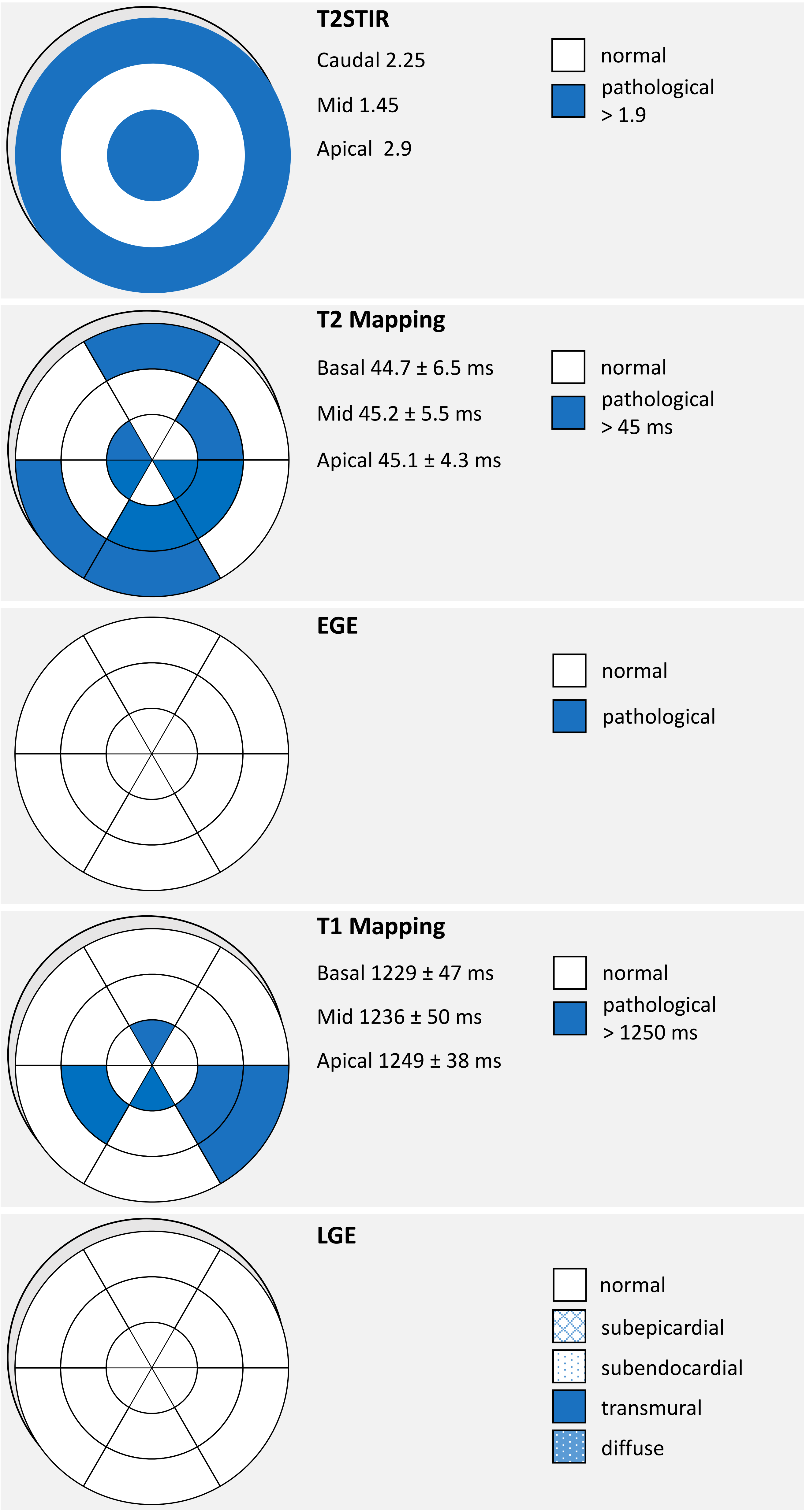

T2STIR
Caudal 2.25
Mid 1.45
Apical 2.9
normal
pathological
> 1.9
T2 Mapping
Basal 44.7 ± 6.5 ms
Mid 45.2 ± 5.5 ms
Apical 45.1 ± 4.3 ms
normal
pathological
> 45 ms
EGE
normal
pathological
T1 Mapping
Basal 1229 ± 47 ms
Mid 1236 ± 50 ms
Apical 1249 ± 38 ms
normal
pathological
> 1250 ms
LGE
normal
subepicardial
subendocardial
transmural
diffuse

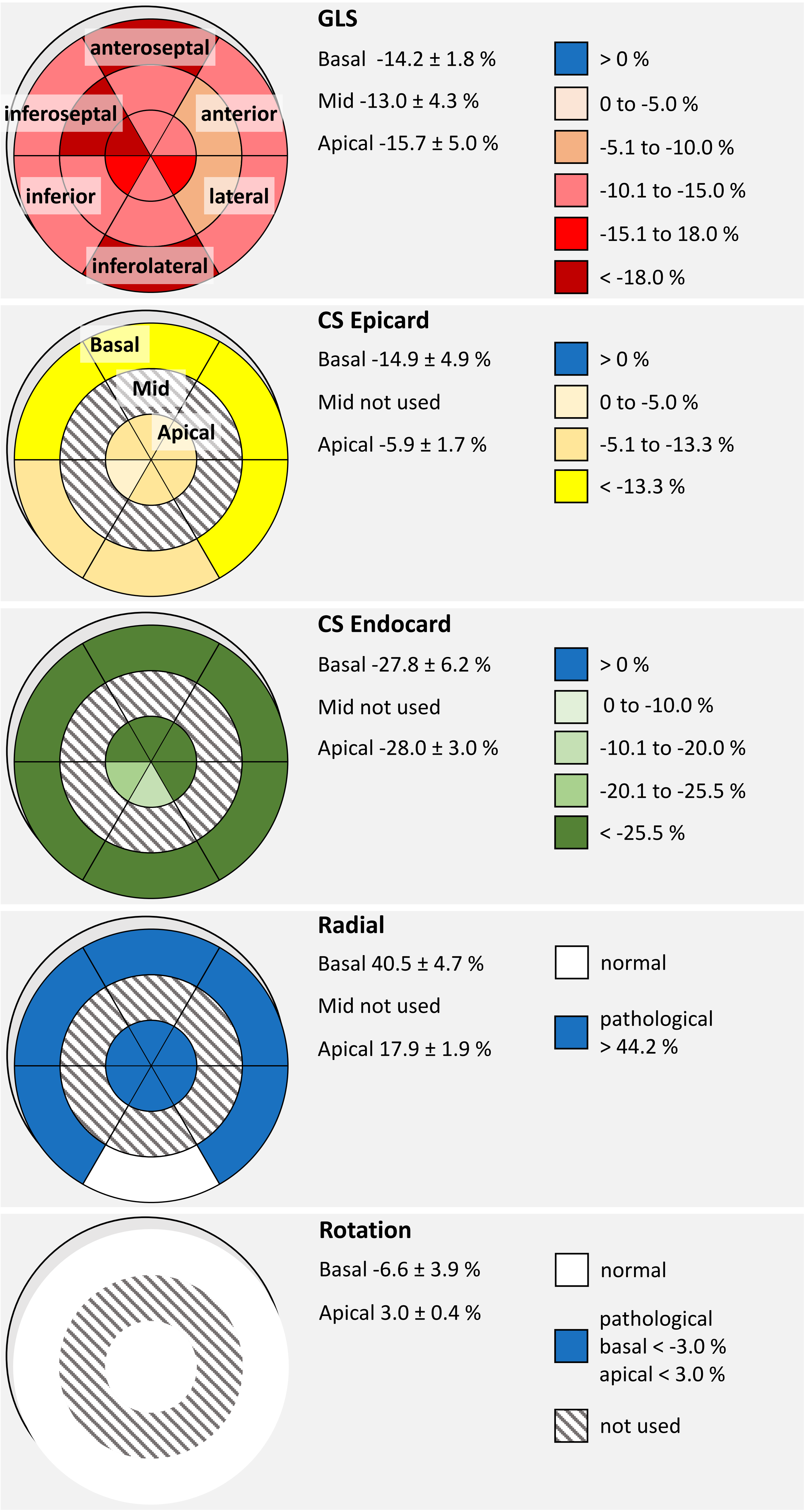

anteroseptal
inferoseptal
anterior
inferior
lateral
inferolateral
GLS
Basal -14.2 ± 1.8 %
Mid -13.0 ± 4.3 %
Apical -15.7 ± 5.0 %
> 0 %
0 to -5.0 %
-5.1 to -10.0 %
-10.1 to -15.0 %
-15.1 to 18.0 %
< -18.0 %
Basal
Mid
Apical
CS Epicard
Basal -14.9 ± 4.9 %
Mid not used
Apical -5.9 ± 1.7 %
> 0 %
0 to -5.0 %
-5.1 to -13.3 %
< -13.3 %
CS Endocard
Basal -27.8 ± 6.2 %
Mid not used
Apical -28.0 ± 3.0 %
> 0 %
0 to -10.0 %
-10.1 to -20.0 %
-20.1 to -25.5 %
< -25.5 %
Radial
Basal 40.5 ± 4.7 %
Mid not used
Apical 17.9 ± 1.9 %
normal
pathological
> 44.2 %
Rotation
Basal -6.6 ± 3.9 %
Apical 3.0 ± 0.4 %
normal
pathological
basal < -3.0 %
apical < 3.0 %
not used

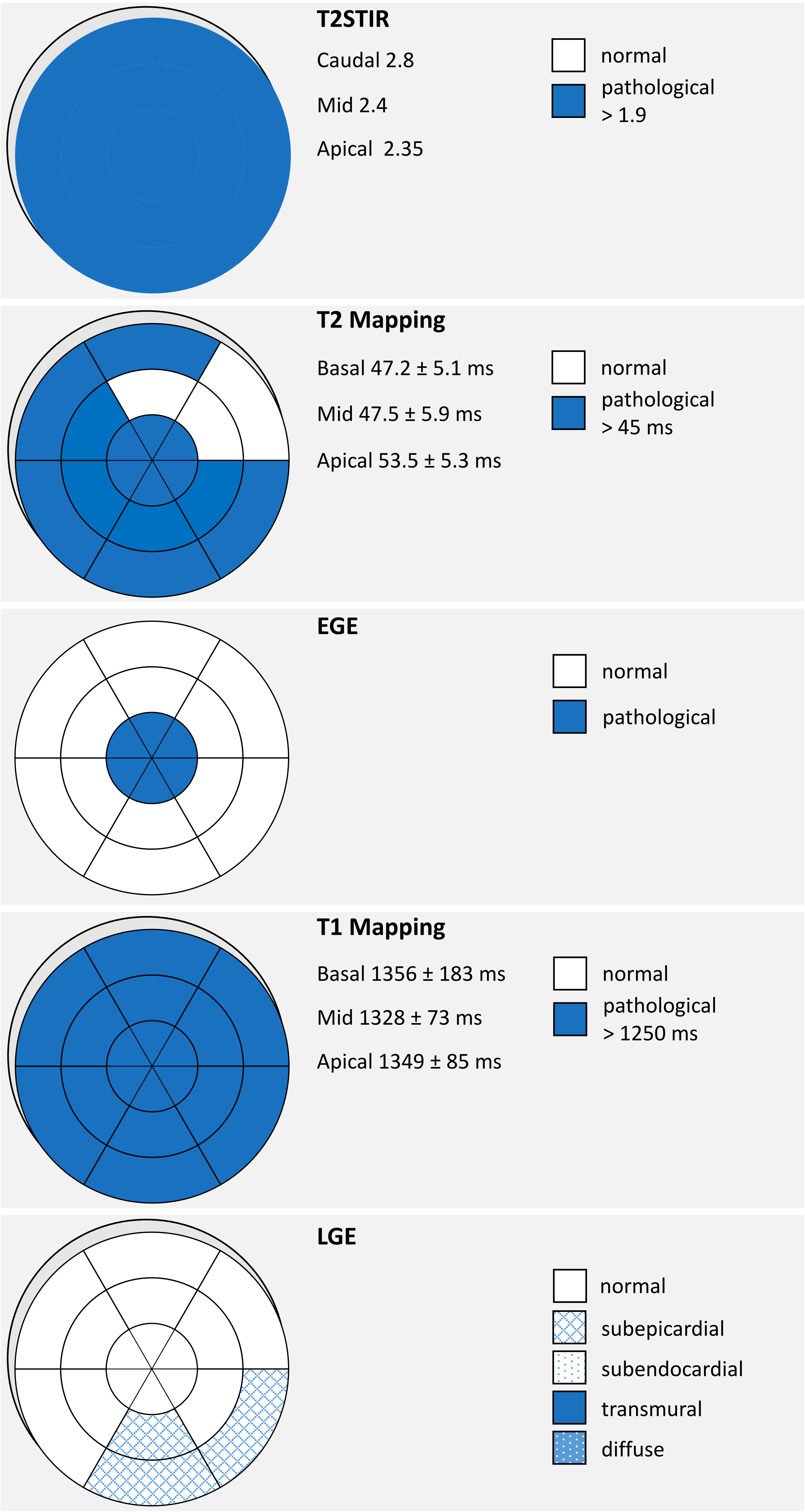
T2STIR
Caudal 2.8
Mid 2.4
Apical 2.35
normal
pathological
> 1.9
T2 Mapping
Basal 47.2 ± 5.1 ms
Mid 47.5 ± 5.9 ms
Apical 53.5 ± 5.3 ms
normal
pathological
> 45 ms
EGE
normal
pathological
T1 Mapping
Basal 1356 ± 183 ms
Mid 1328 ± 73 ms
Apical 1349 ± 85 ms
normal
pathological
> 1250 ms
LGE
normal
subepicardial
subendocardial
transmural
diffuse

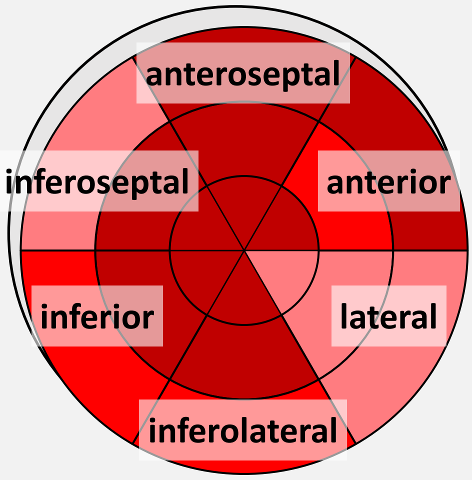

GLS

Basal -16.66 ± 2.6 %

Mid -18.83 ± 3.4 %

Apical -22.63 ± 6.1 %

- > 0 %
- 0 to -5.0 %
- -5.1 to -10.0 %
- -10.1 to -15.0 %
- -15.1 to 18.0 %
- < -18.0 %

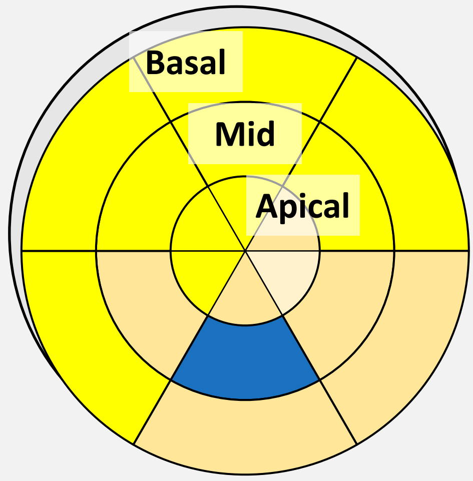

CS Epicard

Basal -16.78 ± 9.3 %

Mid -10.21 ± 24.2 %

Apical -14.28 ± 8.5 %

- > 0 %
- 0 to -5.0 %
- -5.1 to -13.3 %
- < -13.3 %

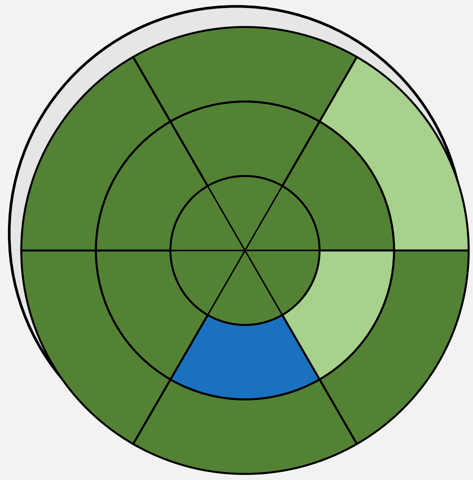

CS Endocard

Basal -34.43 ± 9.2 %

Mid -22.8 ± 35.1 %

Apical -39.5 ± 5.3 %

- > 0 %
- 0 to -10.0 %
- -10.1 to -20.0 %
- -20.1 to -25.5 %
- < -25.5 %

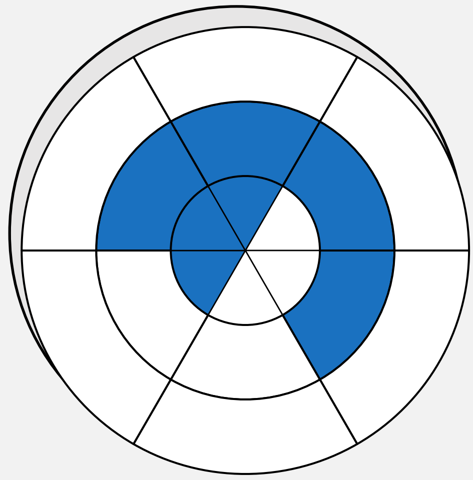

Radial

Basal 46.42 ± 1.5 %

Mid 42.81 ± 10.2 %

Apical 49.64 ± 22 %

- normal
- pathological > 44.2 %

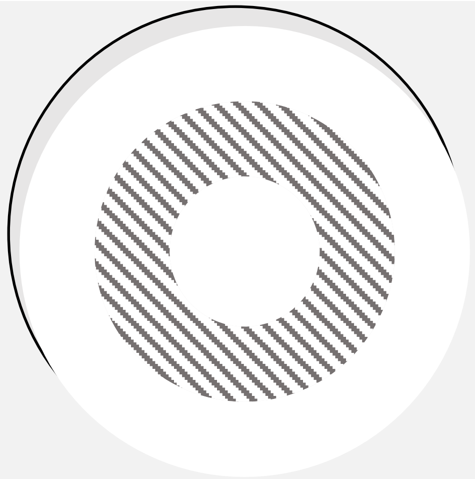

Rotation

Basal -7.56 ± 4.1 %

Apical 5.14 ± 3.2 %

- normal
- pathological basal < -3.0 % apical < 3.0 %
- not used

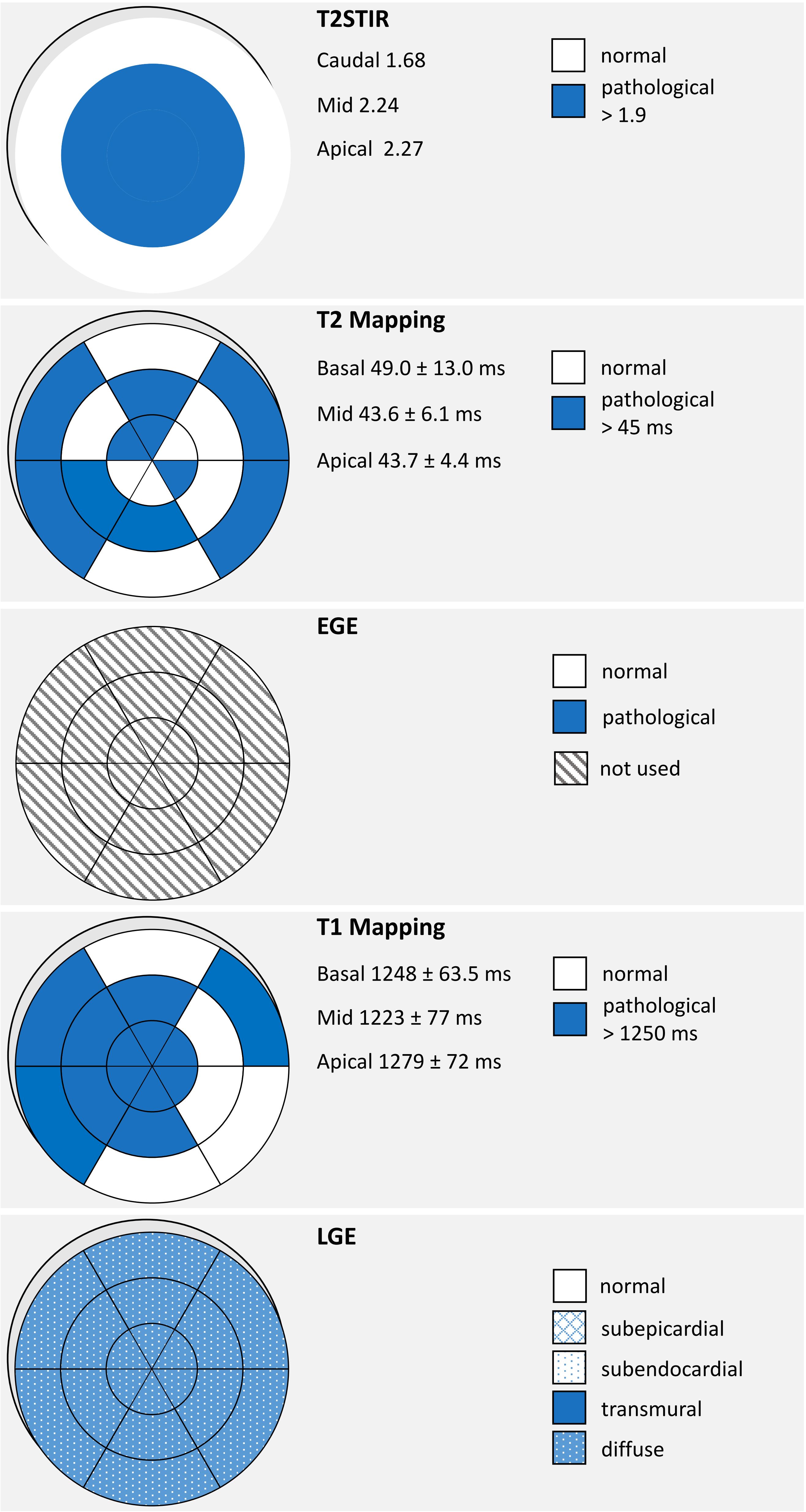
T2STIR
Caudal 1.68
Mid 2.24
Apical 2.27
normal
pathological > 1.9
T2 Mapping
Basal 49.0 ± 13.0 ms
Mid 43.6 ± 6.1 ms
Apical 43.7 ± 4.4 ms
normal
pathological > 45 ms
EGE
normal
pathological
not used
T1 Mapping
Basal 1248 ± 63.5 ms
Mid 1223 ± 77 ms
Apical 1279 ± 72 ms
normal
pathological > 1250 ms
LGE
normal
subepicardial
subendocardial
transmural
diffuse

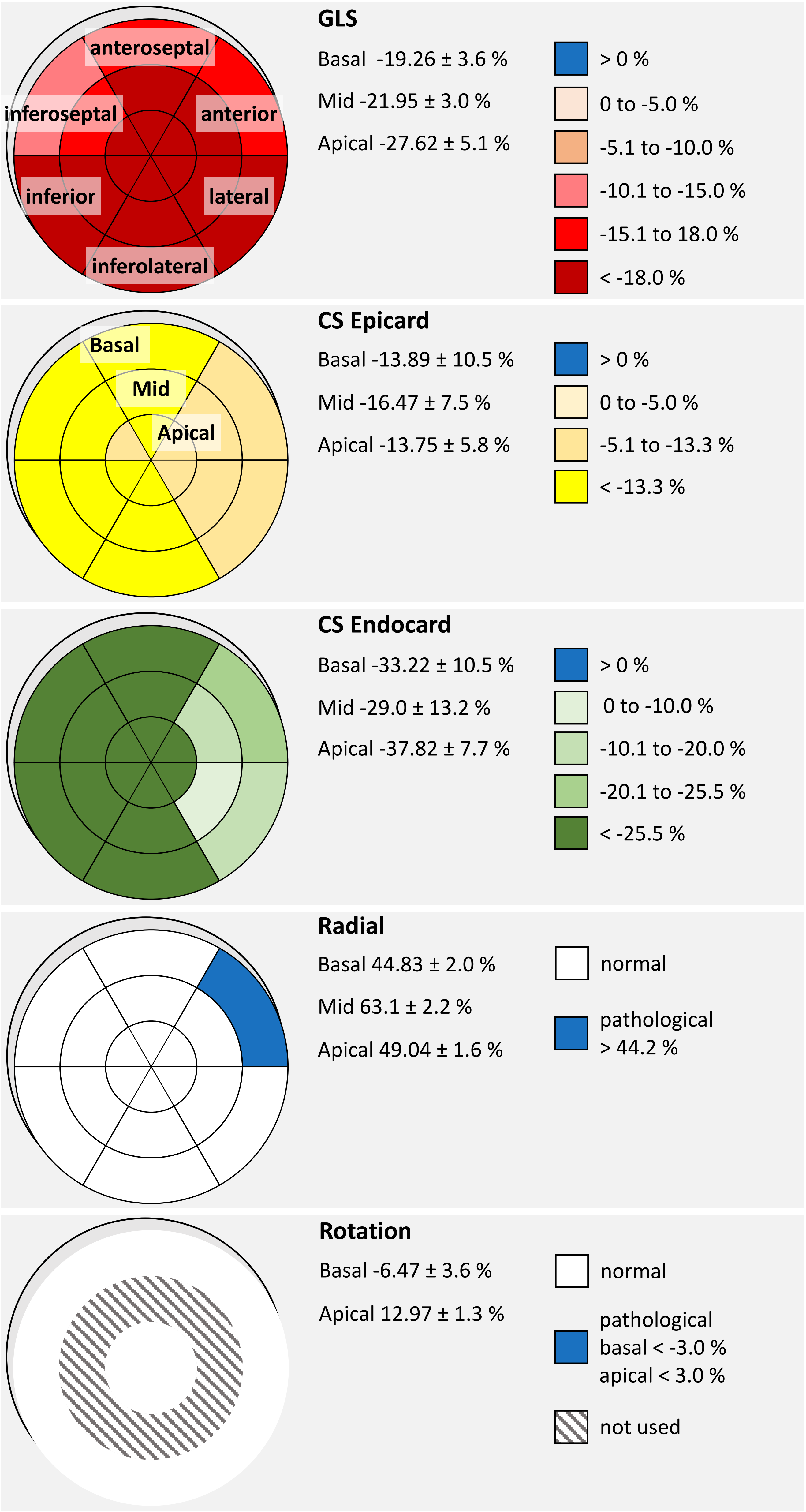
anteroseptal
inferoseptal
anterior
inferior
lateral
inferolateral
GLS
Basal -19.26 ± 3.6 %
Mid -21.95 ± 3.0 %
Apical -27.62 ± 5.1 %
> 0 %
0 to -5.0 %
-5.1 to -10.0 %
-10.1 to -15.0 %
-15.1 to 18.0 %
< -18.0 %
Basal
Mid
Apical
CS Epicard
Basal -13.89 ± 10.5 %
Mid -16.47 ± 7.5 %
Apical -13.75 ± 5.8 %
> 0 %
0 to -5.0 %
-5.1 to -13.3 %
< -13.3 %
CS Endocard
Basal -33.22 ± 10.5 %
Mid -29.0 ± 13.2 %
Apical -37.82 ± 7.7 %
> 0 %
0 to -10.0 %
-10.1 to -20.0 %
-20.1 to -25.5 %
< -25.5 %
Radial
Basal 44.83 ± 2.0 %
Mid 63.1 ± 2.2 %
Apical 49.04 ± 1.6 %
normal
pathological
> 44.2 %
Rotation
Basal -6.47 ± 3.6 %
Apical 12.97 ± 1.3 %
normal
pathological
basal < -3.0 %
apical < 3.0 %
not used

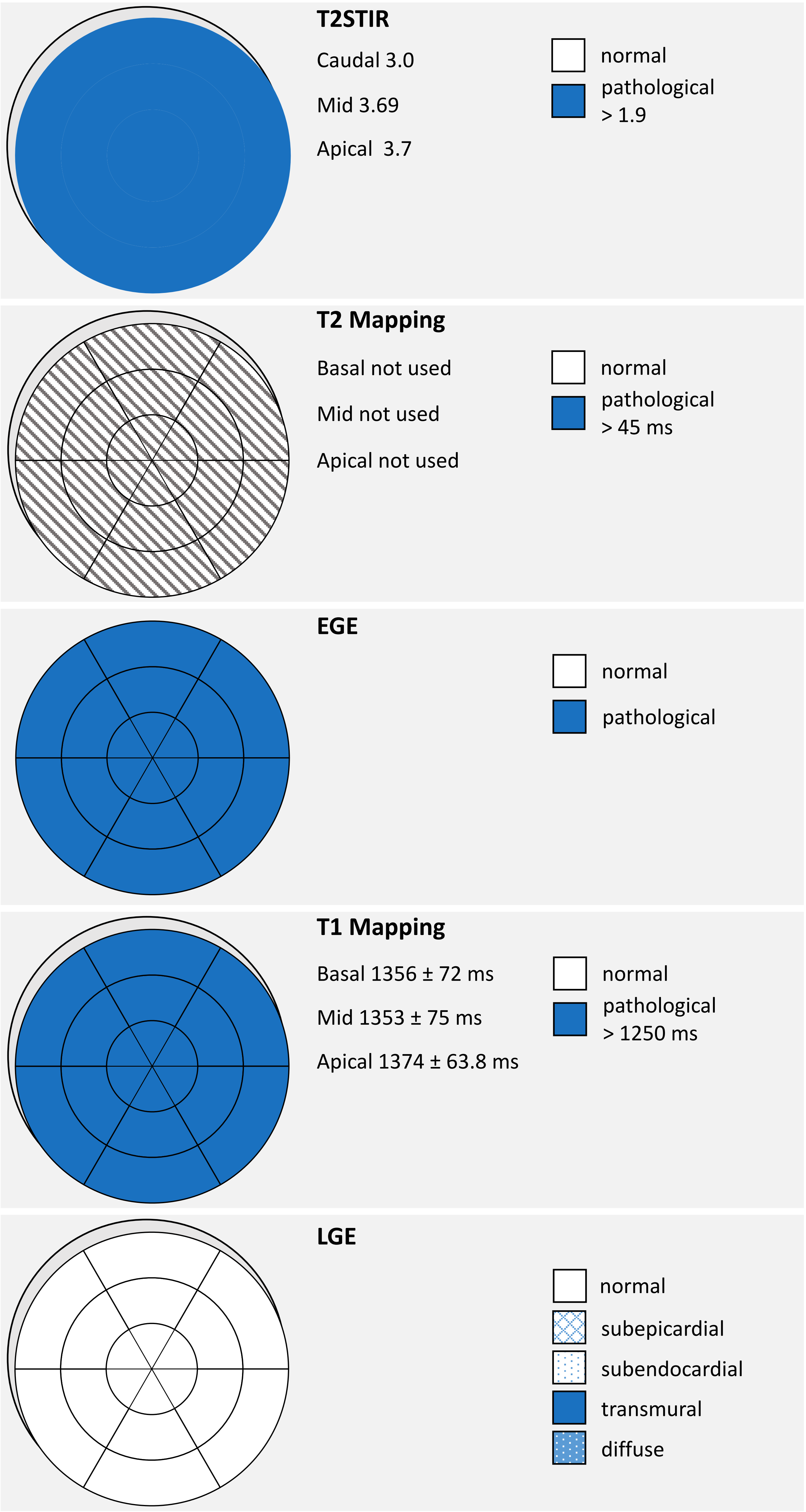
T2STIR
Caudal 3.0
Mid 3.69
Apical 3.7
normal
pathological > 1.9
T2 Mapping
Basal not used
Mid not used
Apical not used
normal
pathological > 45 ms
EGE
normal
pathological
T1 Mapping
Basal 1356 ± 72 ms
Mid 1353 ± 75 ms
Apical 1374 ± 63.8 ms
normal
pathological > 1250 ms
LGE
normal
subepicardial
subendocardial
transmural
diffuse

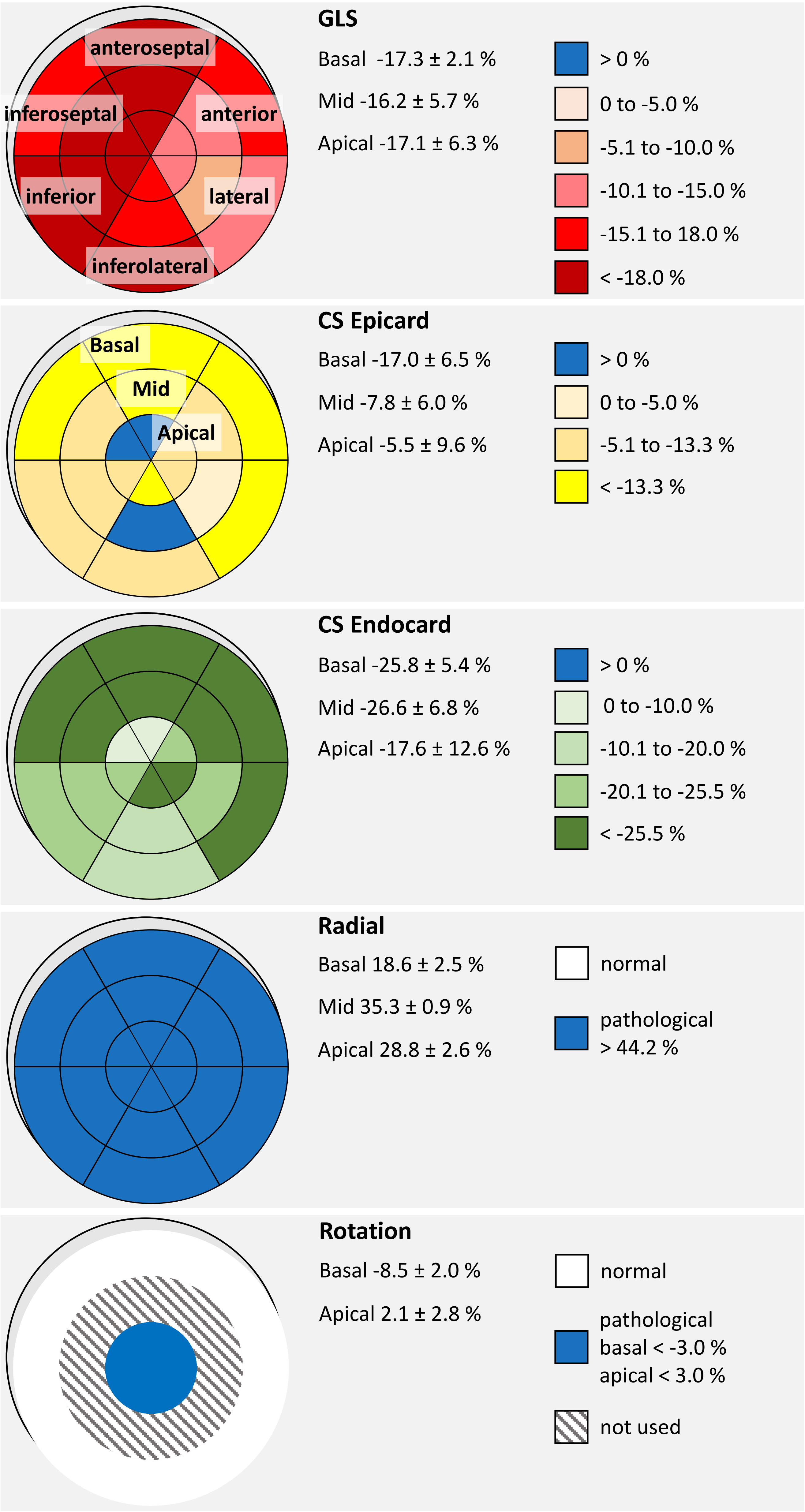
anteroseptal
inferoseptal
anterior
inferior
lateral
inferolateral
GLS
Basal -17.3 ± 2.1 %
Mid -16.2 ± 5.7 %
Apical -17.1 ± 6.3 %
> 0 %
0 to -5.0 %
-5.1 to -10.0 %
-10.1 to -15.0 %
-15.1 to 18.0 %
< -18.0 %
Basal
Mid
Apical
CS Epicard
Basal -17.0 ± 6.5 %
Mid -7.8 ± 6.0 %
Apical -5.5 ± 9.6 %
> 0 %
0 to -5.0 %
-5.1 to -13.3 %
< -13.3 %
CS Endocard
Basal -25.8 ± 5.4 %
Mid -26.6 ± 6.8 %
Apical -17.6 ± 12.6 %
> 0 %
0 to -10.0 %
-10.1 to -20.0 %
-20.1 to -25.5 %
< -25.5 %
Radial
Basal 18.6 ± 2.5 %
Mid 35.3 ± 0.9 %
Apical 28.8 ± 2.6 %
normal
pathological
> 44.2 %
Rotation
Basal -8.5 ± 2.0 %
Apical 2.1 ± 2.8 %
normal
pathological
basal < -3.0 %
apical < 3.0 %
not used

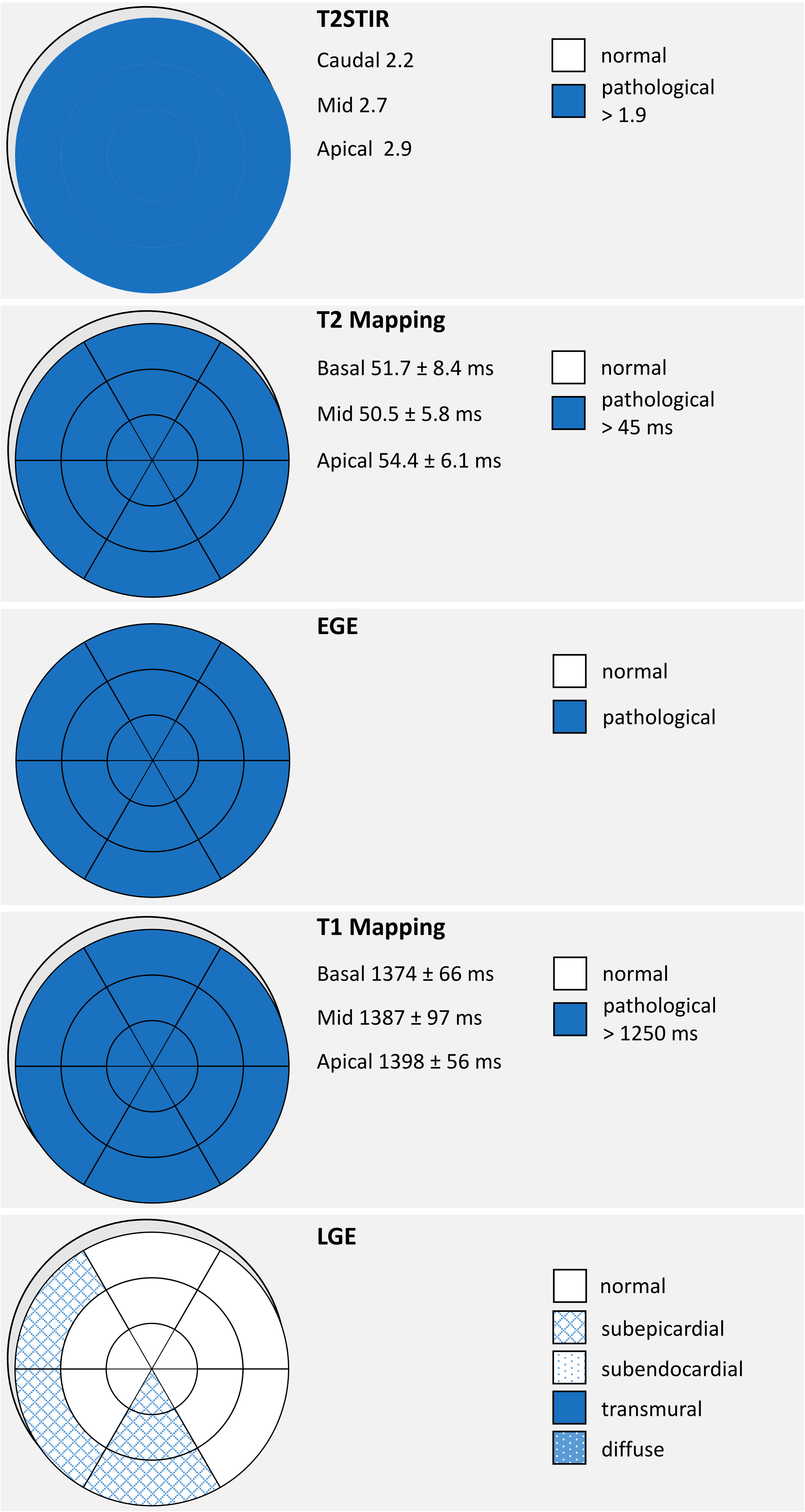
T2STIR
Caudal 2.2
Mid 2.7
Apical 2.9
normal
pathological
> 1.9
T2 Mapping
Basal 51.7 ± 8.4 ms
Mid 50.5 ± 5.8 ms
Apical 54.4 ± 6.1 ms
normal
pathological
> 45 ms
EGE
normal
pathological
T1 Mapping
Basal 1374 ± 66 ms
Mid 1387 ± 97 ms
Apical 1398 ± 56 ms
normal
pathological
> 1250 ms
LGE
normal
subepicardial
subendocardial
transmural
diffuse

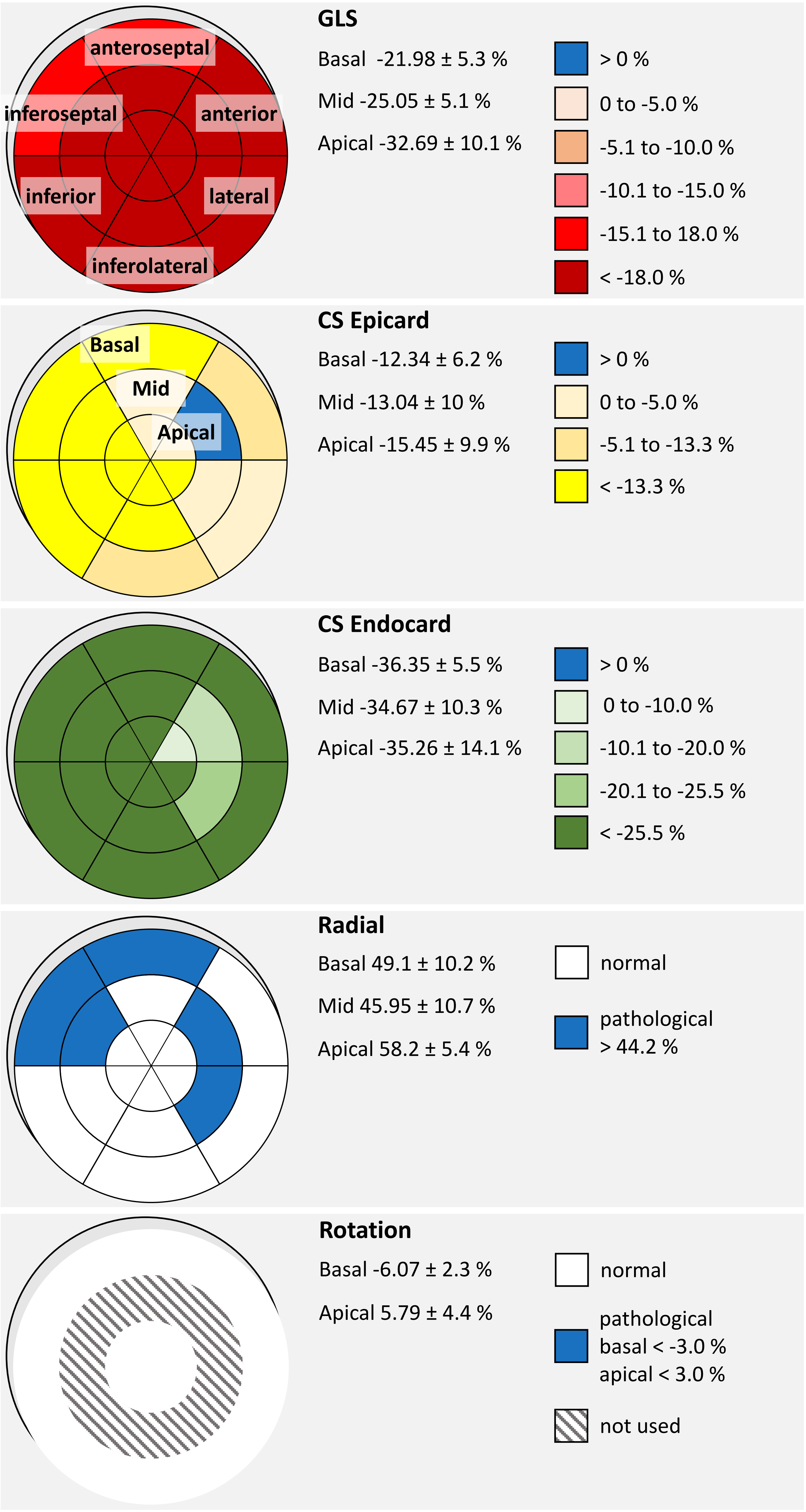
anteroseptal
inferoseptal
anterior
inferior
lateral
inferolateral
GLS
Basal -21.98 ± 5.3 %
Mid -25.05 ± 5.1 %
Apical -32.69 ± 10.1 %
> 0 %
0 to -5.0 %
-5.1 to -10.0 %
-10.1 to -15.0 %
-15.1 to 18.0 %
< -18.0 %
Basal
Mid
Apical
CS Epicard
Basal -12.34 ± 6.2 %
Mid -13.04 ± 10 %
Apical -15.45 ± 9.9 %
> 0 %
0 to -5.0 %
-5.1 to -13.3 %
< -13.3 %
CS Endocard
Basal -36.35 ± 5.5 %
Mid -34.67 ± 10.3 %
Apical -35.26 ± 14.1 %
> 0 %
0 to -10.0 %
-10.1 to -20.0 %
-20.1 to -25.5 %
< -25.5 %
Radial
Basal 49.1 ± 10.2 %
Mid 45.95 ± 10.7 %
Apical 58.2 ± 5.4 %
normal
pathological
> 44.2 %
Rotation
Basal -6.07 ± 2.3 %
Apical 5.79 ± 4.4 %
normal
pathological
basal < -3.0 %
apical < 3.0 %
not used

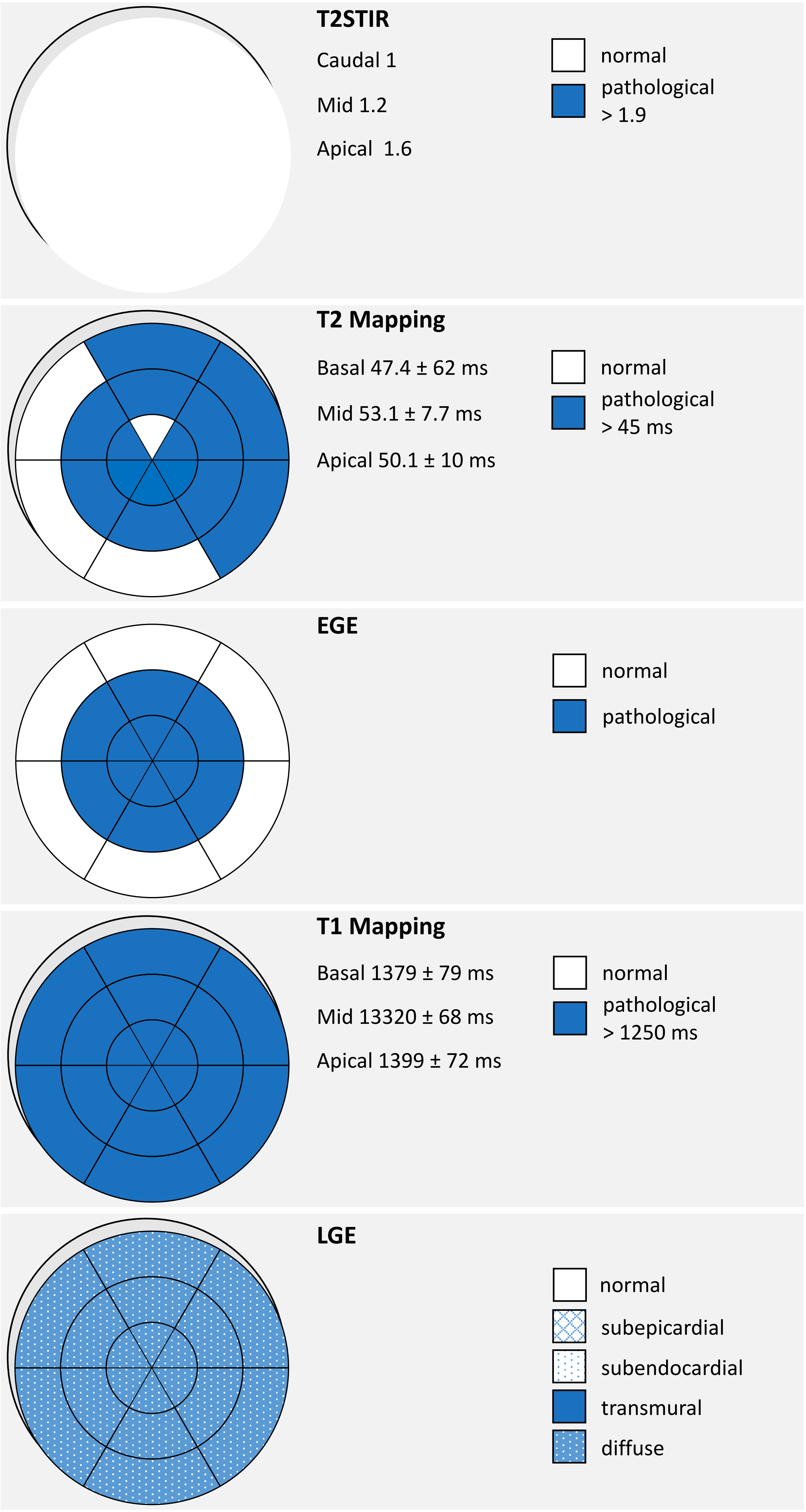

T2STIR
Caudal 1
Mid 1.2
Apical 1.6
normal
pathological > 1.9
T2 Mapping
Basal 47.4 ± 62 ms
Mid 53.1 ± 7.7 ms
Apical 50.1 ± 10 ms
normal
pathological > 45 ms
EGE
normal
pathological
T1 Mapping
Basal 1379 ± 79 ms
Mid 13320 ± 68 ms
Apical 1399 ± 72 ms
normal
pathological > 1250 ms
LGE
normal
subepicardial
subendocardial
transmural
diffuse

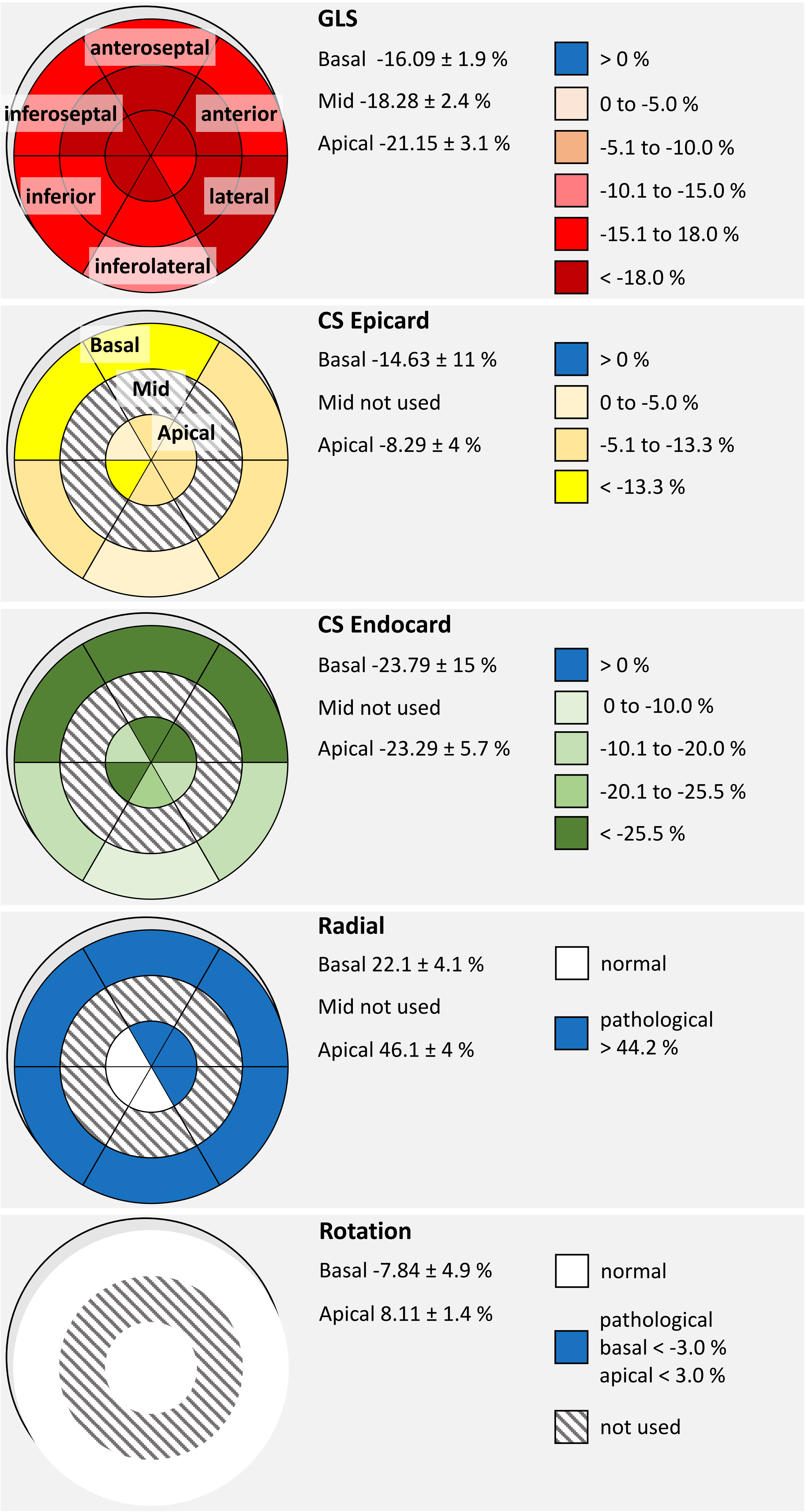
anteroseptal
inferoseptal
anterior
inferior
lateral
inferolateral
GLS
Basal -16.09 ± 1.9 %
Mid -18.28 ± 2.4 %
Apical -21.15 ± 3.1 %
> 0 %
0 to -5.0 %
-5.1 to -10.0 %
-10.1 to -15.0 %
-15.1 to 18.0 %
< -18.0 %
Basal
Mid
Apical
CS Epicard
Basal -14.63 ± 11 %
Mid not used
Apical -8.29 ± 4 %
> 0 %
0 to -5.0 %
-5.1 to -13.3 %
< -13.3 %
CS Endocard
Basal -23.79 ± 15 %
Mid not used
Apical -23.29 ± 5.7 %
> 0 %
0 to -10.0 %
-10.1 to -20.0 %
-20.1 to -25.5 %
< -25.5 %
Radial
Basal 22.1 ± 4.1 %
Mid not used
Apical 46.1 ± 4 %
normal
pathological
> 44.2 %
Rotation
Basal -7.84 ± 4.9 %
Apical 8.11 ± 1.4 %
normal
pathological
basal < -3.0 %
apical < 3.0 %
not used

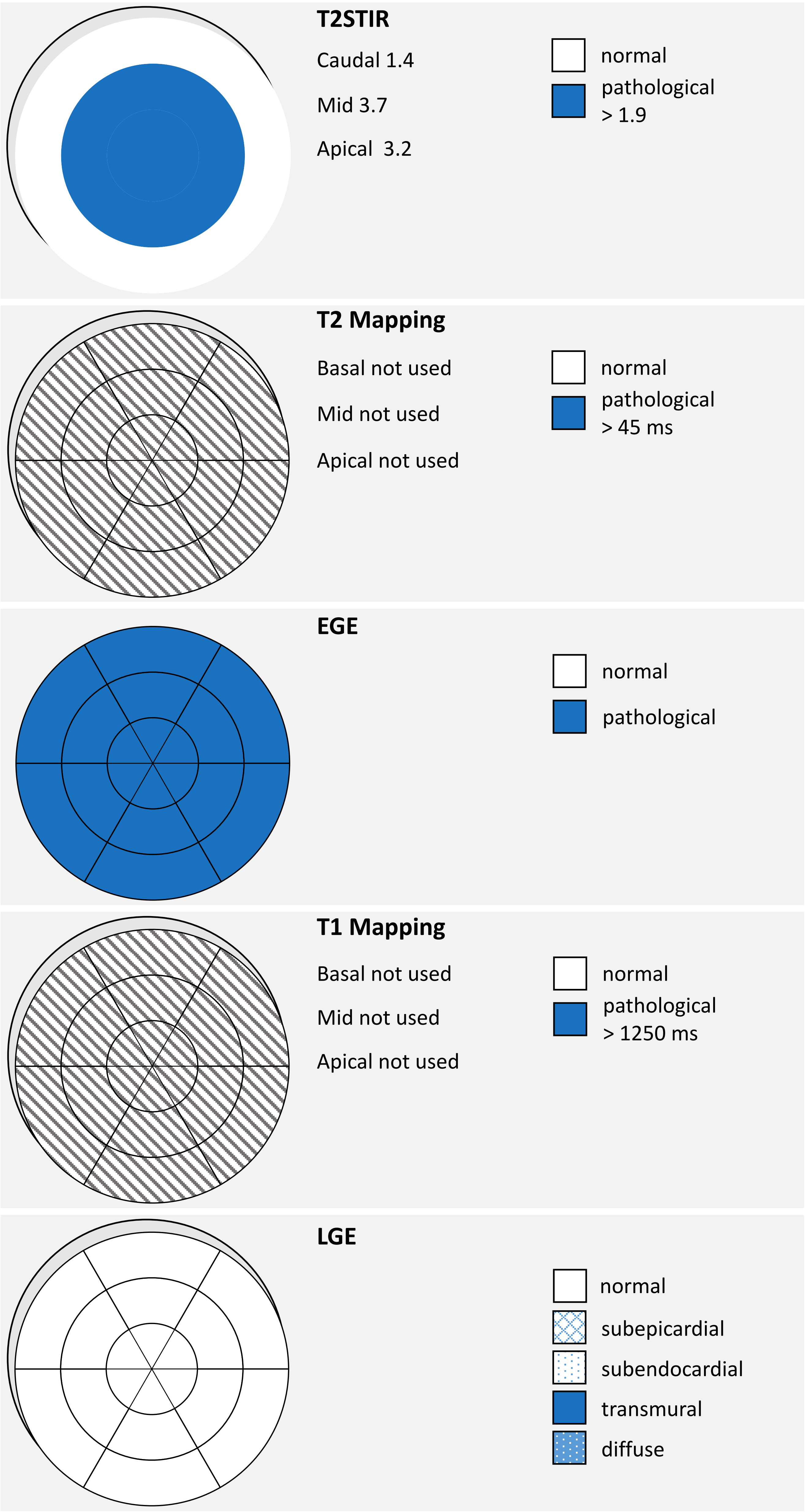
T2STIR
Caudal 1.4
Mid 3.7
Apical 3.2
normal
pathological > 1.9
T2 Mapping
Basal not used
Mid not used
Apical not used
normal
pathological > 45 ms
EGE
normal
pathological
T1 Mapping
Basal not used
Mid not used
Apical not used
normal
pathological > 1250 ms
LGE
normal
subepicardial
subendocardial
transmural
diffuse

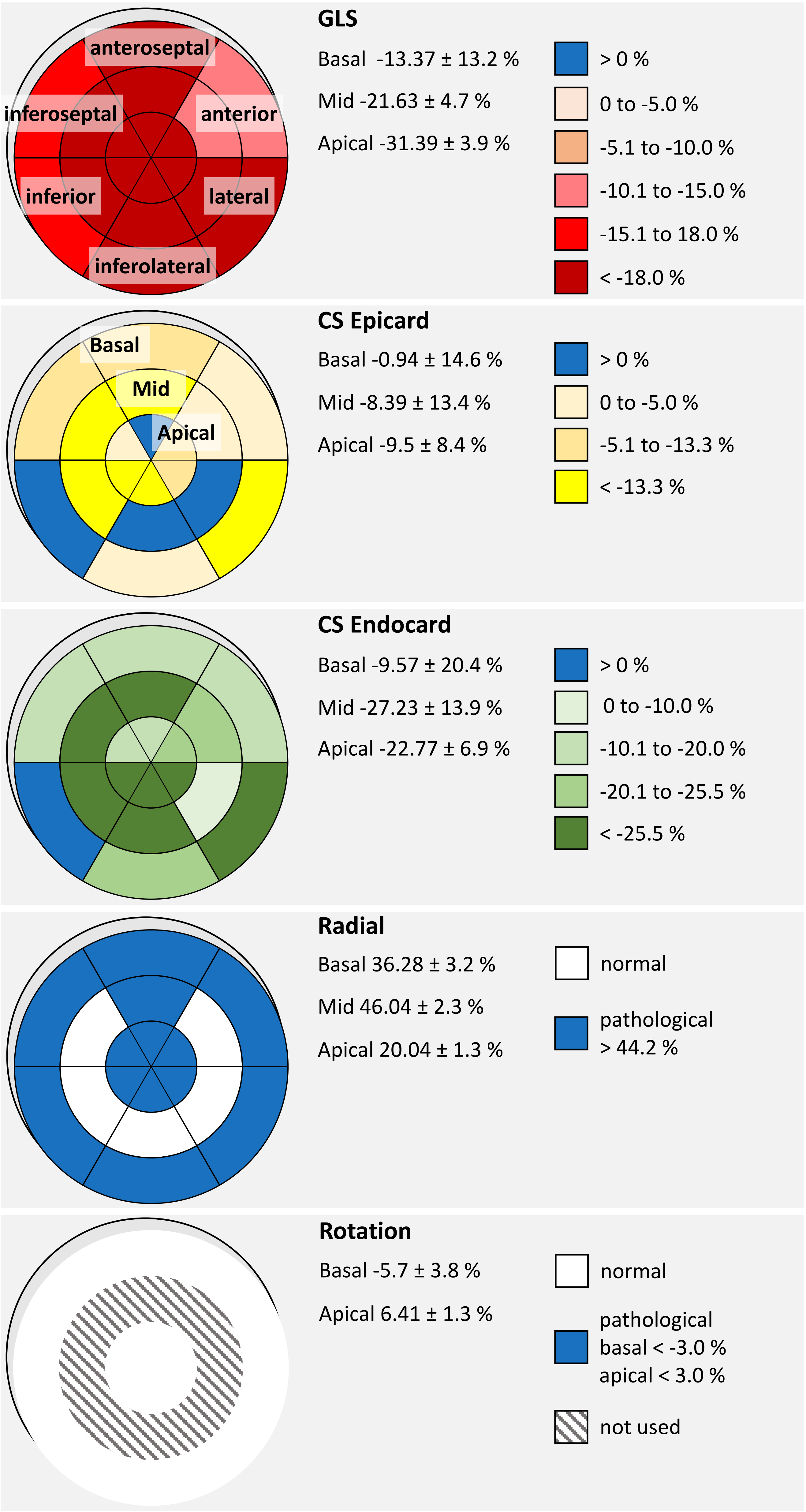

anteroseptal
inferoseptal
anterior
inferior
lateral
inferolateral
GLS
Basal -13.37 ± 13.2 %
Mid -21.63 ± 4.7 %
Apical -31.39 ± 3.9 %
> 0 %
0 to -5.0 %
-5.1 to -10.0 %
-10.1 to -15.0 %
-15.1 to 18.0 %
< -18.0 %
Basal
Mid
Apical
CS Epicard
Basal -0.94 ± 14.6 %
Mid -8.39 ± 13.4 %
Apical -9.5 ± 8.4 %
> 0 %
0 to -5.0 %
-5.1 to -13.3 %
< -13.3 %
CS Endocard
Basal -9.57 ± 20.4 %
Mid -27.23 ± 13.9 %
Apical -22.77 ± 6.9 %
> 0 %
0 to -10.0 %
-10.1 to -20.0 %
-20.1 to -25.5 %
< -25.5 %
Radial
Basal 36.28 ± 3.2 %
Mid 46.04 ± 2.3 %
Apical 20.04 ± 1.3 %
normal
pathological
> 44.2 %
Rotation
Basal -5.7 ± 3.8 %
Apical 6.41 ± 1.3 %
normal
pathological
basal < -3.0 %
apical < 3.0 %
not used

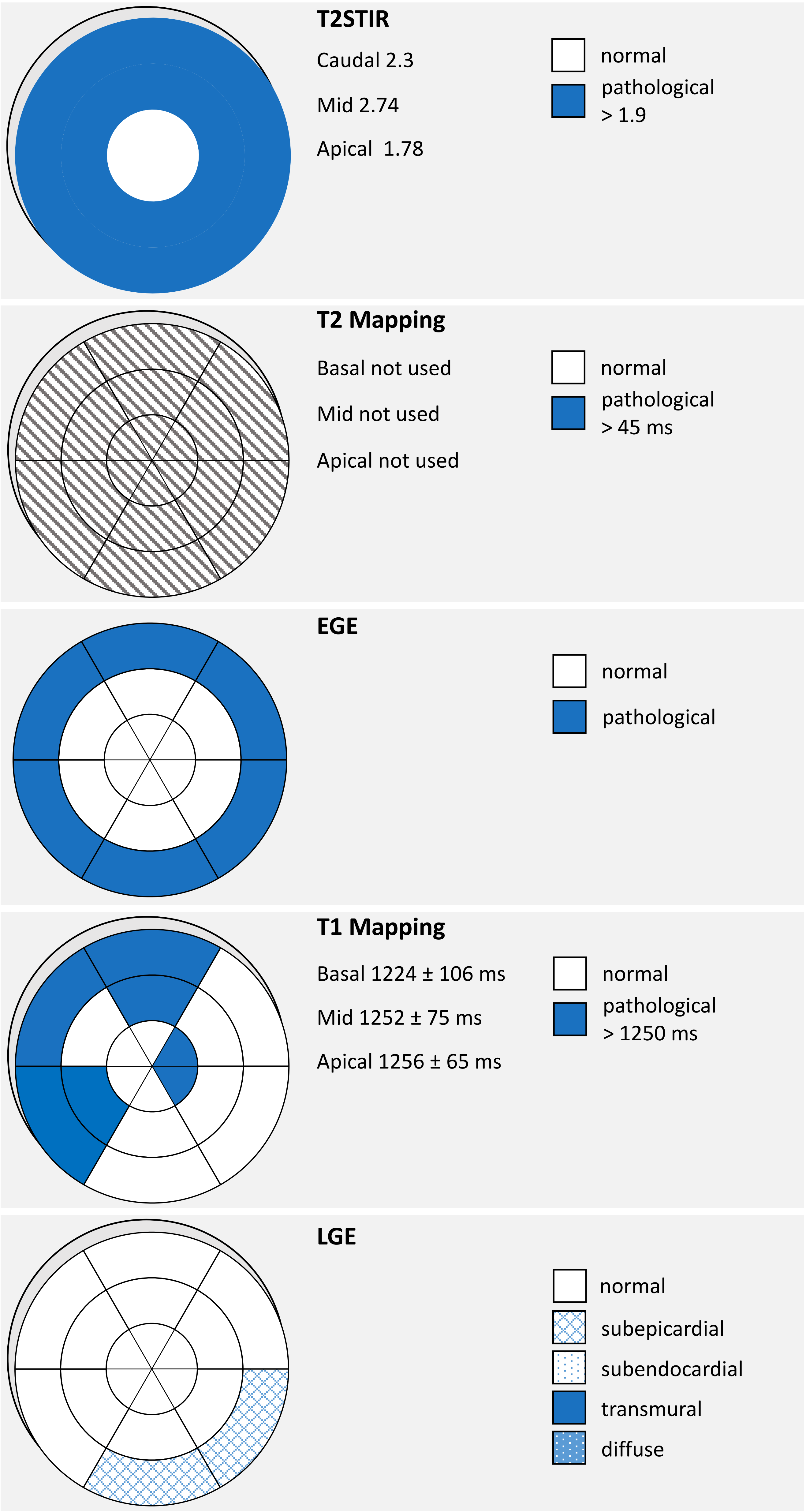
T2STIR
Caudal 2.3
Mid 2.74
Apical 1.78
normal
pathological > 1.9
T2 Mapping
Basal not used
Mid not used
Apical not used
normal
pathological > 45 ms
EGE
normal
pathological
T1 Mapping
Basal 1224 ± 106 ms
Mid 1252 ± 75 ms
Apical 1256 ± 65 ms
normal
pathological > 1250 ms
LGE
normal
subepicardial
subendocardial
transmural
diffuse

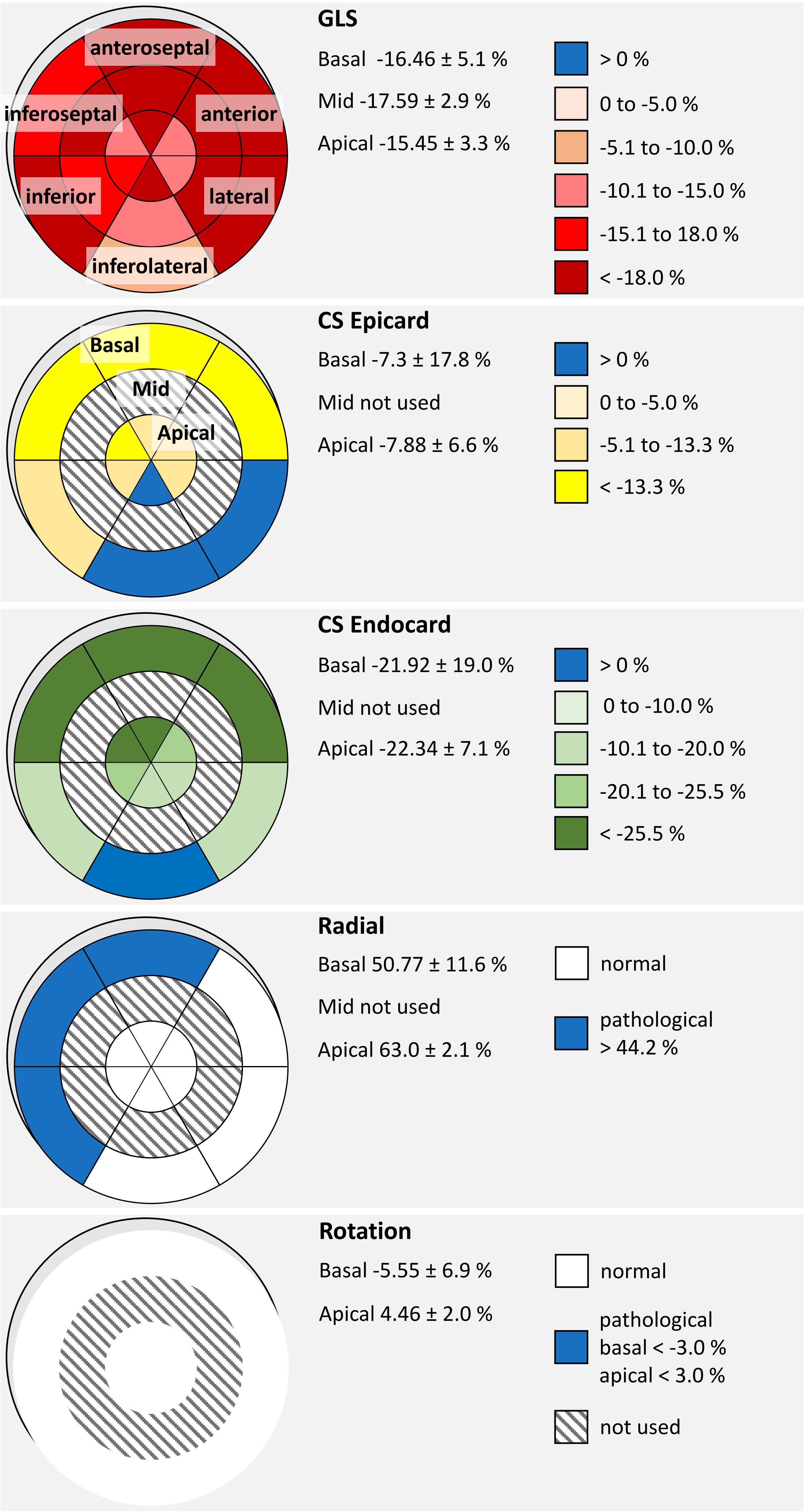
anteroseptal
inferoseptal
anterior
inferior
lateral
inferolateral
GLS
Basal -16.46 ± 5.1 %
Mid -17.59 ± 2.9 %
Apical -15.45 ± 3.3 %
> 0 %
0 to -5.0 %
-5.1 to -10.0 %
-10.1 to -15.0 %
-15.1 to 18.0 %
< -18.0 %
Basal
Mid
Apical
CS Epicard
Basal -7.3 ± 17.8 %
Mid not used
Apical -7.88 ± 6.6 %
> 0 %
0 to -5.0 %
-5.1 to -13.3 %
< -13.3 %
CS Endocard
Basal -21.92 ± 19.0 %
Mid not used
Apical -22.34 ± 7.1 %
> 0 %
0 to -10.0 %
-10.1 to -20.0 %
-20.1 to -25.5 %
< -25.5 %
Radial
Basal 50.77 ± 11.6 %
Mid not used
Apical 63.0 ± 2.1 %
normal
pathological
> 44.2 %
Rotation
Basal -5.55 ± 6.9 %
Apical 4.46 ± 2.0 %
normal
pathological
basal < -3.0 %
apical < 3.0 %
not used

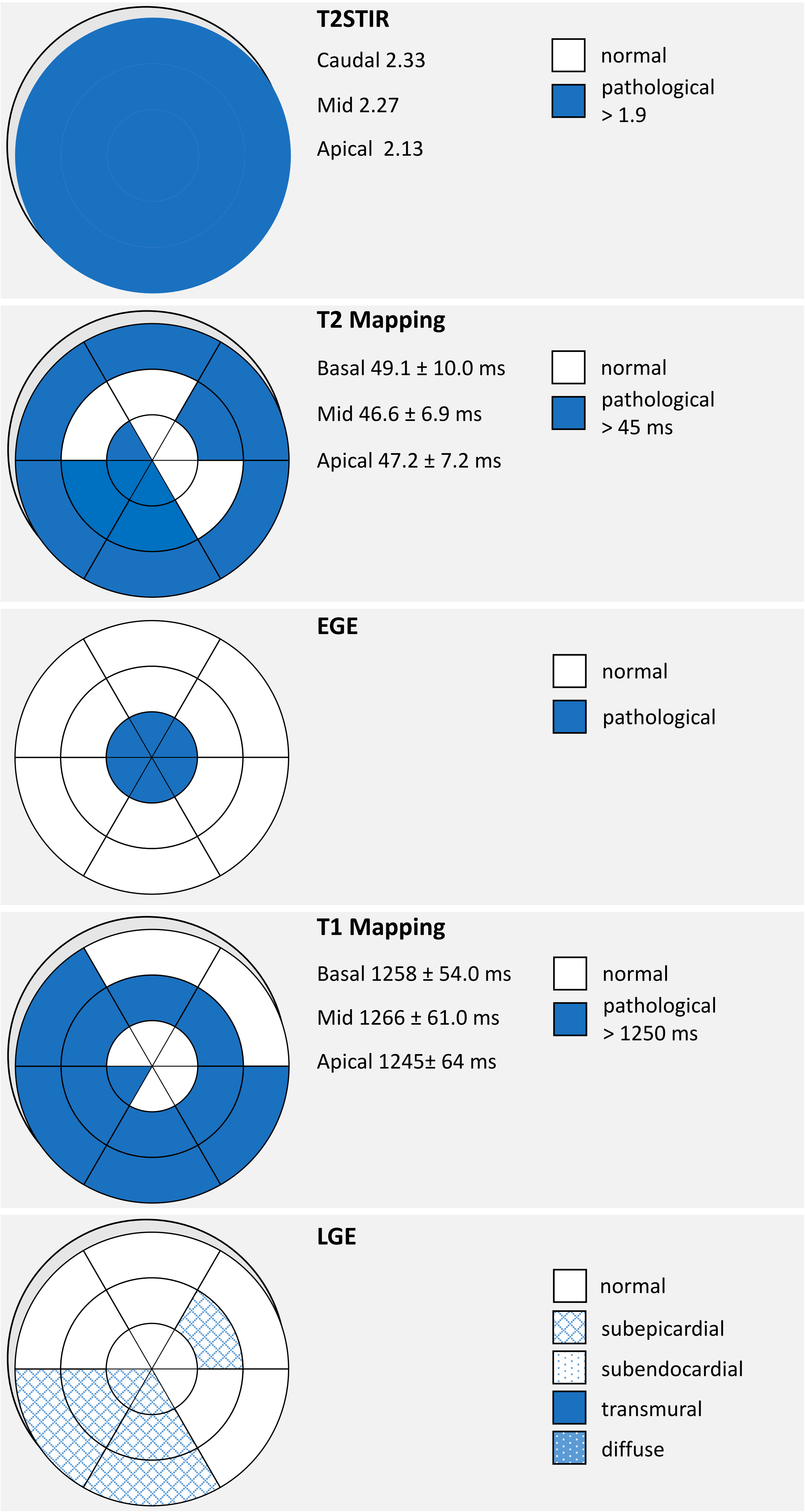
T2STIR
Caudal 2.33
Mid 2.27
Apical 2.13
normal
pathological
> 1.9
T2 Mapping
Basal 49.1 ± 10.0 ms
Mid 46.6 ± 6.9 ms
Apical 47.2 ± 7.2 ms
normal
pathological
> 45 ms
EGE
normal
pathological
T1 Mapping
Basal 1258 ± 54.0 ms
Mid 1266 ± 61.0 ms
Apical 1245± 64 ms
normal
pathological
> 1250 ms
LGE
normal
subepicardial
subendocardial
transmural
diffuse

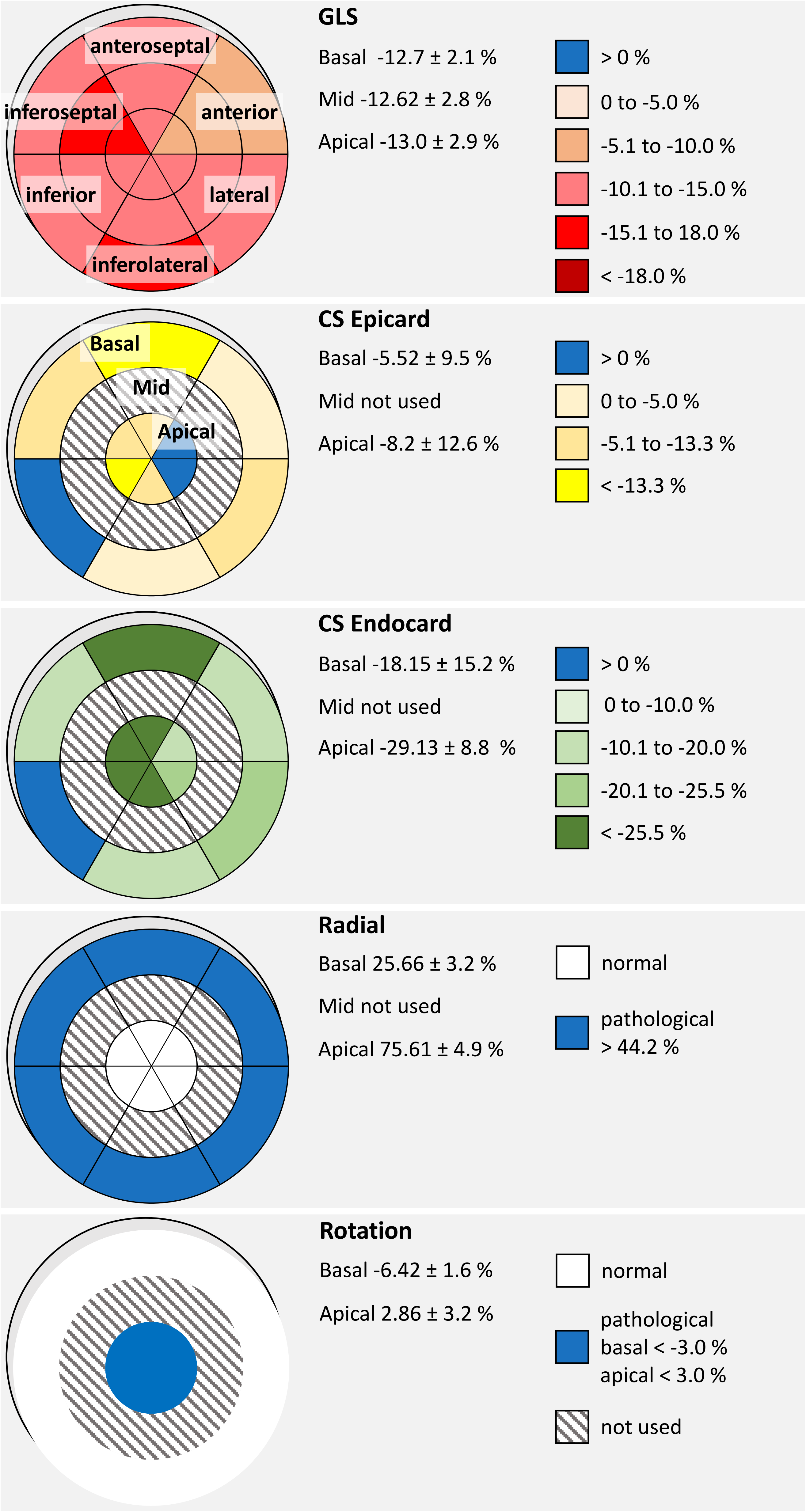

anteroseptal
inferoseptal
anterior
inferior
lateral
inferolateral
GLS
Basal -12.7 ± 2.1 %
Mid -12.62 ± 2.8 %
Apical -13.0 ± 2.9 %
> 0 %
0 to -5.0 %
-5.1 to -10.0 %
-10.1 to -15.0 %
-15.1 to 18.0 %
< -18.0 %
Basal
Mid
Apical
CS Epicard
Basal -5.52 ± 9.5 %
Mid not used
Apical -8.2 ± 12.6 %
> 0 %
0 to -5.0 %
-5.1 to -13.3 %
< -13.3 %
CS Endocard
Basal -18.15 ± 15.2 %
Mid not used
Apical -29.13 ± 8.8 %
> 0 %
0 to -10.0 %
-10.1 to -20.0 %
-20.1 to -25.5 %
< -25.5 %
Radial
Basal 25.66 ± 3.2 %
Mid not used
Apical 75.61 ± 4.9 %
normal
pathological
> 44.2 %
Rotation
Basal -6.42 ± 1.6 %
Apical 2.86 ± 3.2 %
normal
pathological
basal < -3.0 %
apical < 3.0 %
not used

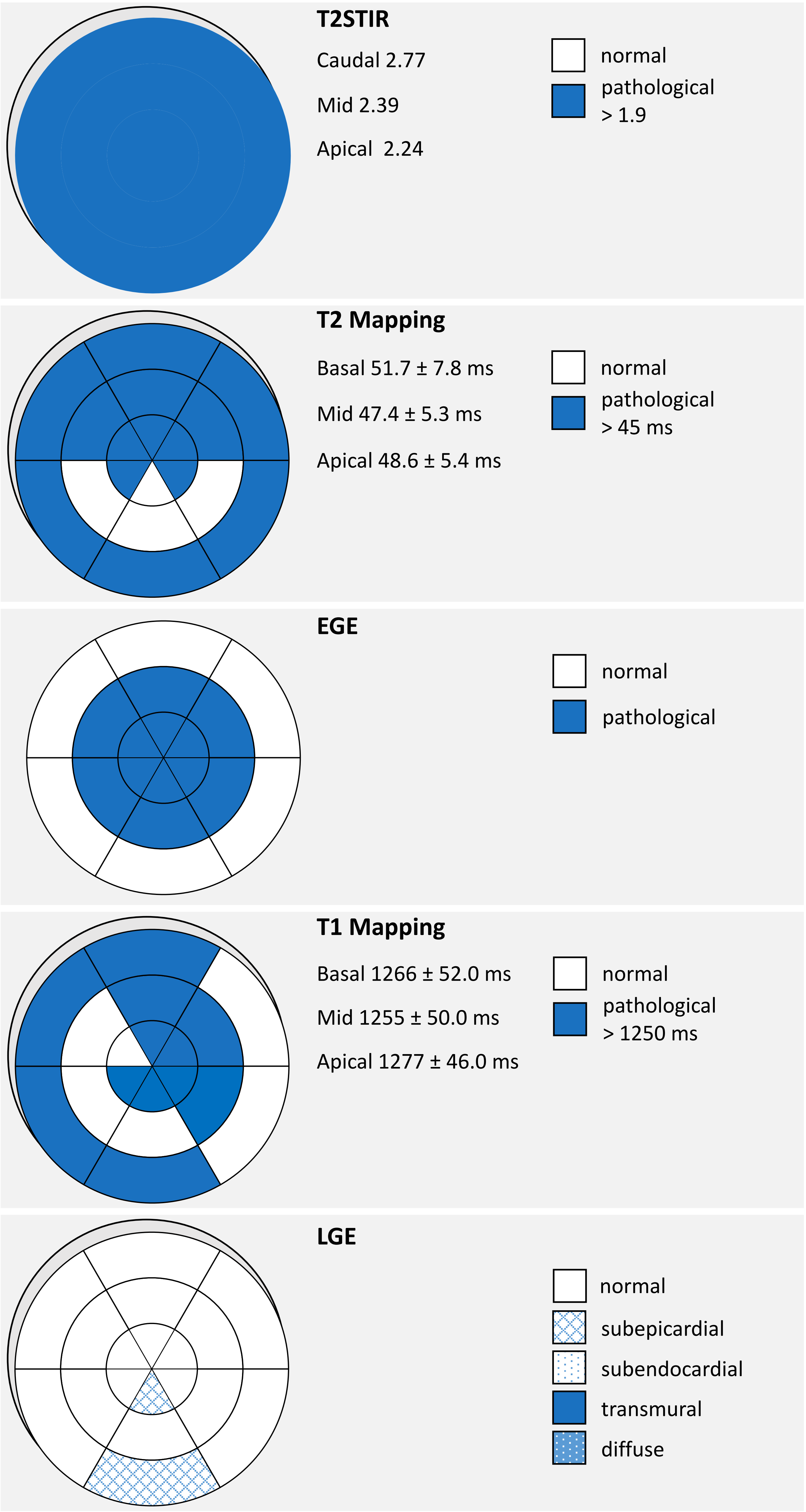

T2STIR
Caudal 2.77
Mid 2.39
Apical 2.24
normal
pathological > 1.9
T2 Mapping
Basal 51.7 ± 7.8 ms
Mid 47.4 ± 5.3 ms
Apical 48.6 ± 5.4 ms
normal
pathological > 45 ms
EGE
normal
pathological
T1 Mapping
Basal 1266 ± 52.0 ms
Mid 1255 ± 50.0 ms
Apical 1277 ± 46.0 ms
normal
pathological > 1250 ms
LGE
normal
subepicardial
subendocardial
transmural
diffuse

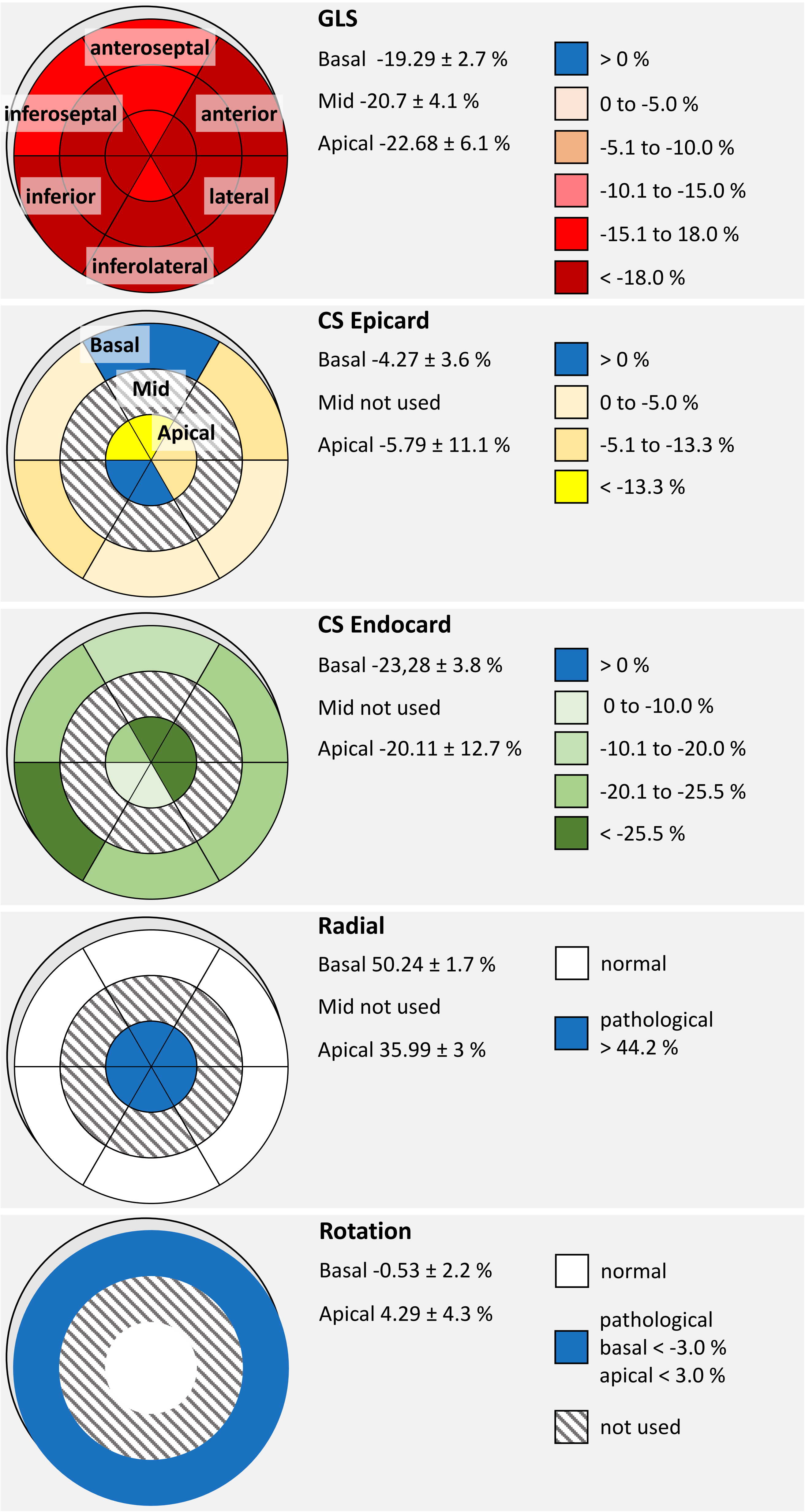
anteroseptal
inferoseptal
anterior
inferior
lateral
inferolateral
GLS
Basal -19.29 ± 2.7 %
Mid -20.7 ± 4.1 %
Apical -22.68 ± 6.1 %
> 0 %
0 to -5.0 %
-5.1 to -10.0 %
-10.1 to -15.0 %
-15.1 to 18.0 %
< -18.0 %
Basal
Mid
Apical
CS Epicard
Basal -4.27 ± 3.6 %
Mid not used
Apical -5.79 ± 11.1 %
> 0 %
0 to -5.0 %
-5.1 to -13.3 %
< -13.3 %
CS Endocard
Basal -23,28 ± 3.8 %
Mid not used
Apical -20.11 ± 12.7 %
> 0 %
0 to -10.0 %
-10.1 to -20.0 %
-20.1 to -25.5 %
< -25.5 %
Radial
Basal 50.24 ± 1.7 %
Mid not used
Apical 35.99 ± 3 %
normal
pathological
> 44.2 %
Rotation
Basal -0.53 ± 2.2 %
Apical 4.29 ± 4.3 %
normal
pathological
basal < -3.0 %
apical < 3.0 %
not used

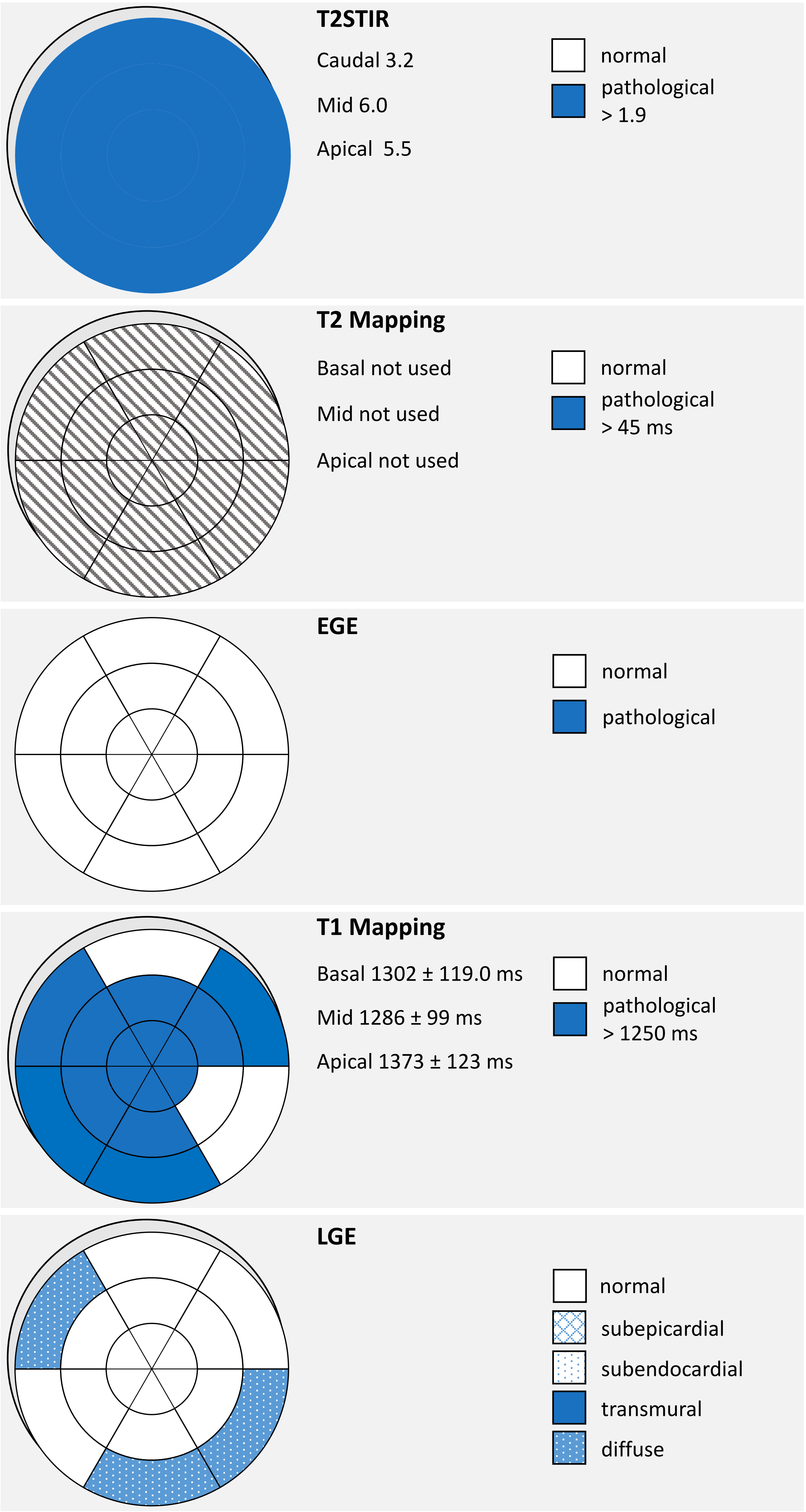
T2STIR
Caudal 3.2
Mid 6.0
Apical 5.5
normal
pathological
> 1.9
T2 Mapping
Basal not used
Mid not used
Apical not used
normal
pathological
> 45 ms
EGE
normal
pathological
T1 Mapping
Basal 1302 ± 119.0 ms
Mid 1286 ± 99 ms
Apical 1373 ± 123 ms
normal
pathological
> 1250 ms
LGE
normal
subepicardial
subendocardial
transmural
diffuse

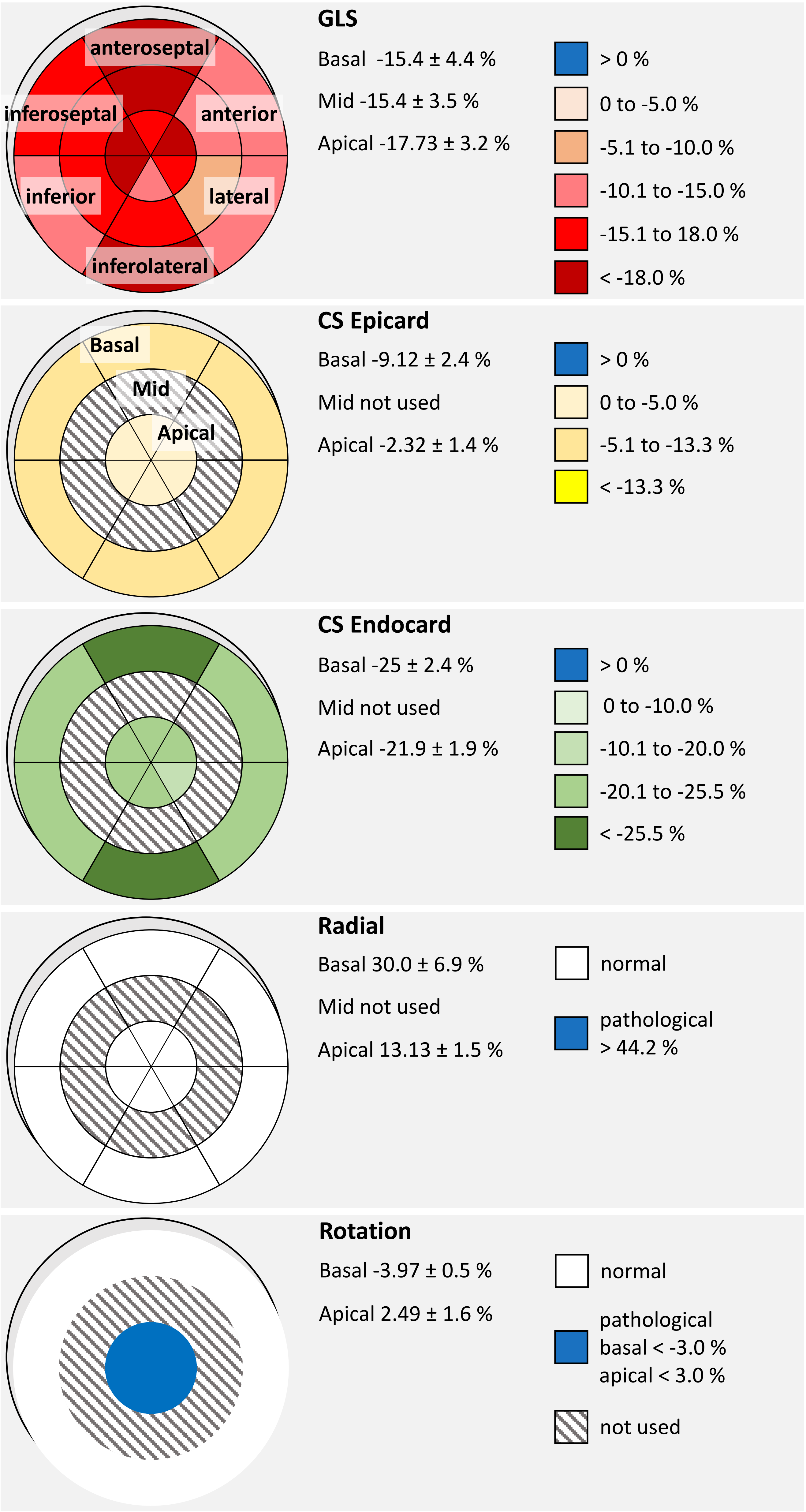
anteroseptal
inferoseptal
anterior
inferior
lateral
inferolateral
GLS
Basal -15.4 ± 4.4 %
Mid -15.4 ± 3.5 %
Apical -17.73 ± 3.2 %
> 0 %
0 to -5.0 %
-5.1 to -10.0 %
-10.1 to -15.0 %
-15.1 to 18.0 %
< -18.0 %
Basal
Mid
Apical
CS Epicard
Basal -9.12 ± 2.4 %
Mid not used
Apical -2.32 ± 1.4 %
> 0 %
0 to -5.0 %
-5.1 to -13.3 %
< -13.3 %
CS Endocard
Basal -25 ± 2.4 %
Mid not used
Apical -21.9 ± 1.9 %
> 0 %
0 to -10.0 %
-10.1 to -20.0 %
-20.1 to -25.5 %
< -25.5 %
Radial
Basal 30.0 ± 6.9 %
Mid not used
Apical 13.13 ± 1.5 %
normal
pathological
> 44.2 %
Rotation
Basal -3.97 ± 0.5 %
Apical 2.49 ± 1.6 %
normal
pathological
basal < -3.0 %
apical < 3.0 %
not used

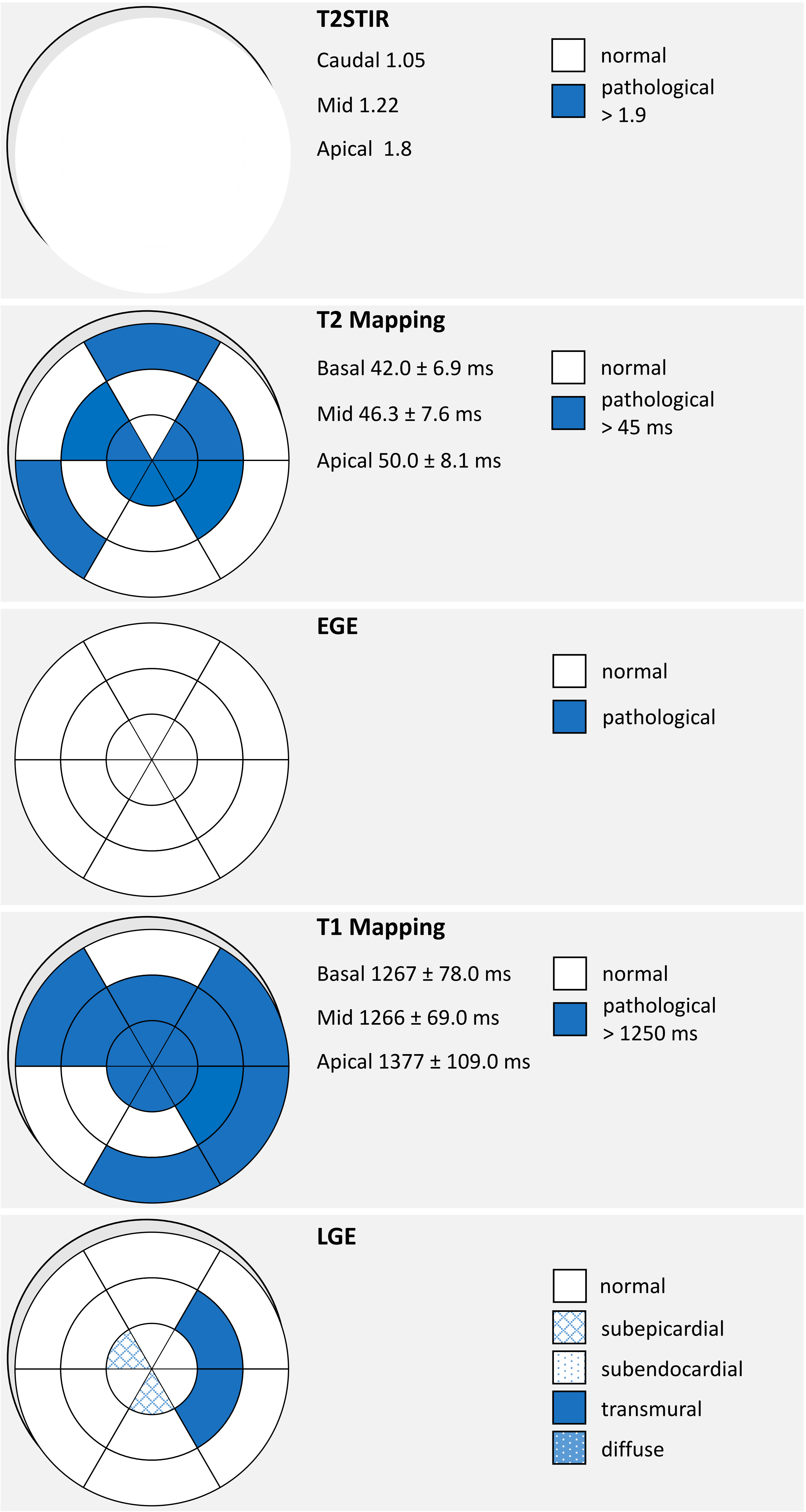
T2STIR
Caudal 1.05
Mid 1.22
Apical 1.8
normal
pathological
> 1.9
T2 Mapping
Basal 42.0 ± 6.9 ms
Mid 46.3 ± 7.6 ms
Apical 50.0 ± 8.1 ms
normal
pathological
> 45 ms
EGE
normal
pathological
T1 Mapping
Basal 1267 ± 78.0 ms
Mid 1266 ± 69.0 ms
Apical 1377 ± 109.0 ms
normal
pathological
> 1250 ms
LGE
normal
subepicardial
subendocardial
transmural
diffuse

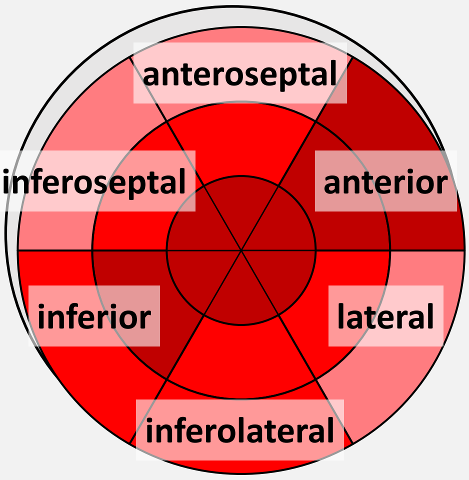

GLS

Basal -16.15 ± 3.2 %

Mid -17.85 ± 2.2 %

Apical -21.11 ± 2.5 %

- > 0 %
- 0 to -5.0 %
- -5.1 to -10.0 %
- -10.1 to -15.0 %
- -15.1 to 18.0 %
- < -18.0 %

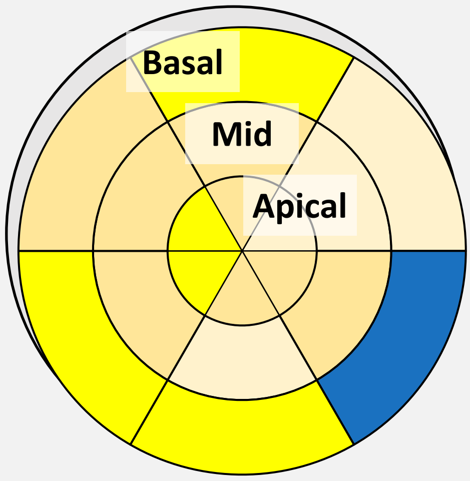

CS Epicard

Basal -10.47 ± 8.7 %

Mid -8.24 ± 4.9 %

Apical -9.81 ± 5.8 %

- > 0 %
- 0 to -5.0 %
- -5.1 to -13.3 %
- < -13.3 %

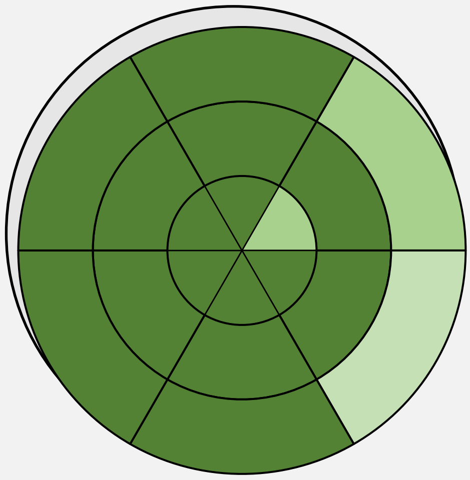

CS Endocard

Basal -30.1 ± 9.0 %

Mid -33.19 ± 4.8 %

Apical -35.06 ± 7.6 %

- > 0 %
- 0 to -10.0 %
- -10.1 to -20.0 %
- -20.1 to -25.5 %
- < -25.5 %

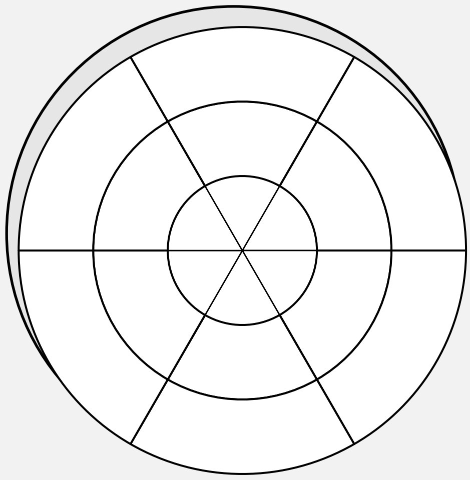

Radial

Basal 49.8 ± 3.4 %

Mid 75.85 ± 5.6 %

Apical 87.84 ± 6.9 %

- normal
- pathological > 44.2 %

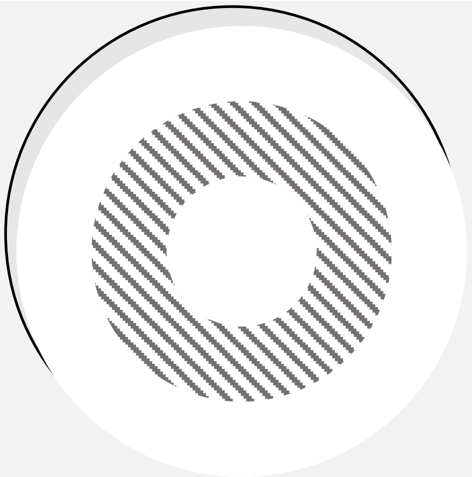

Rotation

Basal -4.85 ± 1.2 %

Apical 15.39 ± 3.5 %

- normal
- pathological basal < -3.0 % apical < 3.0 %
- not used

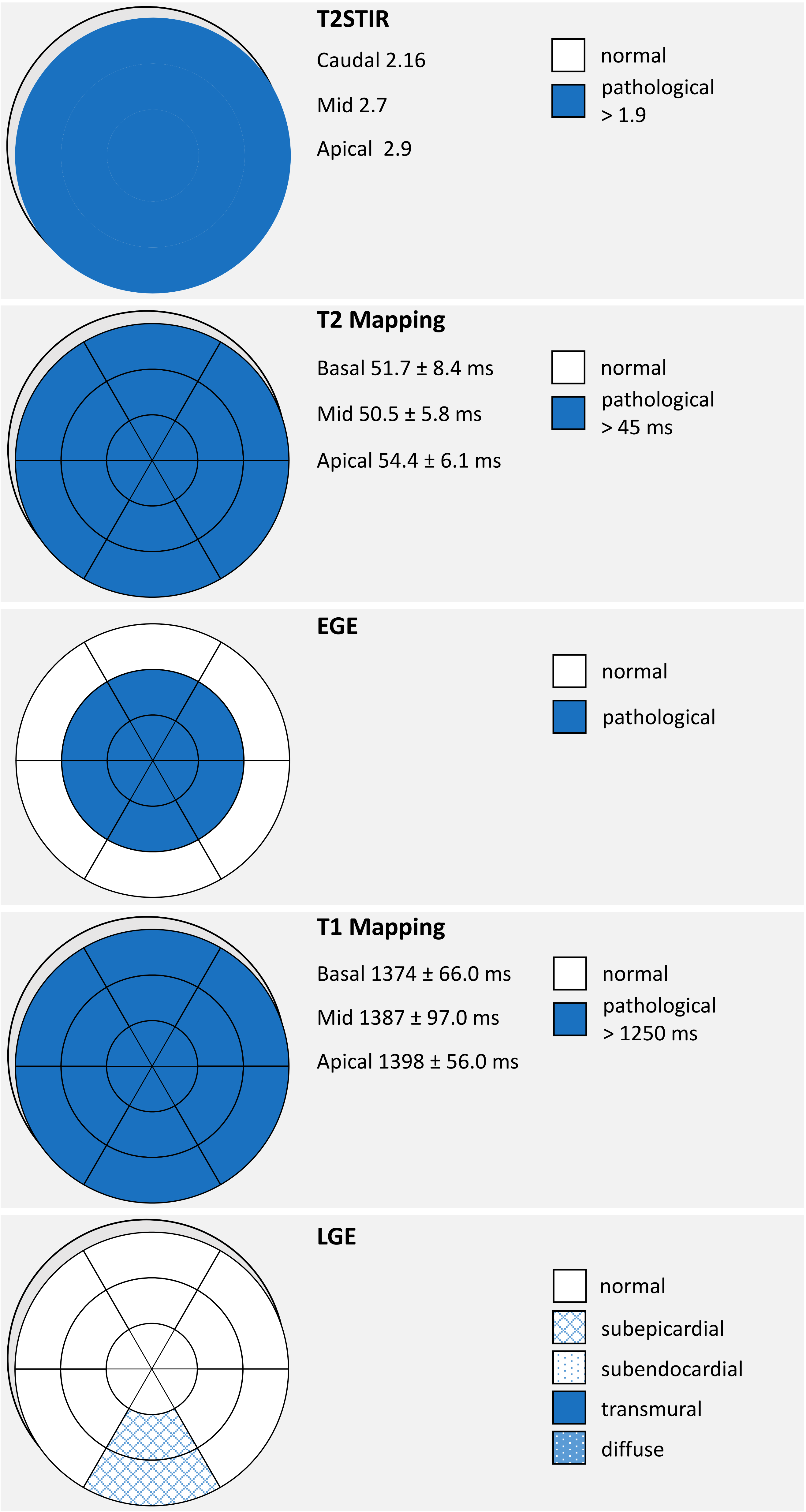
T2STIR
Caudal 2.16
Mid 2.7
Apical 2.9
normal
pathological
> 1.9
T2 Mapping
Basal 51.7 ± 8.4 ms
Mid 50.5 ± 5.8 ms
Apical 54.4 ± 6.1 ms
normal
pathological
> 45 ms
EGE
normal
pathological
T1 Mapping
Basal 1374 ± 66.0 ms
Mid 1387 ± 97.0 ms
Apical 1398 ± 56.0 ms
normal
pathological
> 1250 ms
LGE
normal
subepicardial
subendocardial
transmural
diffuse

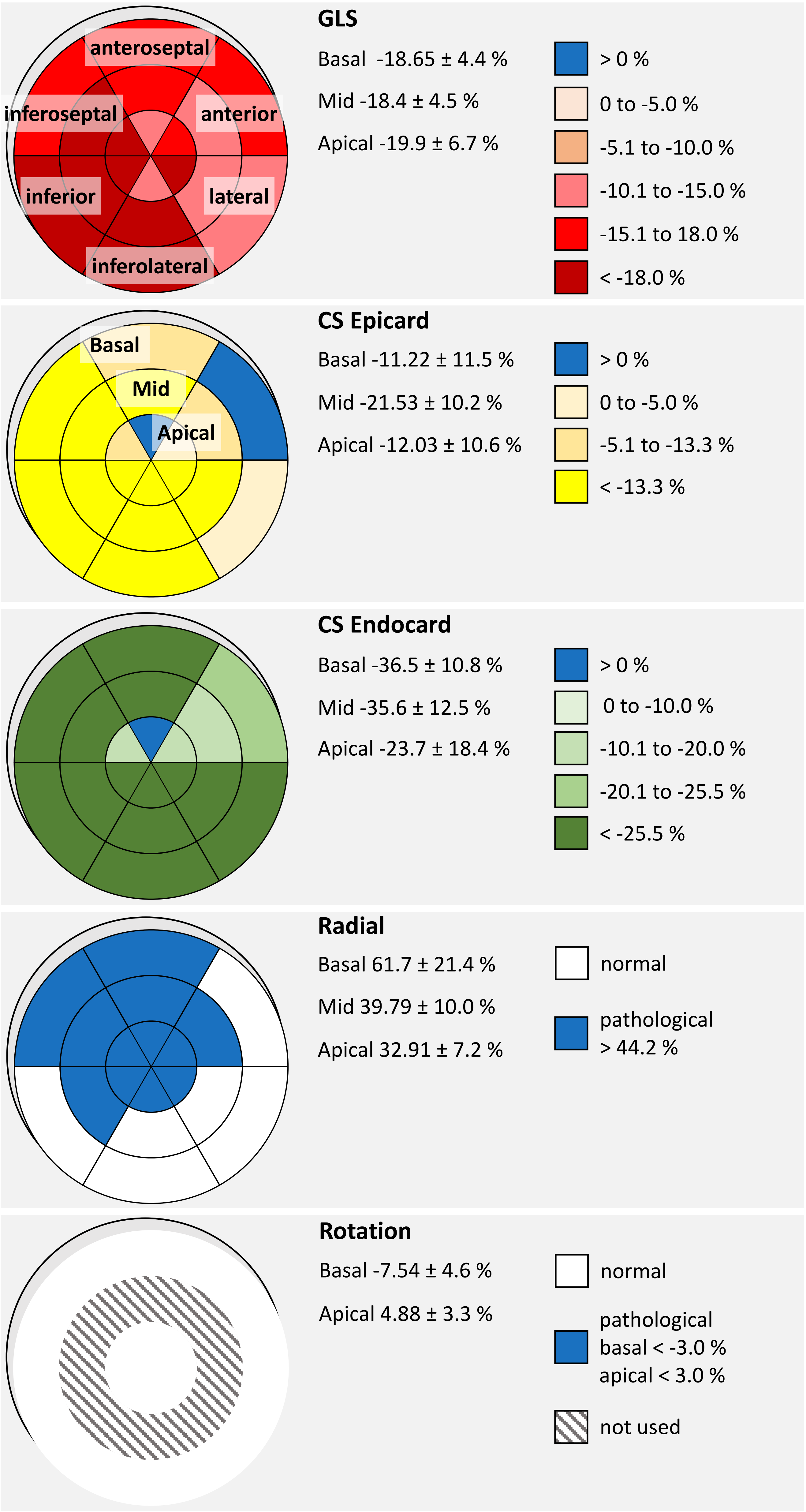
anteroseptal
inferoseptal
anterior
inferior
lateral
inferolateral
GLS
Basal -18.65 ± 4.4 %
Mid -18.4 ± 4.5 %
Apical -19.9 ± 6.7 %
> 0 %
0 to -5.0 %
-5.1 to -10.0 %
-10.1 to -15.0 %
-15.1 to 18.0 %
< -18.0 %
Basal
Mid
Apical
CS Epicard
Basal -11.22 ± 11.5 %
Mid -21.53 ± 10.2 %
Apical -12.03 ± 10.6 %
> 0 %
0 to -5.0 %
-5.1 to -13.3 %
< -13.3 %
CS Endocard
Basal -36.5 ± 10.8 %
Mid -35.6 ± 12.5 %
Apical -23.7 ± 18.4 %
> 0 %
0 to -10.0 %
-10.1 to -20.0 %
-20.1 to -25.5 %
< -25.5 %
Radial
Basal 61.7 ± 21.4 %
Mid 39.79 ± 10.0 %
Apical 32.91 ± 7.2 %
normal
pathological
> 44.2 %
Rotation
Basal -7.54 ± 4.6 %
Apical 4.88 ± 3.3 %
normal
pathological
basal < -3.0 %
apical < 3.0 %
not used

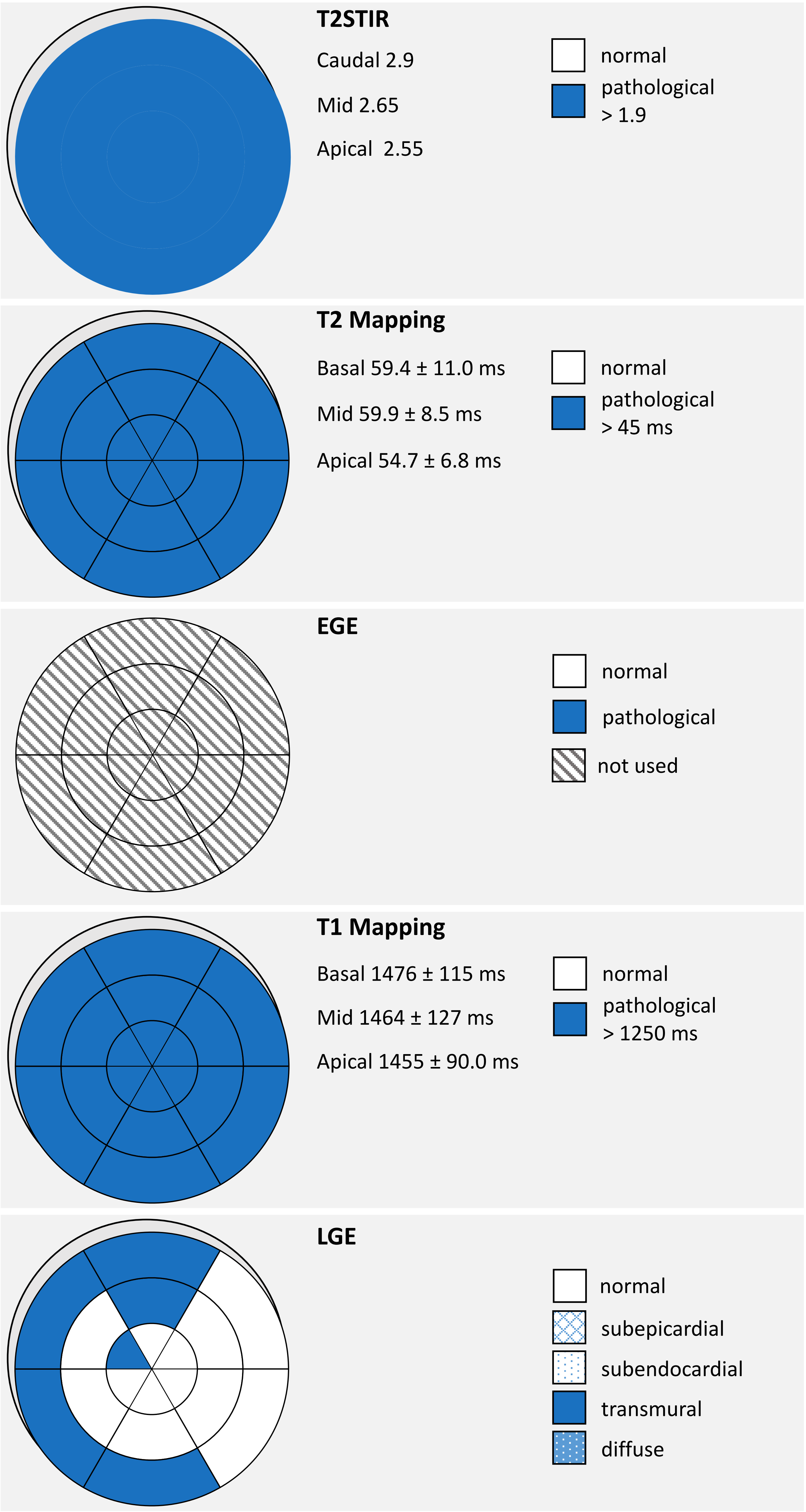
T2STIR
Caudal 2.9
Mid 2.65
Apical 2.55
normal
pathological
> 1.9
T2 Mapping
Basal 59.4 ± 11.0 ms
Mid 59.9 ± 8.5 ms
Apical 54.7 ± 6.8 ms
normal
pathological
> 45 ms
EGE
normal
pathological
not used
T1 Mapping
Basal 1476 ± 115 ms
Mid 1464 ± 127 ms
Apical 1455 ± 90.0 ms
normal
pathological
> 1250 ms
LGE
normal
subepicardial
subendocardial
transmural
diffuse

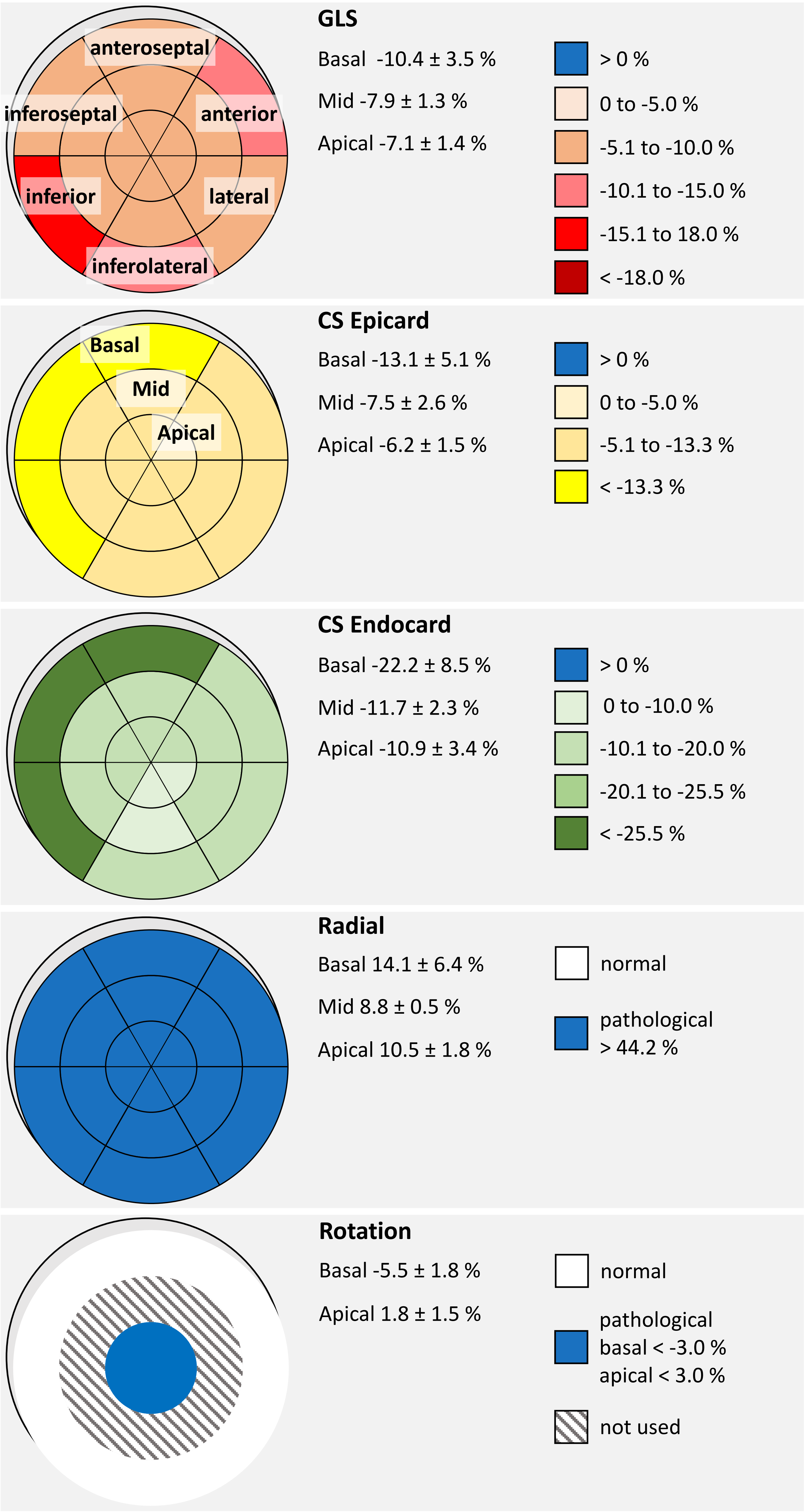
anteroseptal
inferoseptal
anterior
inferior
lateral
inferolateral
GLS
Basal -10.4 ± 3.5 %
Mid -7.9 ± 1.3 %
Apical -7.1 ± 1.4 %
> 0 %
0 to -5.0 %
-5.1 to -10.0 %
-10.1 to -15.0 %
-15.1 to 18.0 %
< -18.0 %
Basal
Mid
Apical
CS Epicard
Basal -13.1 ± 5.1 %
Mid -7.5 ± 2.6 %
Apical -6.2 ± 1.5 %
> 0 %
0 to -5.0 %
-5.1 to -13.3 %
< -13.3 %
CS Endocard
Basal -22.2 ± 8.5 %
Mid -11.7 ± 2.3 %
Apical -10.9 ± 3.4 %
> 0 %
0 to -10.0 %
-10.1 to -20.0 %
-20.1 to -25.5 %
< -25.5 %
Radial
Basal 14.1 ± 6.4 %
Mid 8.8 ± 0.5 %
Apical 10.5 ± 1.8 %
normal
pathological
> 44.2 %
Rotation
Basal -5.5 ± 1.8 %
Apical 1.8 ± 1.5 %
normal
pathological
basal < -3.0 %
apical < 3.0 %
not used

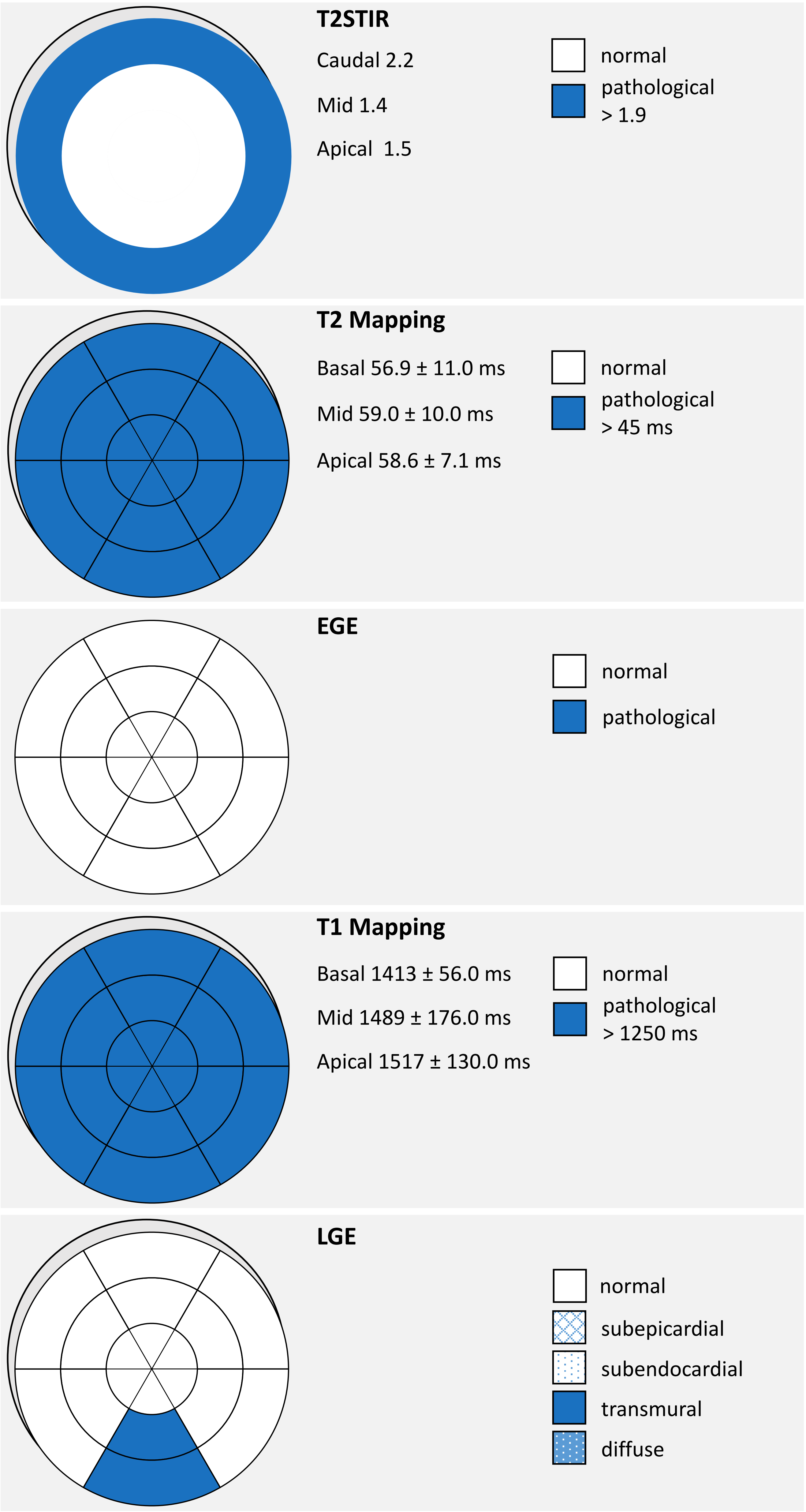
T2STIR
Caudal 2.2
Mid 1.4
Apical 1.5
normal
pathological > 1.9
T2 Mapping
Basal 56.9 ± 11.0 ms
Mid 59.0 ± 10.0 ms
Apical 58.6 ± 7.1 ms
normal
pathological > 45 ms
EGE
normal
pathological
T1 Mapping
Basal 1413 ± 56.0 ms
Mid 1489 ± 176.0 ms
Apical 1517 ± 130.0 ms
normal
pathological > 1250 ms
LGE
normal
subepicardial
subendocardial
transmural
diffuse

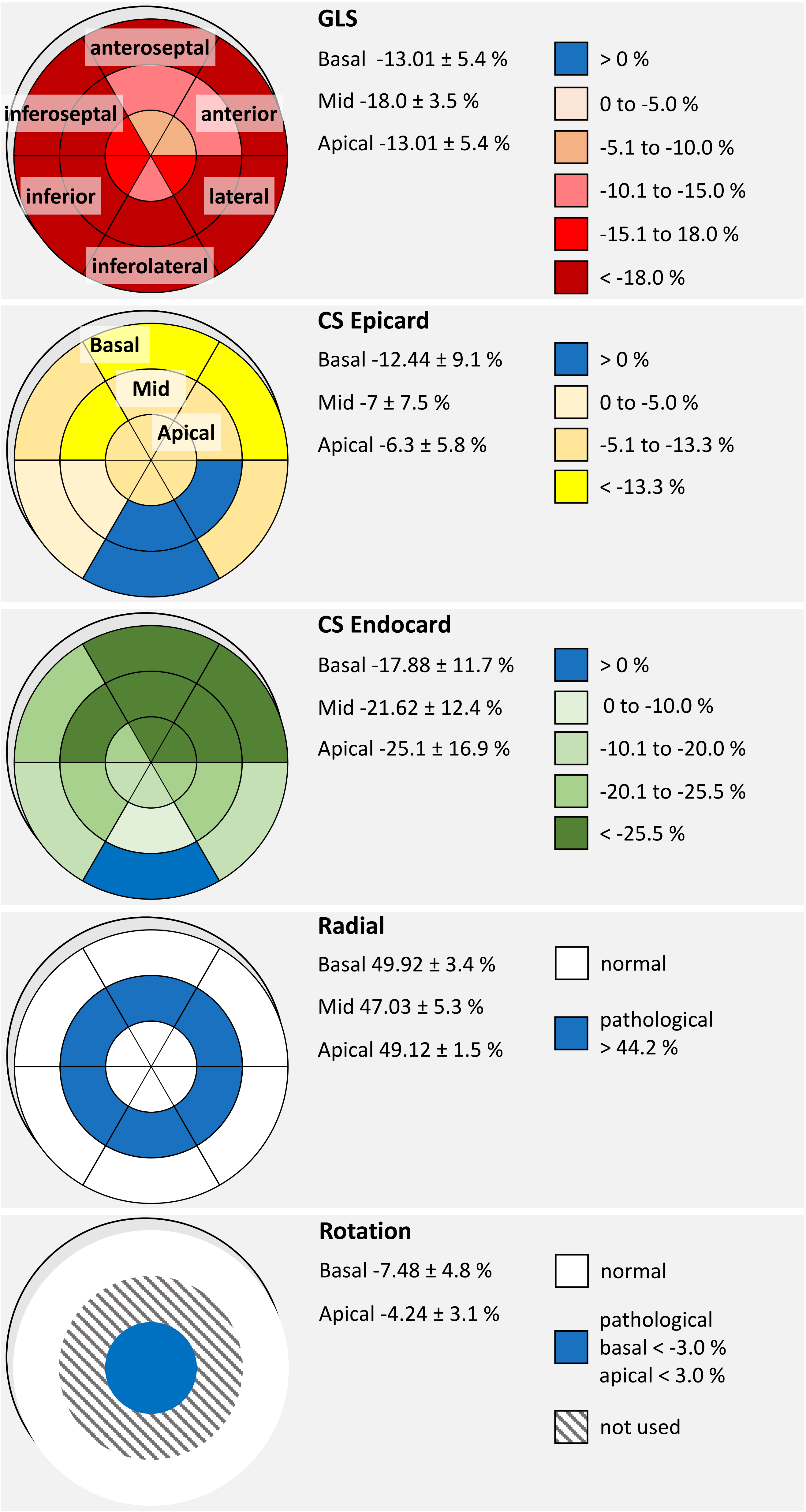

GLS
anteroseptal
inferoseptal
anterior
inferior
lateral
inferolateral
Basal -13.01 ± 5.4 %
Mid -18.0 ± 3.5 %
Apical -13.01 ± 5.4 %
> 0 %
0 to -5.0 %
-5.1 to -10.0 %
-10.1 to -15.0 %
-15.1 to 18.0 %
< -18.0 %
CS Epicard
Basal
Mid
Apical
Basal -12.44 ± 9.1 %
Mid -7 ± 7.5 %
Apical -6.3 ± 5.8 %
> 0 %
0 to -5.0 %
-5.1 to -13.3 %
< -13.3 %
CS Endocard
Basal -17.88 ± 11.7 %
Mid -21.62 ± 12.4 %
Apical -25.1 ± 16.9 %
> 0 %
0 to -10.0 %
-10.1 to -20.0 %
-20.1 to -25.5 %
< -25.5 %
Radial
Basal 49.92 ± 3.4 %
Mid 47.03 ± 5.3 %
Apical 49.12 ± 1.5 %
normal
pathological
> 44.2 %
Rotation
Basal -7.48 ± 4.8 %
Apical -4.24 ± 3.1 %
normal
pathological
basal < -3.0 %
apical < 3.0 %
not used

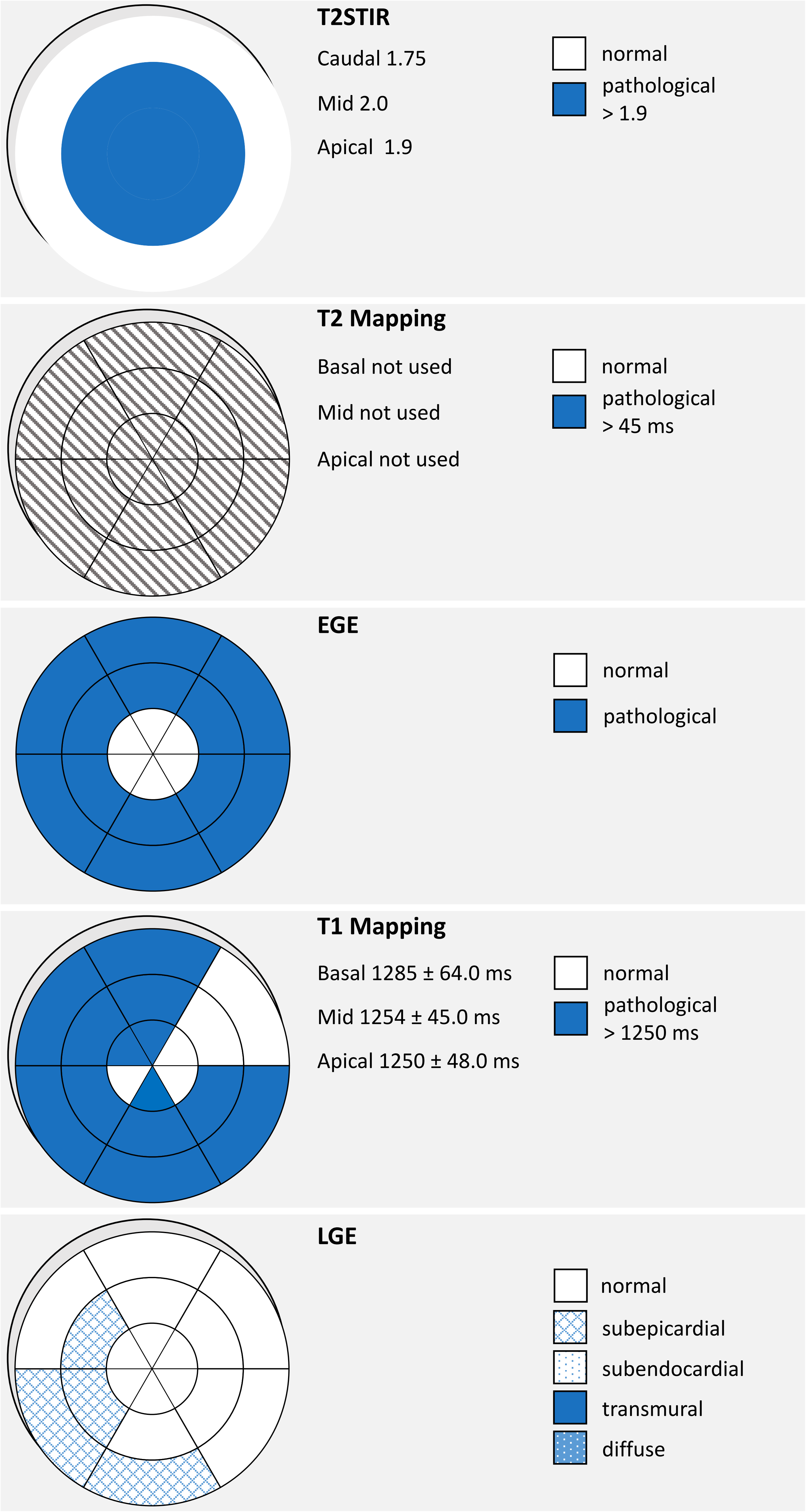

T2STIR
Caudal 1.75
Mid 2.0
Apical 1.9
normal
pathological > 1.9
T2 Mapping
Basal not used
Mid not used
Apical not used
normal
pathological > 45 ms
EGE
normal
pathological
T1 Mapping
Basal 1285 ± 64.0 ms
Mid 1254 ± 45.0 ms
Apical 1250 ± 48.0 ms
normal
pathological > 1250 ms
LGE
normal
subepicardial
subendocardial
transmural
diffuse

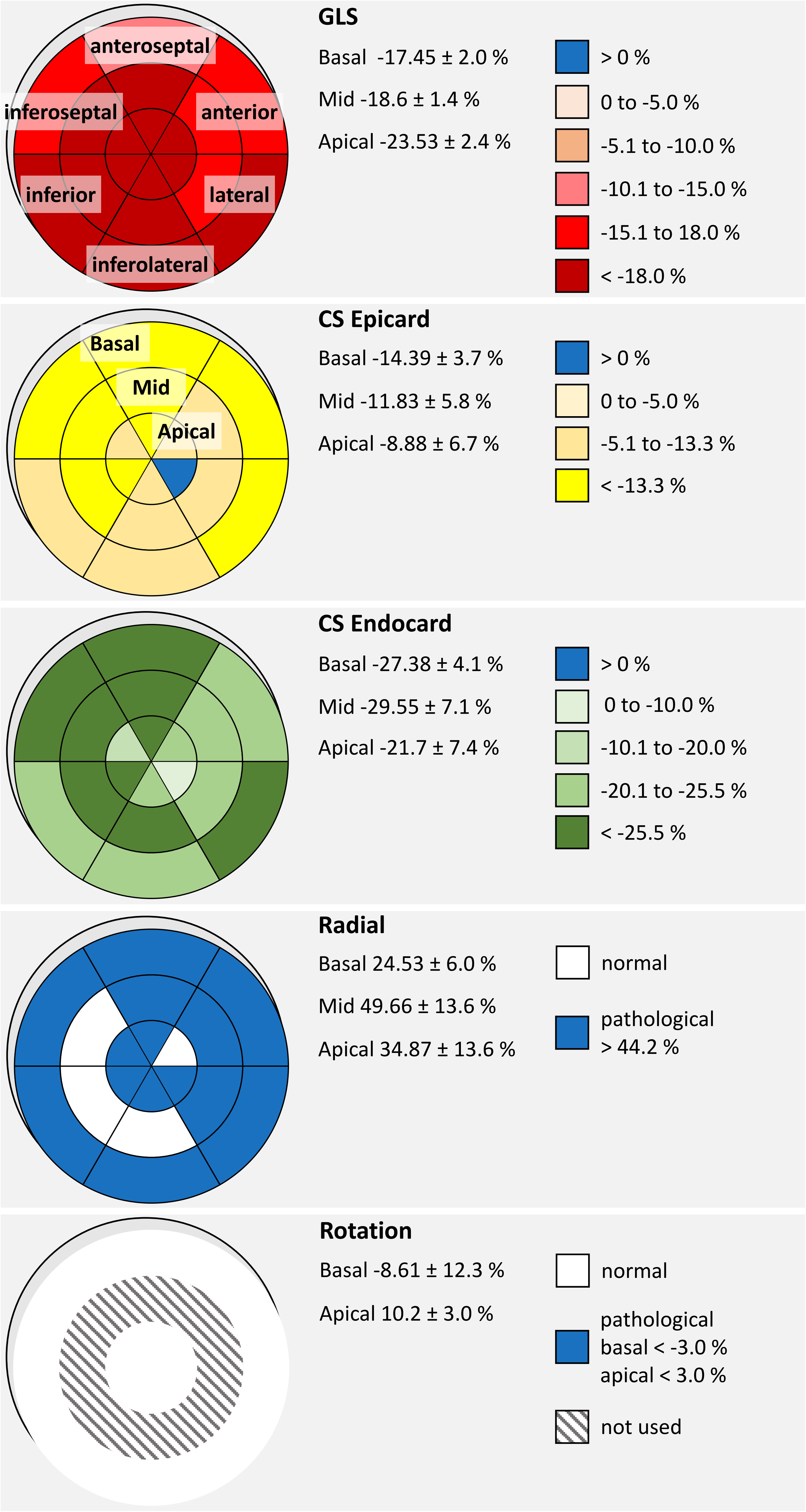
anteroseptal
inferoseptal
anterior
inferior
lateral
inferolateral
GLS
Basal -17.45 ± 2.0 %
Mid -18.6 ± 1.4 %
Apical -23.53 ± 2.4 %
> 0 %
0 to -5.0 %
-5.1 to -10.0 %
-10.1 to -15.0 %
-15.1 to 18.0 %
< -18.0 %
Basal
Mid
Apical
CS Epicard
Basal -14.39 ± 3.7 %
Mid -11.83 ± 5.8 %
Apical -8.88 ± 6.7 %
> 0 %
0 to -5.0 %
-5.1 to -13.3 %
< -13.3 %
CS Endocard
Basal -27.38 ± 4.1 %
Mid -29.55 ± 7.1 %
Apical -21.7 ± 7.4 %
> 0 %
0 to -10.0 %
-10.1 to -20.0 %
-20.1 to -25.5 %
< -25.5 %
Radial
Basal 24.53 ± 6.0 %
Mid 49.66 ± 13.6 %
Apical 34.87 ± 13.6 %
normal
pathological
> 44.2 %
Rotation
Basal -8.61 ± 12.3 %
Apical 10.2 ± 3.0 %
normal
pathological
basal < -3.0 %
apical < 3.0 %
not used

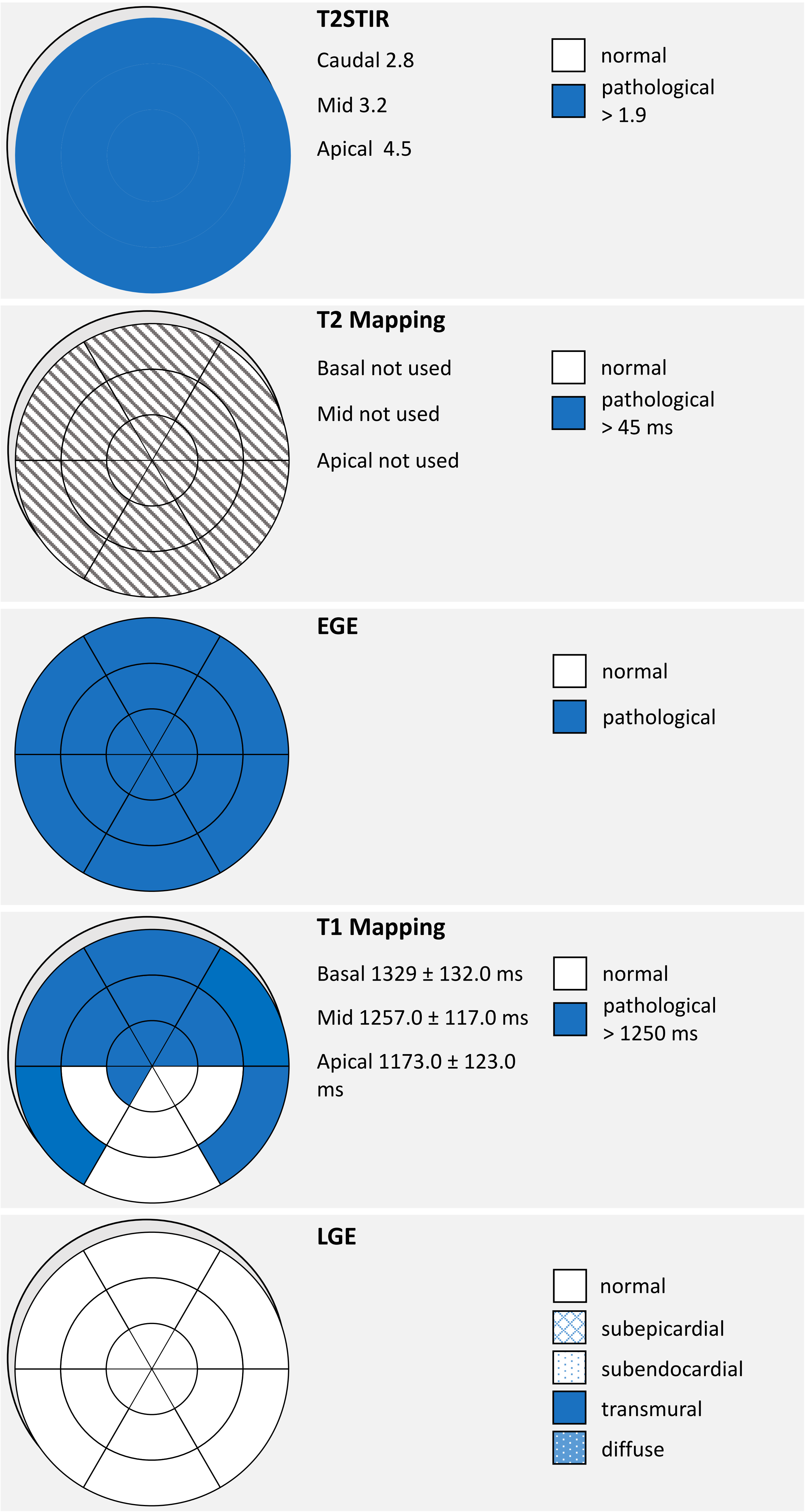
T2STIR
Caudal 2.8
Mid 3.2
Apical 4.5
normal
pathological > 1.9
T2 Mapping
Basal not used
Mid not used
Apical not used
normal
pathological > 45 ms
EGE
normal
pathological
T1 Mapping
Basal 1329 ± 132.0 ms
Mid 1257.0 ± 117.0 ms
Apical 1173.0 ± 123.0 ms
normal
pathological > 1250 ms
LGE
normal
subepicardial
subendocardial
transmural
diffuse

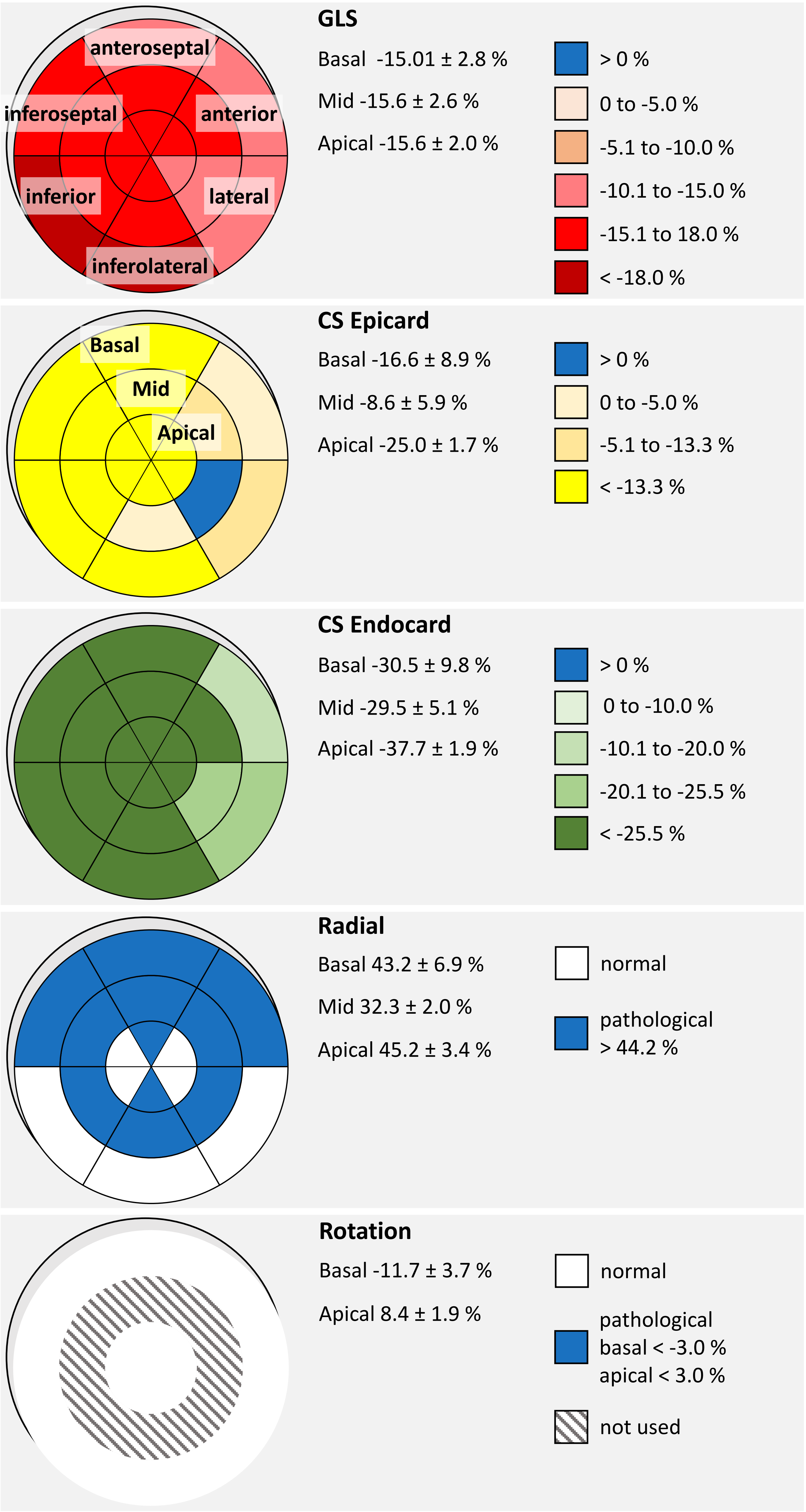
anteroseptal
inferoseptal
anterior
inferior
lateral
inferolateral
GLS
Basal -15.01 ± 2.8 %
Mid -15.6 ± 2.6 %
Apical -15.6 ± 2.0 %
> 0 %
0 to -5.0 %
-5.1 to -10.0 %
-10.1 to -15.0 %
-15.1 to 18.0 %
< -18.0 %
Basal
Mid
Apical
CS Epicard
Basal -16.6 ± 8.9 %
Mid -8.6 ± 5.9 %
Apical -25.0 ± 1.7 %
> 0 %
0 to -5.0 %
-5.1 to -13.3 %
< -13.3 %
CS Endocard
Basal -30.5 ± 9.8 %
Mid -29.5 ± 5.1 %
Apical -37.7 ± 1.9 %
> 0 %
0 to -10.0 %
-10.1 to -20.0 %
-20.1 to -25.5 %
< -25.5 %
Radial
Basal 43.2 ± 6.9 %
Mid 32.3 ± 2.0 %
Apical 45.2 ± 3.4 %
normal
pathological
> 44.2 %
Rotation
Basal -11.7 ± 3.7 %
Apical 8.4 ± 1.9 %
normal
pathological
basal < -3.0 %
apical < 3.0 %
not used

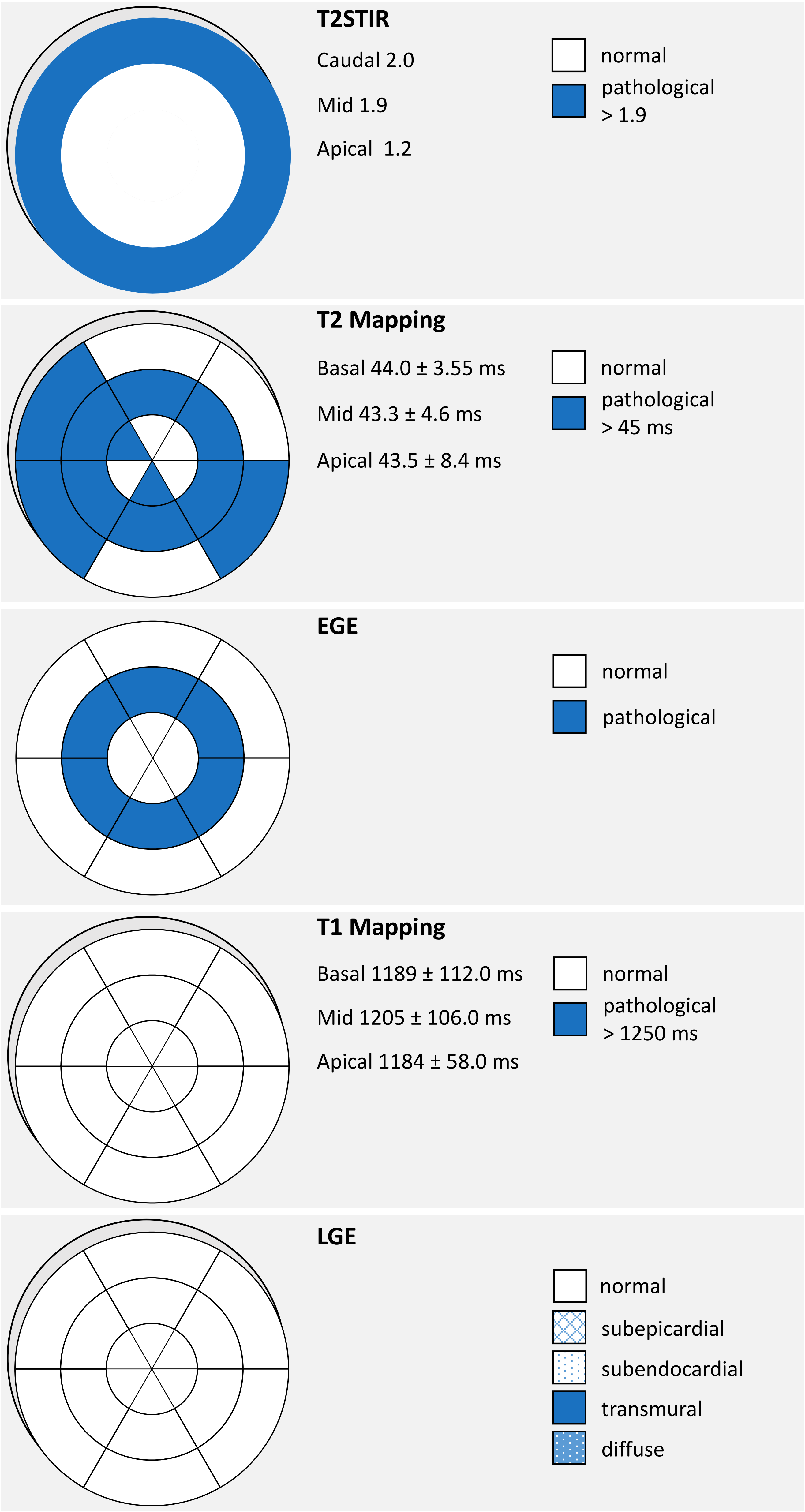
T2STIR
Caudal 2.0
Mid 1.9
Apical 1.2
normal
pathological
> 1.9
T2 Mapping
Basal 44.0 ± 3.55 ms
Mid 43.3 ± 4.6 ms
Apical 43.5 ± 8.4 ms
normal
pathological
> 45 ms
EGE
normal
pathological
T1 Mapping
Basal 1189 ± 112.0 ms
Mid 1205 ± 106.0 ms
Apical 1184 ± 58.0 ms
normal
pathological
> 1250 ms
LGE
normal
subepicardial
subendocardial
transmural
diffuse

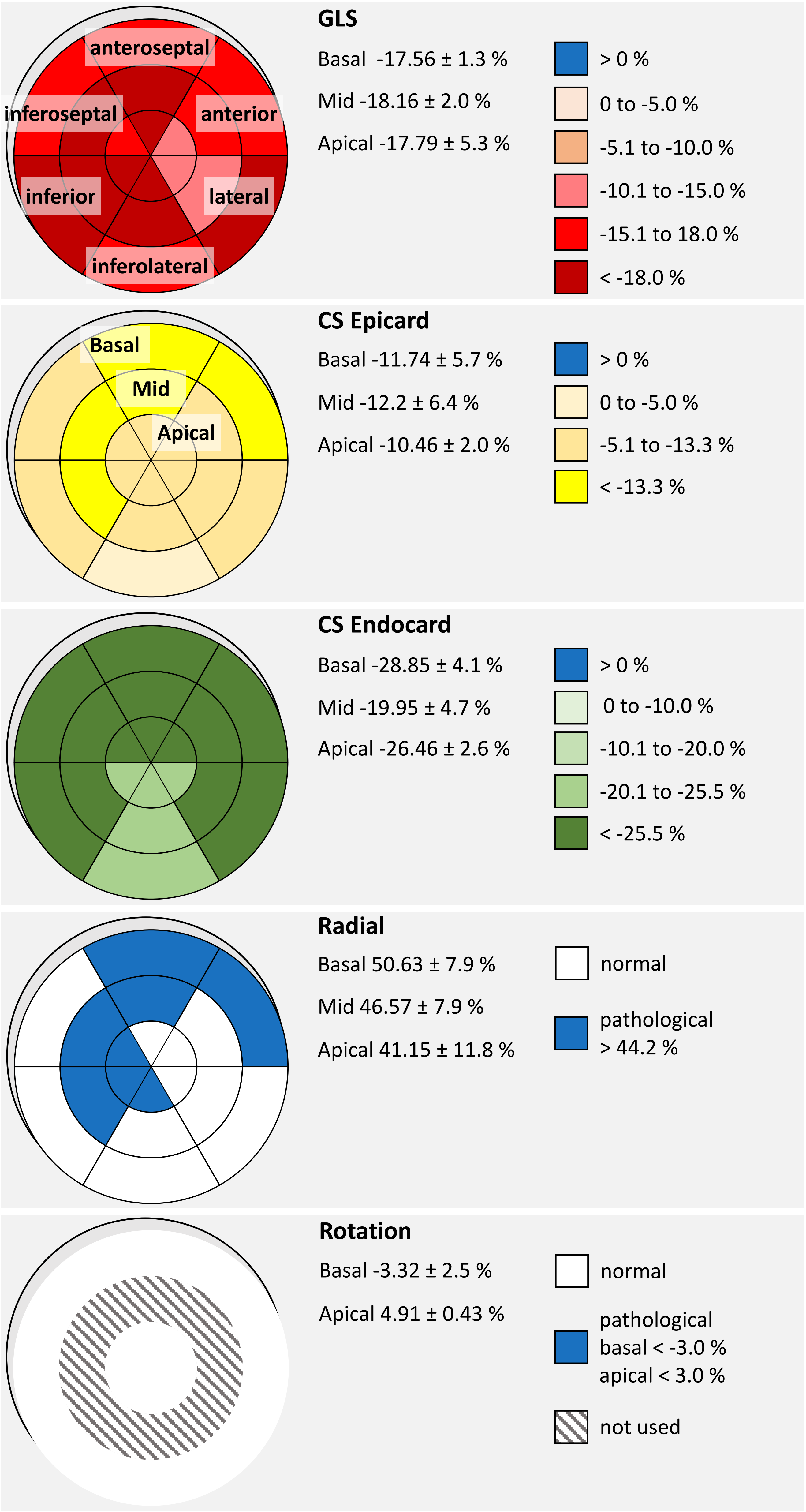
GLS
anteroseptal
inferoseptal
anterior
inferior
lateral
inferolateral
Basal -17.56 ± 1.3 %
Mid -18.16 ± 2.0 %
Apical -17.79 ± 5.3 %
> 0 %
0 to -5.0 %
-5.1 to -10.0 %
-10.1 to -15.0 %
-15.1 to 18.0 %
< -18.0 %
CS Epicard
Basal
Mid
Apical
Basal -11.74 ± 5.7 %
Mid -12.2 ± 6.4 %
Apical -10.46 ± 2.0 %
> 0 %
0 to -5.0 %
-5.1 to -13.3 %
< -13.3 %
CS Endocard
Basal -28.85 ± 4.1 %
Mid -19.95 ± 4.7 %
Apical -26.46 ± 2.6 %
> 0 %
0 to -10.0 %
-10.1 to -20.0 %
-20.1 to -25.5 %
< -25.5 %
Radial
Basal 50.63 ± 7.9 %
Mid 46.57 ± 7.9 %
Apical 41.15 ± 11.8 %
normal
pathological
> 44.2 %
Rotation
Basal -3.32 ± 2.5 %
Apical 4.91 ± 0.43 %
normal
pathological
basal < -3.0 %
apical < 3.0 %
not used

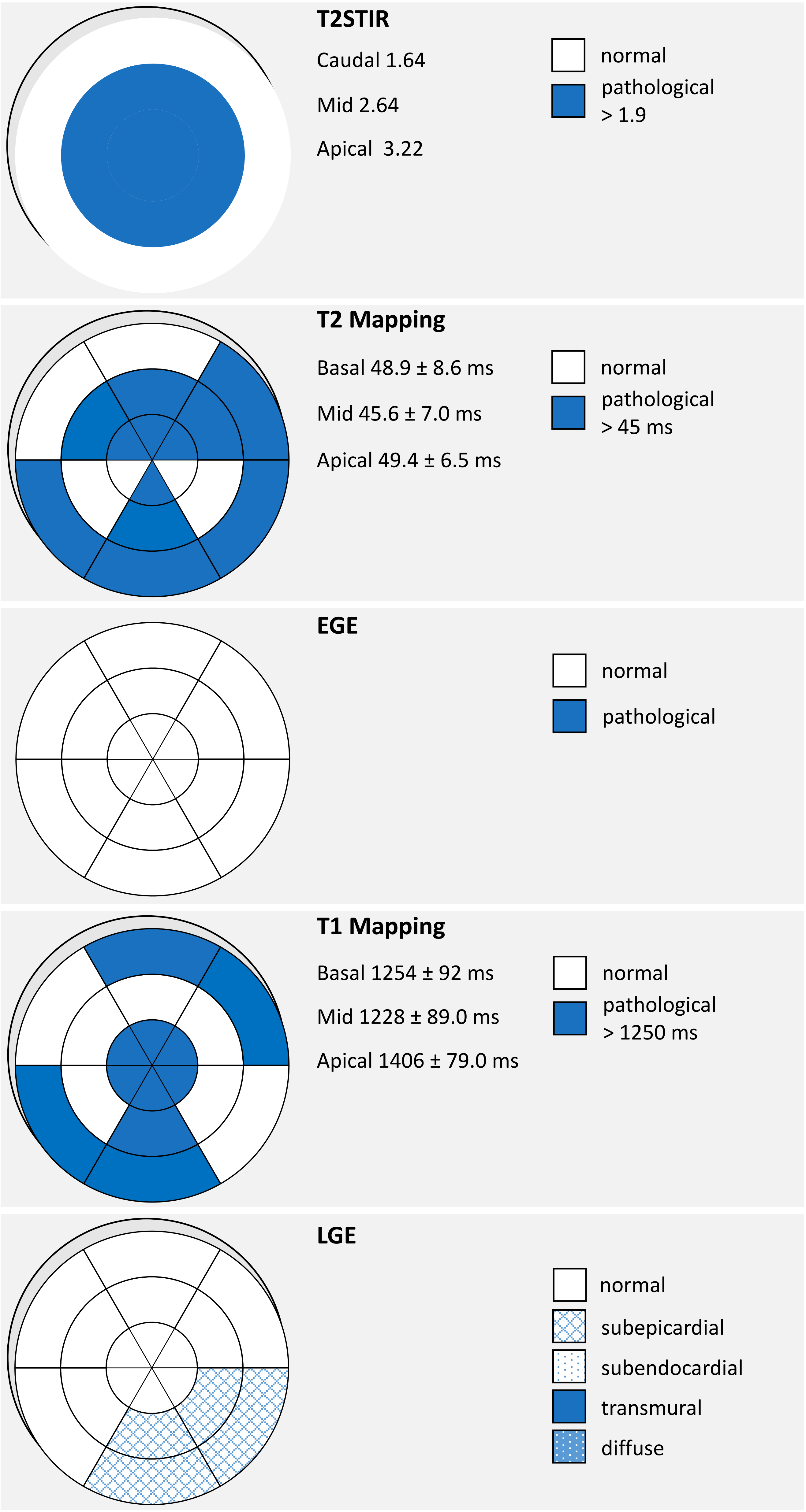
T2STIR
Caudal 1.64
Mid 2.64
Apical 3.22
normal
pathological
> 1.9
T2 Mapping
Basal 48.9 ± 8.6 ms
Mid 45.6 ± 7.0 ms
Apical 49.4 ± 6.5 ms
normal
pathological
> 45 ms
EGE
normal
pathological
T1 Mapping
Basal 1254 ± 92 ms
Mid 1228 ± 89.0 ms
Apical 1406 ± 79.0 ms
normal
pathological
> 1250 ms
LGE
normal
subepicardial
subendocardial
transmural
diffuse

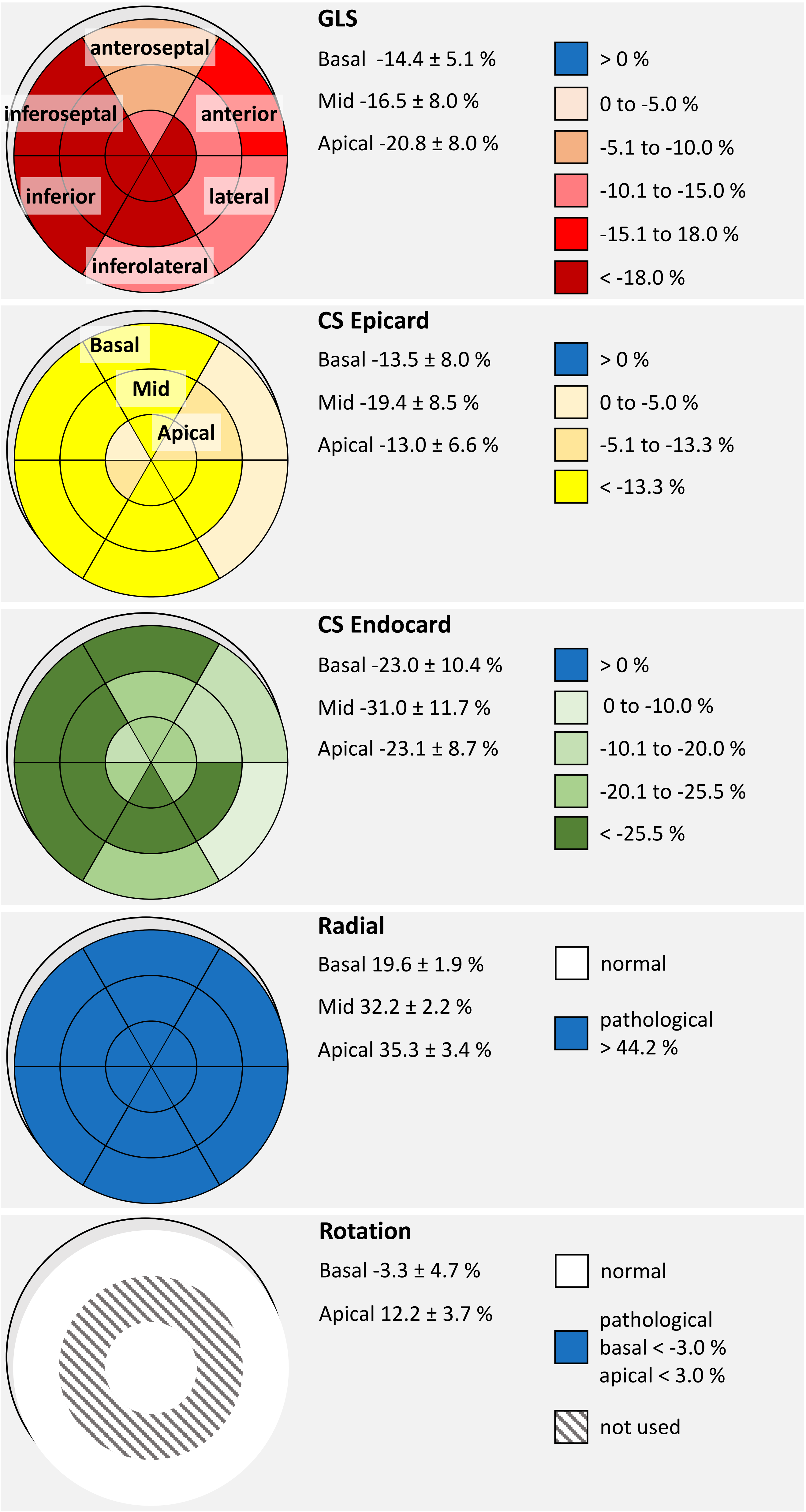

anteroseptal
inferoseptal
anterior
inferior
lateral
inferolateral
GLS
Basal -14.4 ± 5.1 %
Mid -16.5 ± 8.0 %
Apical -20.8 ± 8.0 %
> 0 %
0 to -5.0 %
-5.1 to -10.0 %
-10.1 to -15.0 %
-15.1 to 18.0 %
< -18.0 %
Basal
Mid
Apical
CS Epicard
Basal -13.5 ± 8.0 %
Mid -19.4 ± 8.5 %
Apical -13.0 ± 6.6 %
> 0 %
0 to -5.0 %
-5.1 to -13.3 %
< -13.3 %
CS Endocard
Basal -23.0 ± 10.4 %
Mid -31.0 ± 11.7 %
Apical -23.1 ± 8.7 %
> 0 %
0 to -10.0 %
-10.1 to -20.0 %
-20.1 to -25.5 %
< -25.5 %
Radial
Basal 19.6 ± 1.9 %
Mid 32.2 ± 2.2 %
Apical 35.3 ± 3.4 %
normal
pathological
> 44.2 %
Rotation
Basal -3.3 ± 4.7 %
Apical 12.2 ± 3.7 %
normal
pathological
basal < -3.0 %
apical < 3.0 %
not used

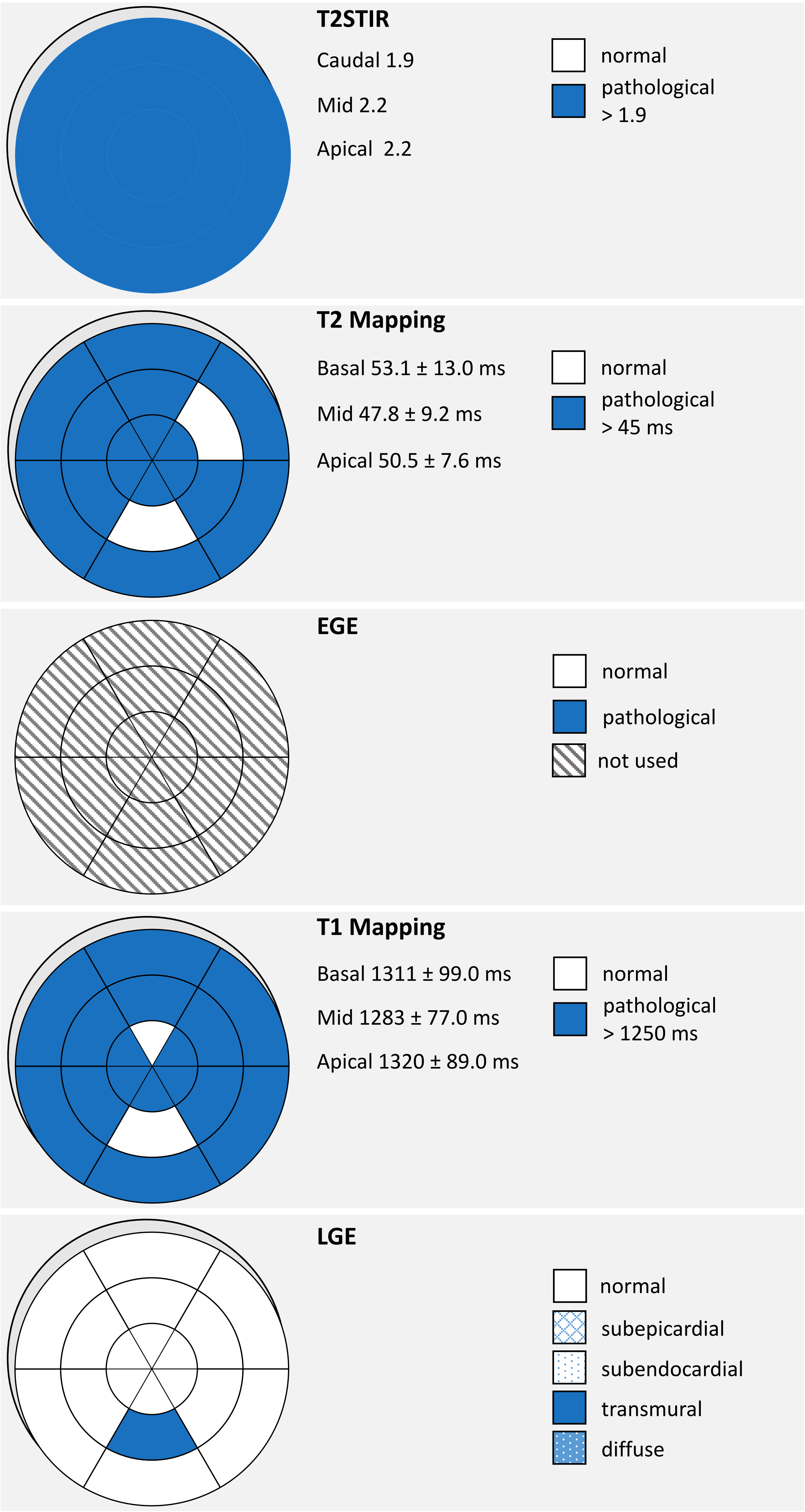
T2STIR
Caudal 1.9
Mid 2.2
Apical 2.2
normal
pathological
> 1.9
T2 Mapping
Basal 53.1 ± 13.0 ms
Mid 47.8 ± 9.2 ms
Apical 50.5 ± 7.6 ms
normal
pathological
> 45 ms
EGE
normal
pathological
not used
T1 Mapping
Basal 1311 ± 99.0 ms
Mid 1283 ± 77.0 ms
Apical 1320 ± 89.0 ms
normal
pathological
> 1250 ms
LGE
normal
subepicardial
subendocardial
transmural
diffuse

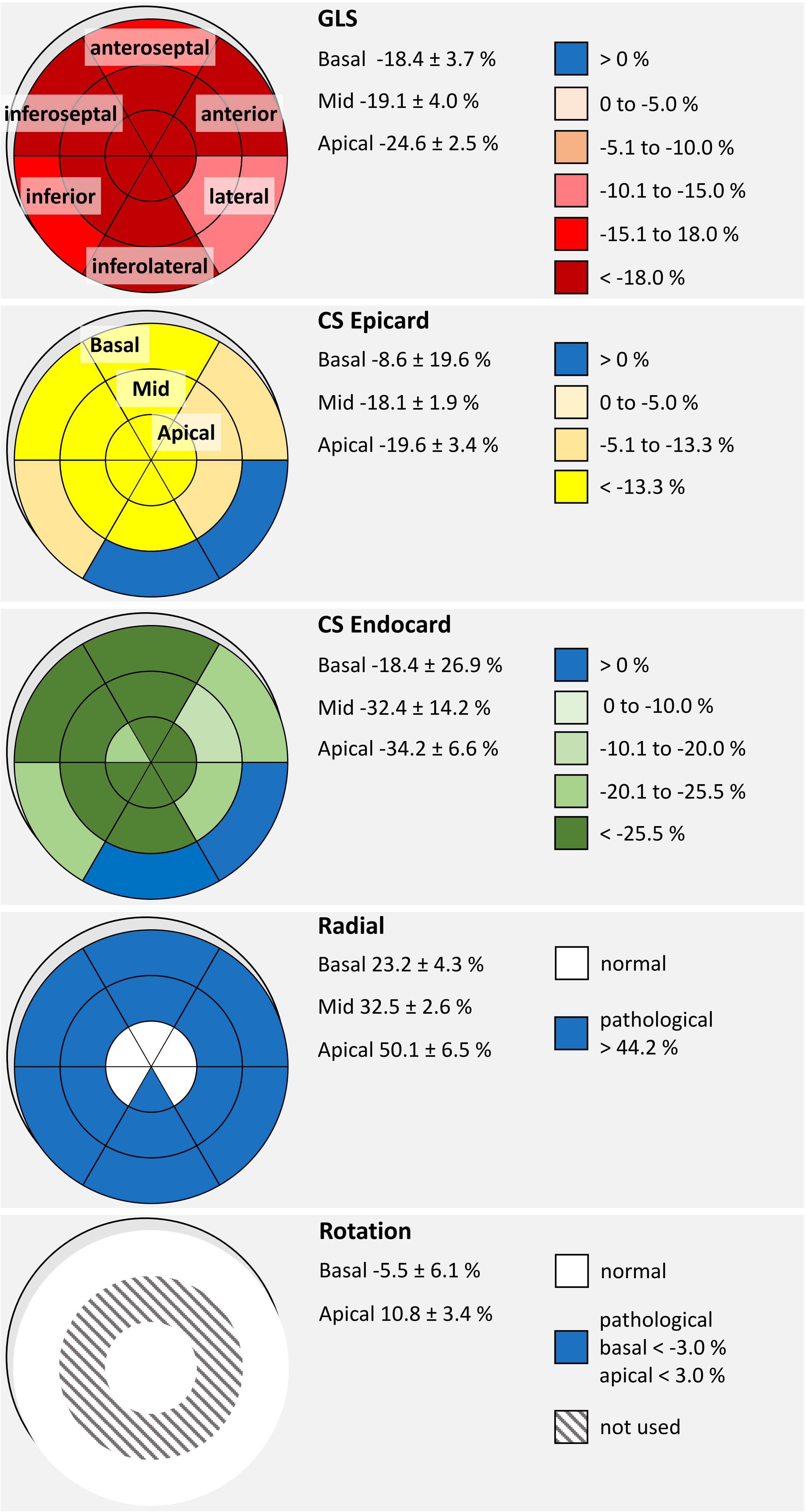
GLS
Basal -18.4 ± 3.7 %
Mid -19.1 ± 4.0 %
Apical -24.6 ± 2.5 %
anteroseptal
inferoseptal
anterior
inferior
lateral
inferolateral
> 0 %
0 to -5.0 %
-5.1 to -10.0 %
-10.1 to -15.0 %
-15.1 to 18.0 %
< -18.0 %
CS Epicard
Basal -8.6 ± 19.6 %
Mid -18.1 ± 1.9 %
Apical -19.6 ± 3.4 %
Basal
Mid
Apical
> 0 %
0 to -5.0 %
-5.1 to -13.3 %
< -13.3 %
CS Endocard
Basal -18.4 ± 26.9 %
Mid -32.4 ± 14.2 %
Apical -34.2 ± 6.6 %
> 0 %
0 to -10.0 %
-10.1 to -20.0 %
-20.1 to -25.5 %
< -25.5 %
Radial
Basal 23.2 ± 4.3 %
Mid 32.5 ± 2.6 %
Apical 50.1 ± 6.5 %
normal
pathological > 44.2 %
Rotation
Basal -5.5 ± 6.1 %
Apical 10.8 ± 3.4 %
normal
pathological basal < -3.0 % apical < 3.0 %
not used

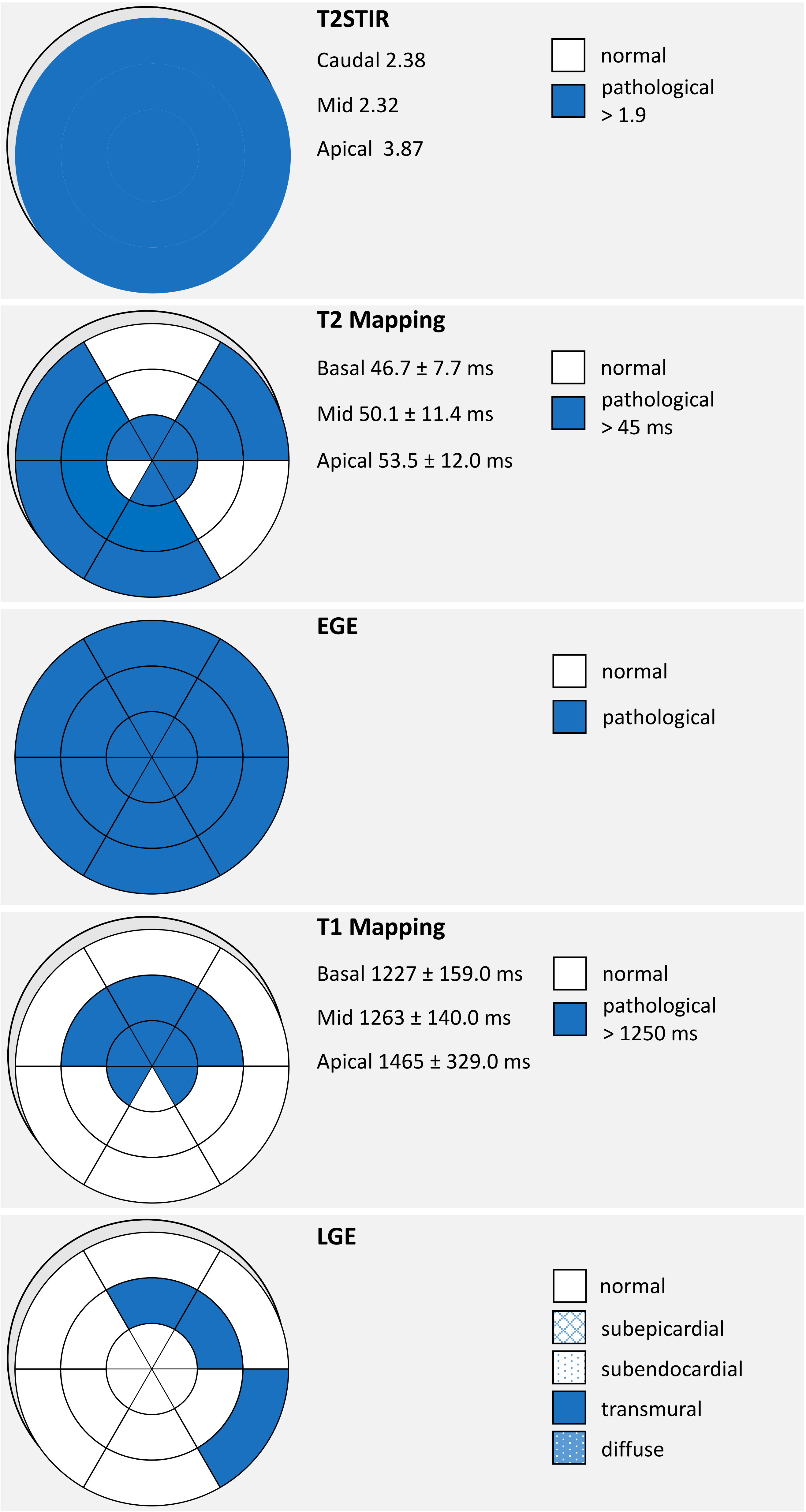
T2STIR
Caudal 2.38
Mid 2.32
Apical 3.87
normal
pathological
> 1.9
T2 Mapping
Basal 46.7 ± 7.7 ms
Mid 50.1 ± 11.4 ms
Apical 53.5 ± 12.0 ms
normal
pathological
> 45 ms
EGE
normal
pathological
T1 Mapping
Basal 1227 ± 159.0 ms
Mid 1263 ± 140.0 ms
Apical 1465 ± 329.0 ms
normal
pathological
> 1250 ms
LGE
normal
subepicardial
subendocardial
transmural
diffuse

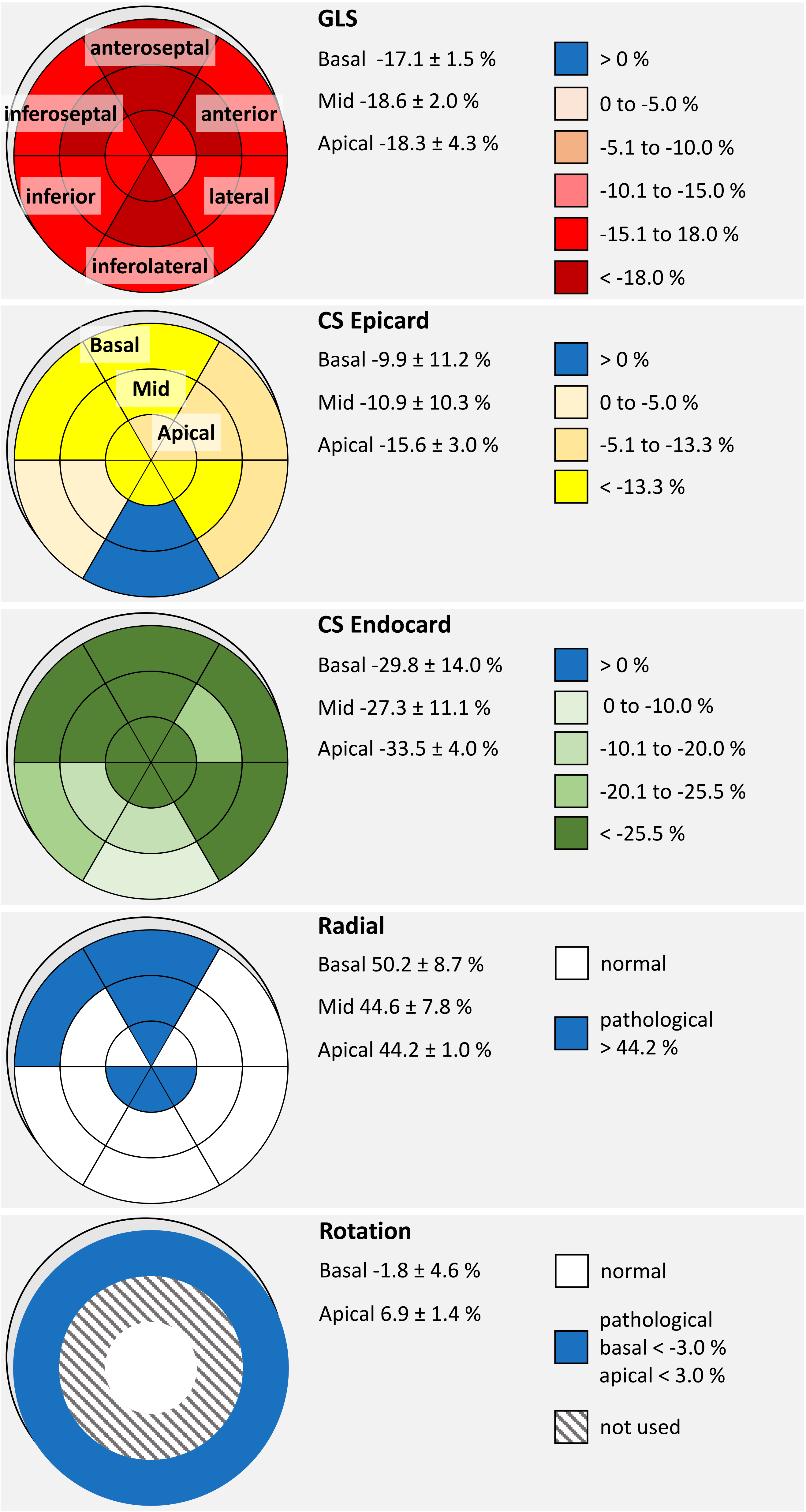

anteroseptal
inferoseptal
anterior
inferior
lateral
inferolateral
GLS
Basal -17.1 ± 1.5 %
Mid -18.6 ± 2.0 %
Apical -18.3 ± 4.3 %
> 0 %
0 to -5.0 %
-5.1 to -10.0 %
-10.1 to -15.0 %
-15.1 to 18.0 %
< -18.0 %
Basal
Mid
Apical
CS Epicard
Basal -9.9 ± 11.2 %
Mid -10.9 ± 10.3 %
Apical -15.6 ± 3.0 %
> 0 %
0 to -5.0 %
-5.1 to -13.3 %
< -13.3 %
CS Endocard
Basal -29.8 ± 14.0 %
Mid -27.3 ± 11.1 %
Apical -33.5 ± 4.0 %
> 0 %
0 to -10.0 %
-10.1 to -20.0 %
-20.1 to -25.5 %
< -25.5 %
Radial
Basal 50.2 ± 8.7 %
Mid 44.6 ± 7.8 %
Apical 44.2 ± 1.0 %
normal
pathological
> 44.2 %
Rotation
Basal -1.8 ± 4.6 %
Apical 6.9 ± 1.4 %
normal
pathological
basal < -3.0 %
apical < 3.0 %
not used

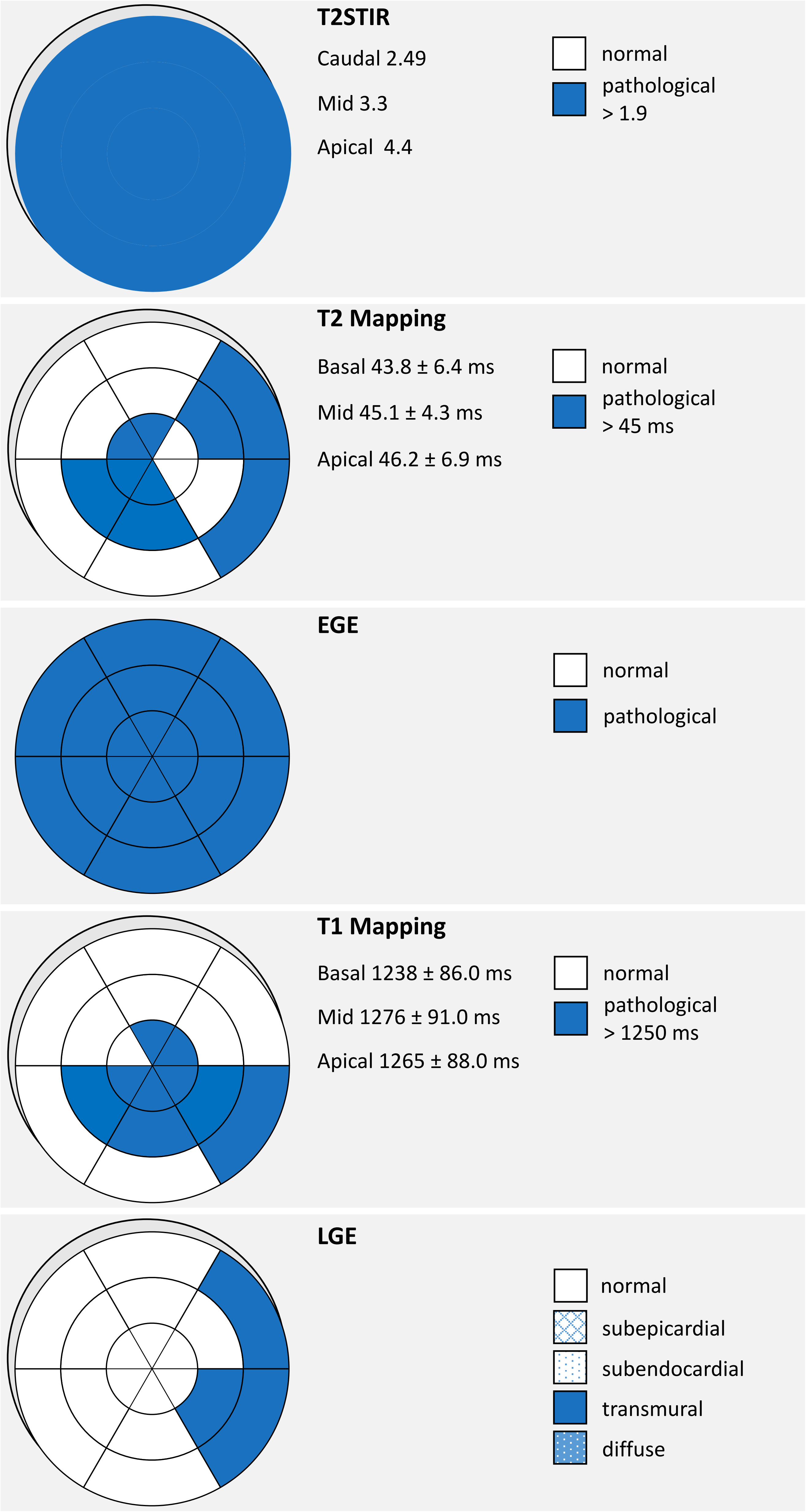
T2STIR
Caudal 2.49
Mid 3.3
Apical 4.4
normal
pathological > 1.9
T2 Mapping
Basal 43.8 ± 6.4 ms
Mid 45.1 ± 4.3 ms
Apical 46.2 ± 6.9 ms
normal
pathological > 45 ms
EGE
normal
pathological
T1 Mapping
Basal 1238 ± 86.0 ms
Mid 1276 ± 91.0 ms
Apical 1265 ± 88.0 ms
normal
pathological > 1250 ms
LGE
normal
subepicardial
subendocardial
transmural
diffuse

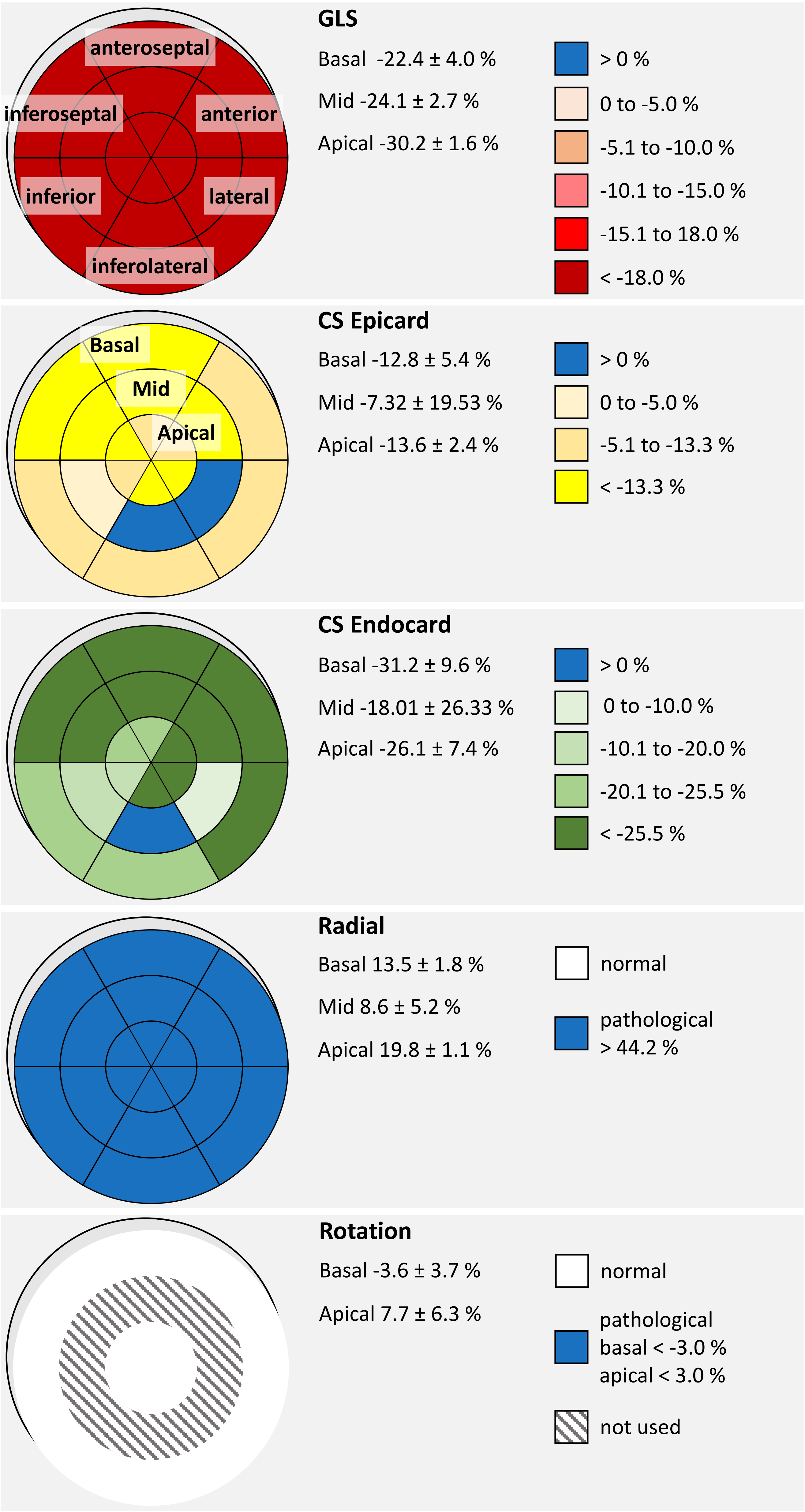
GLS
anteroseptal
inferoseptal
anterior
inferior
lateral
inferolateral
Basal -22.4 ± 4.0 %
Mid -24.1 ± 2.7 %
Apical -30.2 ± 1.6 %
> 0 %
0 to -5.0 %
-5.1 to -10.0 %
-10.1 to -15.0 %
-15.1 to 18.0 %
< -18.0 %
CS Epicard
Basal
Mid
Apical
Basal -12.8 ± 5.4 %
Mid -7.32 ± 19.53 %
Apical -13.6 ± 2.4 %
> 0 %
0 to -5.0 %
-5.1 to -13.3 %
< -13.3 %
CS Endocard
Basal -31.2 ± 9.6 %
Mid -18.01 ± 26.33 %
Apical -26.1 ± 7.4 %
> 0 %
0 to -10.0 %
-10.1 to -20.0 %
-20.1 to -25.5 %
< -25.5 %
Radial
Basal 13.5 ± 1.8 %
Mid 8.6 ± 5.2 %
Apical 19.8 ± 1.1 %
normal
pathological
> 44.2 %
Rotation
Basal -3.6 ± 3.7 %
Apical 7.7 ± 6.3 %
normal
pathological
basal < -3.0 %
apical < 3.0 %
not used

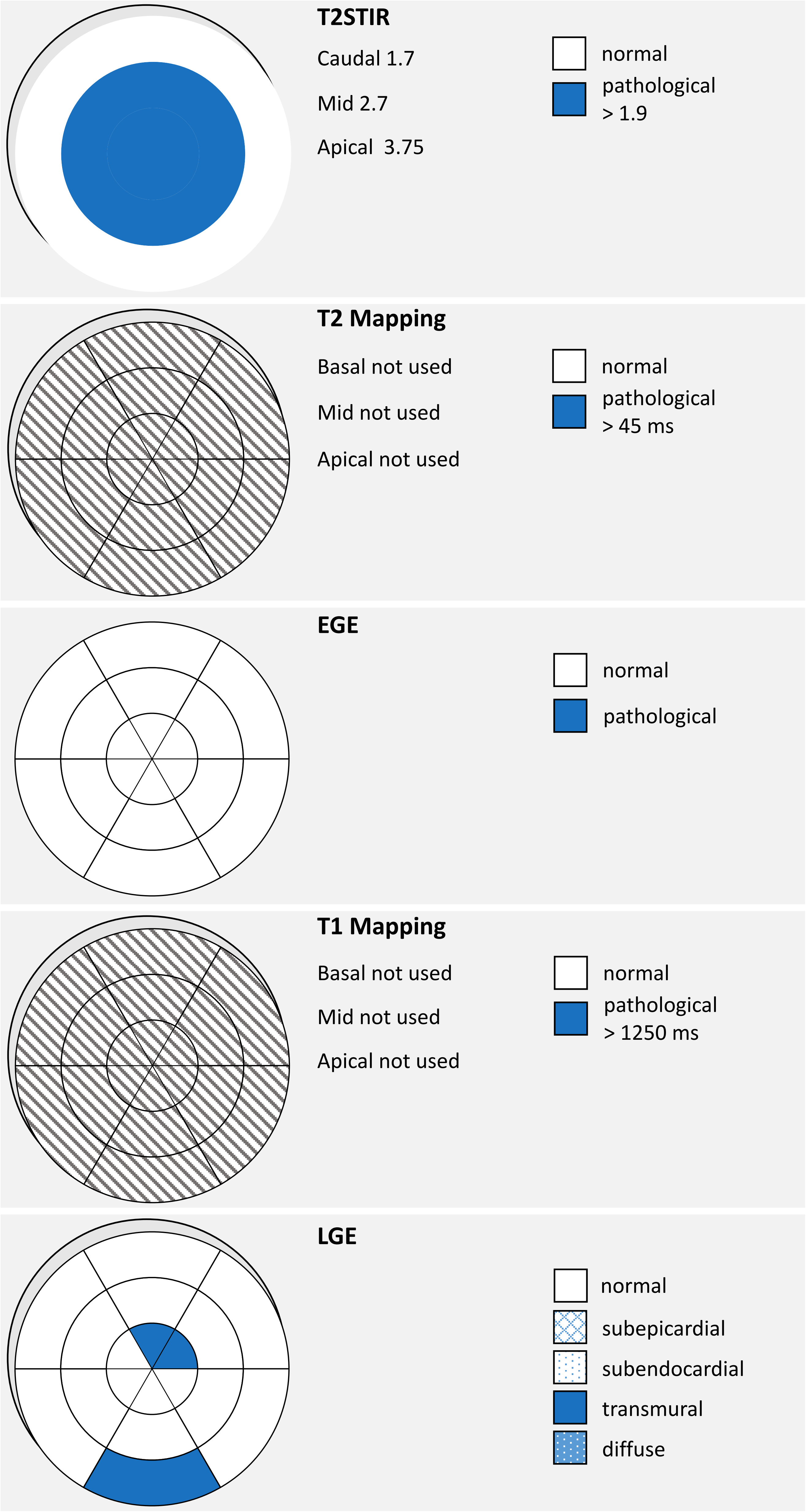
T2STIR
Caudal 1.7
Mid 2.7
Apical 3.75
normal
pathological > 1.9
T2 Mapping
Basal not used
Mid not used
Apical not used
normal
pathological > 45 ms
EGE
normal
pathological
T1 Mapping
Basal not used
Mid not used
Apical not used
normal
pathological > 1250 ms
LGE
normal
subepicardial
subendocardial
transmural
diffuse
