# Supplementary material for: Comparison of left ventricular deformation abnormalities by echocardiography with cardiac magnetic resonance imaging in patients with acute myocarditis and preserved left ventricular ejection fraction
Source: Front Cardiovasc Med. 2024 Jan 9;10:1322145. doi: 10.3389/fcvm.2023.1322145 (PMC10803407; doi:10.3389/fcvm.2023.1322145)
Supplement: Supplementary file 2 [file Table1.docx]

**Supplementary tables**

**Table 1**

| **T2-STIR** | **Basal**  (mean value ± SD) | **Mid**  (mean value ± SD) | **Apical**  (mean value ± SD) | ***P* value** |
| --- | --- | --- | --- | --- |
| **T2STIR** | | | | |
|  | 2.3 ± 0.7† | 2.6 ± 1.0 | 2.8 ± 1.1 | 0.168 |
| **Native T2 Mapping** | | | | |
| anterior | 52.2 ± 8.8* | 47.9 ± 7.4 | 48.7 ± 6.5 | 0.103 |
| lateral | 48.3 ± 6.6 | 48.5 ± 5.7 | 50.3 ± 6.7 | 0.461 |
| inferolateral | 47.0 ± 5.9*† | 49.7 ± 5.6 | 51.7 ± 6.7 | **0.024** |
| inferior | 51.1 ± 7.5 | 50.0 ± 6.6 | 49.7 ± 4.7 | 0.707 |
| septal | 49.7 ± 7.5 | 49.1 ± 5.5 | 50.1 ± 3.9 | 0.998 |
| anteroseptal | 47.2 ± 4.6 | 47.3 ± 6.9 | 48.4 ± 6.3 | 0.728 |
| **EGE** | | | | |
| Normal | 10 | 10 | 10 | 1 |
| Pathological | 12 | 12 | 11 | 0.921 |
| **Native T1 Mapping** | | | | |
| anterior | 1301.6 ± 104.5 | 1285.8 ± 119.3 | 1336.8 ± 120.4 | 0.268 |
| lateral | 1281.1 ± 73.1† | 1287.4 ± 78.8 | 1330.8 ± 106.5 | 0.089 |
| inferolateral | 1290.0 ± 80.7† | 1284.5 ± 78.7 | 1324.4 ± 85.0 | **0.030** |
| inferior | 1316.0 ± 80.7 | 1302.3 ± 89.4 | 1344.0 ± 96.8 | 0.235 |
| septal | 1291.0 ± 76.6 | 1296.1 ± 82.4 | 1316.2 ± 95.4 | 0.532 |
| anteroseptal | 1293.9 ± 89.5 | 1305.2 ± 92.2 | 1328.8 ± 94.6 | 0.383 |
| **Longitudinal strain** | | | | |
| anterior | -16.7 ± 4.9 | -16.5 ± 5.8 | -19.0 ± 8.1 | 0.301 |
| lateral | -15.6 ± 4.7† | -14.4 ± 8.3† | -18.6 ± 7.5 | 0.091 |
| inferolateral | -18.0 ± 4.3 | -17.6 ± 4.3 | -18.2 ± 6.9 | 0.999 |
| inferior | -18.0 ± 3.8† | -19.2 ± 4.1† | -22.8 ± 6.3 | 0.002 |
| inferoseptal | -15.5 ± 2.7*† | -18.1 ± 3.3† | -21.7 ± 6.6 | **<0.001** |
| anteroseptal | -15.8 ± 3.7 | -17.3 ± 5.5 | -18.5 ± 8.6 | 0.304 |
| **Subepicardial circumferential strain** | | | | |
| anterior | -8.0 ± 7.0 | -7.0 ± 8.2 | -7.1 ± 9.5 | 0.712 |
| lateral | -6.1 ± 7.7† | -6.3 ± 7.3 | -10.3 ± 7.1 | 0.075 |
| inferolateral | -5.5 ± 10.9† | -4.4 ± 16.4† | -12.8 ± 9.5 | **0.039** |
| inferior | -10.5 ± 10.5 | -12.9 ± 9.4 | -14.2 ± 8.4 | 0.365 |
| inferoseptal | -17.0 ± 7.2† | -18.0 ± 7.2† | -10.4 ± 7.2 | **<0.001** |
| anteroseptal | -18.9 ± 7.7† | -17.1 ± 6.0† | -9.0 ± 6.9 | **<0.001** |
| **Subendocardial circumferential strain** | | | | |
| anterior | -22.9 ± 9.3 | -21.8 ± 12.5 | -23.1 ± 10.6 | 0.998 |
| lateral | -20.3 ± 10.7† | -19.7 ± 9.1† | -25.4 ± 10.0 | 0.083 |
| inferolateral | -18.3 ± 16.6† | -19.7 ± 22.8† | -29.3 ± 11.4 | 0.055 |
| inferior | -21.0 ± 19.8*† | -29.4 ± 12.1 | -29.6 ± 11.8 | 0.069 |
| inferoseptal | -32.1 ± 8.4† | -35.7 ± 10.3† | -25.8 ± 11.4 | 0.003 |
| anteroseptal | -34.5 ± 9.9† | -34.2 ± 8.7† | -24.6 ± 11.0 | **<0.001** |
| **Radial strain** | | | | |
| anterior | 38.6 ± 17.2 | 38.8 ± 17.3 | 42.4 ± 18.2 | 0.682 |
| lateral | 39.3 ± 20.4 | 42.1 ± 16.1 | 43.2 ± 20.0 | 0.747 |
| inferolateral | 38.6 ± 16.1 | 43.8 ± 16.6 | 41.6 ± 19.9 | 0.567 |
| inferior | 37.0 ± 13.2 | 42.6 ± 18.8 | 39.4 ± 18.6 | 0.497 |
| inferoseptal | 33.9 ± 13.2 | 39.0 ± 19.2 | 39.1 ± 20.0 | 0.485 |
| anteroseptal | 33.8 ± 11.5 | 36.9 ± 17.6 | 37.5 ± 19.6 | 0.690 |

*Significant difference (p < 0.05) with mid segment group. †Significant difference with apical segment group

**Table 2** Mean values of LV rotation of basal and apical LV segments

| **Rotation** | **Basal**  (mean value ± SD) | **Apical**  (mean value ± SD) | ***P* value** |
| --- | --- | --- | --- |
| anterior | -2.5 ± 3.9 | 5.1 ± 4.2 | **<0.001** |
| lateral | -4.3 ± 4.6 | 5.1 ± 4.9 | **<0.001** |
| inferolateral | -7.2 ± 4.0 | 5.2 ± 5.9 | **<0.001** |
| inferior | -7.7 ± 5.9 | 6.1 ± 6.1 | **<0.001** |
| septal | -7.3 ± 4.1 | 6.9 ± 5.4 | **<0.001** |
| anteroseptal | -3.6 ± 4.2 | 6.3 ± 4.8 | **<0.001** |

*****Significant difference (p < 0.05) with apical segment group.

**Table 3** STE and cMRI parameters (T2-STIR-ratio, Native T2- and T1-mapping) of different LV levels in controls

| **cMRI** | **basal** | **medial** | **apical** | ***P* value** |
| --- | --- | --- | --- | --- |
| T2-STIR | 1.7 ± 0.3 | 1.8 ± 0.5 | 1.7 ± 0.2 | 0.356 |
| Native T2 Mapping | 43.0 ± 1.7 | 42.5 ± 1.5 | 43.3 ± 1.2 | 0.419 |
| Native T1 Mapping | 1198 ± 19 | 1203 ± 18 | 1208 ± 22 | 0.471 |
| **TTE** |  |  |  |  |
| Longitudinal strain (%) | -22.5 ± 2.5*† | -19.3 ± 2.0† | -16.6 ± 2.1 | **<0.001** |
| Circumferential strain basal (%) | -28.2 ± 4.3*† | -18.0 ± 3.0† | -10.6 ± 3.0 | **<0.001** |
| Circumferential strain apical (%) | -33.9 ± 4.4*† | -22.4 ± 3.0† | -14.8 ± 3.3 | **<0.001** |
|  | **basal** | **apical** | |  |
| Radial strain (%) | 40.9 ± 16.5 | 31.3 ± 10.8 | | **0.010** |
| Rotation (%) | -5.6 ± 3.0 | 7.6 ± 2.6 | | **<0.001** |

*Significant difference (p < 0.05) with mid segment group. †Significant difference with epi segment group
